# Systematic Identification of Culture Conditions for Induction and Maintenance of Naive Human Pluripotency

**DOI:** 10.1016/j.stem.2014.08.002

**Published:** 2014-10-02

**Authors:** Thorold W. Theunissen, Benjamin E. Powell, Haoyi Wang, Maya Mitalipova, Dina A. Faddah, Jessica Reddy, Zi Peng Fan, Dorothea Maetzel, Kibibi Ganz, Linyu Shi, Tenzin Lungjangwa, Sumeth Imsoonthornruksa, Yonatan Stelzer, Sudharshan Rangarajan, Ana D’Alessio, Jianming Zhang, Qing Gao, Meelad M. Dawlaty, Richard A. Young, Nathanael S. Gray, Rudolf Jaenisch

(Cell Stem Cell *15*, 471–487; October 2, 2014)

Because of a production issue with this paper, the initial version posted on July 24, 2014 was not the final approved version and contained textual errors. It was replaced by the correct version of the article on July 25, 2014.

In response to a reader query, the authors would also like to clarify that, although the graphical abstract indicates that reduction of bivalent domains was induced in 5i conditions, the actual experiment involved for this analysis also included a JNK inhibitor and thus used 6i conditions (Figure 7) as indicated in the manuscript text.

