# Supplementary material for: Systematic Identification of Culture Conditions for Induction and Maintenance of Naive Human Pluripotency
Source: Cell Stem Cell. 2014 Oct 2;15(4):471–87. doi: 10.1016/j.stem.2014.07.002 (PMC4184977; doi:10.1016/j.stem.2014.07.002)
Supplement: Document S1. Supplemental Experimental Procedures and Figures S1–S7 [file mmc1.pdf]

Supplemental Information

Systematic Identification of Culture Conditions for Induction and Maintenance of Naive Human Pluripotency

Thorold W. Theunissen[1,*], Benjamin E. Powell[1,*], Haoyi Wang[1,*], Maya Mitalipova[1], Dina A. Faddah[1,2], Jessica Reddy[1,2], Zi Peng Fan[1,3], Dorothea Maetzel[1], Kibibi Ganz[1], Linyu Shi[1], Tenzin Lungjangwa[1], Sumeth Imsoonthornruksa[1], Yonatan Stelzer[1], Sudharshan Rangarajan[1], Ana D'Alessio[1], Jianming Zhang[4], Qing Gao[1], Meelad M. Dawlaty[1], Richard A. Young[1,2], Nathanael Gray[4] and Rudolf Jaenisch[1,2,#]

Contents:

I. **Extended Experimental Procedures**

II. **Supplemental Figure and Table Legends**

III. **Supplemental Figures**

IV. **Supplemental References**

I. Extended Experimental Procedures

Gene targeting

Human ESCs and iPSCs were cultured in ROCK inhibitor Y-27632 (Stemgent, 10 μM) 24 hours prior to electroporation. Cell were harvested using 0.25% trypsin/EDTA solution (Invitrogen) and 1 x 10^7 cells resuspended in phosphate buffered saline (PBS) were electroporated with 40 μg of donor plasmids and 5 μg of each TALEN-encoding plasmid (Gene Pulser Xcell System, Bio-Rad: 250 V, 500μ F, 0.4 cm cuvettes (Costa et al., 2007)). Cells were subsequently plated on MEF feeder layers (DR4 MEFs for puromycin selection) in human ESC medium supplemented with ROCK inhibitor for the

first 24 hours. Individual colonies were picked and expanded after puromycin selection (0.5 µg/ml) 10 to 14 days after electroporation. Correctly targeted clones were confirmed by southern blot (NdeI digested) and used for the removal of floxed PGK-puro cassette. Cells were harvested using 0.25% trypsin/EDTA solution (Invitrogen) and 1×10^7 cells resuspended in PBS were electroporated with pTurbo-Cre (40 µg; GenBank accession number AF334827) (Gene Pulser Xcell System, Bio-Rad; 250 V, 500 µF, 0.4 cm cuvettes). Cells were subsequently plated on MEF feeder layers at a low density in human ESC medium supplemented with ROCK inhibitor. Individual colonies were picked 10–14 d after electroporation. The excision of PGK-puro was confirmed by Southern blot analysis.

Lentiviral infection

VSVG coated lentiviruses were generated in HEK-293 cells as described previously (Brambrink et al., 2008; Soldner et al., 2009; Soldner et al., 2011). Briefly, culture medium was changed 12 hr post-transfection and virus-containing supernatant was collected 48 to 72 hr post-transfection. Viral supernatant was filtered through a 0.45 mm filter. Virus-containing supernatants of the 2 reprogramming viruses (FUW-tetO-lox-hKLF2 and FUW-tetO-lox-hNANOG) were pooled and supplemented with the FUW-lox-M2rtTA virus and an equal volume of fresh culture medium. 1×10^6 human ESCs were seeded 24 hr before transduction in T75 flasks on matrigel in mTesr1 medium (STEMCELL Technologies). Two consecutive infections in the presence of 2 mg/ml of polybrene were performed over a period of 24 hr. Culture medium was changed 12 hr after the last infection. Five days after transduction, human ESCs were passaged using 0.25% trypsin/EDTA solution (Invitrogen) and re-plated on mitomycin C inactivated mouse embryonic fibroblast (MEF) feeder layers in conventional human ESC medium. To induce conversion to the naive state, human ESCs were trypsinized and seeded at a density of 1×10^5 cells per individual 6 well on a MEF feeder layer in the presence of ROCK inhibitor Y-27632. Medium was replaced 24 hr later with N2B27 basal medium supplemented with PD0325901 (Stemgent, 1 µM), CHIR99021 (Stemgent, 3 µM), 20 ng/mL hLIF (2i/L) and doxycycline (DOX) (Sigma-Aldrich; 2 µg/ml). OCT4-ΔPE-GFP+ human ESC colonies were picked manually within 10 days after DOX induction and

passaged using Accutase (Gibco) on a MEF feeder layer. Upon the addition of 2i/L/DOX, latent OCT4-ΔPE-GFP-negative cells could be removed almost entirely by additional treatment with 0.1 μM PD173074 and 5 μM SB431542, which effectively inhibit the signaling pathways on which primed human ESCs are reliant. These additional inhibitors also facilitated the isolation of transgene-dependent naive human ESCs from wild-type WIBR3 human ESCs.

Chemical screening

To screen for small molecules that support naive human pluripotency, doxycycline (DOX) was withdrawn from a clonal line of WIBR3 OCT4-ΔPE-GFP+ human ESCs derived in 2i/L/DOX. 24h after DOX withdrawal, cells were dissociated in 0.25% trypsin/EDTA solution (Invitrogen) and seeded at a density of 5000 cells per individual well in 96 well plates on a MEF feeder layer in 2i/L supplemented with the ROCK inhibitor Y-27632 (Stemgent, 10 μM). After an additional 24h, 2i/L medium in each individual 96 well was supplemented with a kinase inhibitor from the LINCS inhibitor library (Gray Laboratory, Dana Farber Cancer Institute, Boston, MA) at a final concentration of 1 μM. To improve experimental consistency the small molecule library was applied using the Caliper RapidPlate 96 well Liquid Handling System (Zymark, Westborough, MA). Briefly, a master plate containing 10 mM stock solution of the library in DMSO was first diluted to 100 μM in HEPES aqueous solution (daughter plate), and subsequently to 10 μM in N2B27 basal medium supplemented with 2i/L (granddaughter plate). This granddaughter plate was then diluted a further 10X in 2i/L medium, the final medium was pre-mixed and applied slowly to the 96 well assay plate. Following two medium changes during a seven day period the proportion of OCT4-ΔPE-GFP+ human ESCs in each well was assessed using the High-Throughput System on the LSRFortessa SORP (Beckton-Dickinson, San Jose, CA). To screen for small molecules that improve the proportion of viable OCT4-ΔPE-GFP+ cells, this assay was modified by the addition of 1.0 μM SB590885 to each well and inclusion of a 10 minute DAPI (Life Technologies) staining prior to high-throughput FACS analysis. Viable cells were gated from the DAPI-negative fraction and subsequently assessed for GFP status.

Flow cytometry

To assess the proportion of OCT4-ΔPE-GFP+ human ESCs, a single cell suspension was filtered, stained with DAPI (Life Technologies), and assessed on the LSR II SORP (Beckton-Dickinson, San Jose, CA) or LSRFortessa SORP (Beckton-Dickinson, San Jose, CA). On the LSR II SORP, mTomato (for detection of autofluorescence) was excited by a Coherent Compass 561 nm (25 mW) yellow/green laser and detected using a bandpass filter (emission) of 610/20, GFP was excited by a Coherent Sapphire Solid State 488 nm (100 mW) blue laser and detected using a bandpass filter (emission) of 525/50, and DAPI was excited by a Lightwave Xcyte 355 nm (60 mW) UV laser and detected using a bandpass filter (emission) of 450/50. On the LSRFortessa SORP, PE-Texas Red (for detection of autofluorescence) was excited by a Coherent Compass 561 nm (50 mW) yellow/green laser and detected using a bandpass filter (emission) of 610/20, GFP was excited by a Coherent Sapphire Solid State 488 nm (100 mW) blue laser and detected using a bandpass filter (emission) of 530/30, and DAPI was excited by a Lightwave Xcyte 355 nm (60 mW) UV laser and detected using a bandpass filter (emission) of 450/50.

Immunostaining

Immunostaining was performed according to standard protocols using the following primary antibodies: Oct-3/4 (mouse monoclonal, Santa Cruz Biotechnology); hNANOG (Cat. No. AF1997, goat polyclonal, R&D Systems); AFP (Cat. No. A8452, mouse monoclonal, Sigma); HNF4a (goat polyclonal, Santa Cruz); Nestin (Cat. No. AB5922, mouse monoclonal, Milipore); Pax6 (Cat. No. PRB-278P, rabbit polyclonal, Covance). Appropriate Alexa Fluor dye conjugated secondary antibodies (Invitrogen) were used. Nuclei were stained with DAPI (Life Technologies). Images were taken using LSM710 confocal microscope (Zeiss) or Inverted microscope (Eclipse Ti-Nikon).

Human ESC derivation

Human embryos at 8-cell or blastocyst stage produced by in vitro fertilization for clinical

purposes were obtained with informed written consent and approved by MIT Institutional review board. Embryos were thawed and cultured in Multiblast medium (Irvine Scientific) supplemented with 20% serum protein substitute (50 mg/ml Quinn's Advantage SPS, InVitro Fertilization) until day 6 in 5% O_2, 5% CO_2 and 90% N_2 gas mixture. Embryos were plated as a whole blastocyst after removal of the zona pellucida by brief incubation in protease solution (Sigma), and further cultured in optimized medium for naive human pluripotency on a MEF feeder layer. At day 6-12 after plating, inner cell mass outgrowths were passaged by brief incubation in Accutase solution and dispersed to clumps of 5-10 cells. Freshly derived naive human ESCs proliferated slowly and were passaged by Accutase to single cells and frozen and used for further analysis. Karyotype analysis was performed by the Cytogenetics Laboratory at Tufts Medical Center in Boston, MA.

Generation of naive human iPSCs

Primary C1 *OCT4-ΔPE-GFP* human iPSCs harboring proviral integrations of OCT4, SOX2 and KLF4 were differentiated into fibroblast-like cells as described previously (Xu et al., 2004). iPSCs were differentiated by embryoid body formation in fibroblast medium for 5 days and subsequently plated onto adherent tissue culture dishes and passaged according to primary fibroblast protocols using trypsin for at least four passages before the start of experiments. For the derivation of naive secondary iPSCs, secondary fibroblasts were plated at 1×10^4 per 35 mm on MEF feeder layers. Twenty four hours later, fibroblast medium was replaced by primed human ESC medium supplemented with DOX (Sigma-Aldrich; 2 μg/ml) for six days. After six days the medium was replaced with naive 5i/L/A medium supplemented with FGF2 and DOX. After day 10 post DOX induction, GFP positive naive human iPSC colonies could be readily isolated and clonally expanded in 5i/L/A medium in the absence of doxycycline.

For primary reprogramming experiments fibroblasts were seeded on gelatin at a density of 1×10^5 cells per 35 mm. Viral supernatants of tetO-hSTEMCCA-loxP and FUW-lox-M2rtTA virus were used for transduction at a 3:1 ratio. Two consecutive infections in the presence of 2 mg/ml of polybrene were performed over a period of 24 hr. Culture

medium was changed 12 hr after the last infection. 24 hours later transduced fibroblasts were trypsinized and plated at 1×10^4 per 35 mm on MEF feeder layers. Twenty four hours later, fibroblast medium was replaced by primed human ESC medium supplemented with DOX (Sigma-Aldrich; 2 µg/ml) for six days. After six days the medium was replaced with naive 5i/L/A medium supplemented with FGF2 and DOX. After day 10 post DOX naive human iPSC colonies could be readily isolated and clonally expanded in 5i/L/A medium in the absence of DOX.

RNA FISH and imaging

We performed RNA FISH as outlined previously (Faddah et al., 2013; Raj et al., 2010; Raj et al., 2008). All hybridizations were performed in solution using probes coupled to either tetramethylrhodamine (TMR) (Invitrogen), Alexa 594 (Invitrogen) or Cy5 (GE Amersham). We used TMR for the probes against human KLF4 mRNA, Alexa 594 for the probes against human OCT4, REX1, and NANOG mRNA, and Cy5 for the probes against human OCT4 mRNA. Optimal probe concentrations during hybridization were determined empirically. Imaging involved taking stacks of images spaced 0.4 µm apart using filters appropriate for DAPI, TMR, Alexa 594 and Cy5. All images were taken with a Nikon Ti-E inverted fluorescence microscope equipped with a 100X oil-immersion objective and a Photometrics Pixis 1024 CCD camera using MetaMorph software (Molecular Devices, Downington, PA). During imaging, we minimized photobleaching through the use of an oxygen-scavenging solution using glucose oxidase. We segmented the cells manually and counted the number of fluorescent spots, each of which corresponds to an individual mRNA, using a combination of a semi-automated method described in (Itzkovitz et al., 2011; Raj et al., 2008) and custom software written in MATLAB (Mathworks).

RNA Extraction and Synthetic RNA Spike-In

Total RNA and sample preparation for microarray analysis was performed as previously described (Loven et al., 2012). Briefly, 1 million naive or primed human ESCs were trypsinized and purified from GFP-labeled MEFs using the FACSAria (Beckton-

Dickinson) prior to lysis and RNA extraction. Biological duplicates were subsequently collected and homogenized in 1 ml of TRIzol Reagent (Life Technologies, 15596-026), purified using the mirVANA miRNA isolation kit (Ambion, AM1560) following the manufacturer's instructions and re-suspended in 100 ml nuclease-free water (Ambion, AM9938). Total RNA was spiked-in with ERCC RNA Spike-In Mix (Ambion, 4456740), treated with DNA-freeTM DNase I (Ambion, AM1906) and analyzed on Agilent 2100 Bioanalyzer for integrity. RNA with the RNA Integrity Number (RIN) above 9.8 was hybridized to GeneChip PrimeView Human Gene Expression Arrays (Affymetrix).

Microarray Sample Preparation and Analysis

100 ng of total RNA was used to prepare biotinylated cRNA (cRNA) according to the manufacturer's protocol (30 IVT Express Kit, Affymetrix 901228). GeneChip arrays (Primeview, Affymetrix 901837) were hybridized and scanned according to standard Affymetrix protocols. The raw data was obtained by using Affymetrix Gene Chip Operating Software using default settings. A Primeview CDF provided by Affymetrix was used to generate .CEL files. The CEL files were processed with the expresso command to convert the raw probe intensities to probeset expression values with MAS5 normalization using the standard tools available within the affy package in R. The probesets of the same gene were next collapsed into a single value to represent the gene by taking the mean value. Differential gene expression was determined using moderated t-statistic in the "limma" package (http://bioinf.wehi.edu.au/limma/) from Bioconductor (www.bioconductor.org) (Smyth, 2004). A gene was considered differentially expressed if it met the following criteria: 1) absolute log2 fold- change ≥1 between the mean expression of the two conditions, 2) adjusted p-value less than 0.1 by a moderated t-test within the limma package with BH multiple hypothesis testing correction.

We also processed expression profiles of naive human ESCs and primed human ESCs published in previous studies (Chan et al., 2013; Gafni et al., 2013; Ware et al., 2014). For the expression profiles from the naive human ESCs (GSM1139484 and

GSM1139494) and primed human ESCs (GSM1139488 and GSM1139495) published in Gafni et al., 2013, the Affymetric CDF file (version V1.r3) was used to generate .CEL files. The CEL files were processed with RMA normalization using the standard tools variable within the affy package in R. For the expression profiles from the naive human ESCs (ERR361240, ERR361242, ERR361244) and primed human ESCs (ERR361241, ERR361243, ERR361245) published in Chan et al., 2013, RNA-seq reads were aligned using the software Bowtie (Langmead et al., 2009) to NCBI build 37 (hg19) of the mouse genome with the settings: -e 70 -k 1 -m 1 - n 2. The RPKM (reads per kilobase per million) for each RefSeq gene was calculated using RPKM_count.py (v2.3.5) counting only exonic reads (-e option). For the expression profiles from the naive human ESCs and primed human ESCs published in Ware et al., 2014, the processed expression tables were downloaded from the Sage Synapse Commons Repository: https://www.synapse.org/#!Synapse:syn1447088. The probesets of the same gene were next collapsed into a single value to represent the gene by taking the mean value. The p-values for differential gene expression analysis between the naive and primed human ESCs from each study were determined using moderated t-statistic in the "limma" package (http://bioinf.wehi.edu.au/limma/) from Bioconductor (www.bioconductor.org) (Smyth, 2004). The log2 fold changes between the naive and primed human ESCs and p-values for all Refseq genes from each study were displayed in Volcano plots.

To determine gene ontology (GO) categories, up-regulated (> 3 fold) and down-regulated (> 3 fold) gene lists were subjected to DAVID bioinformatics tool (http://david.abcc.ncifcrf.gov/). Categories were subsequently sorted according to Benjamini multiple testing correction (p-value < 0.01).

To assess X-linked gene expression in female human ESCs in conventional (primed) and naive medium, the expression of individual genes was compared with the average of three male control human iPSC samples previously analyzed on the same platform (GSM1142930, GSM1142931, GSM1142932). Moving average plots were generated as described previously (Bruck and Benvenisty, 2011).

Cross-Species Gene Expression Analysis

Cross-species gene expression analysis was performed as previously described (Gafni et al., 2013) with some modifications. Human ESC array data was compared to previously published naive mouse ESC and primed mouse EpiSC gene expression datasets on an Agilent 4 X 44k array platform (GSE15603). Probeset mapping between the Agilent 4 X 44k array platform and Affymetric Primeview platform of human-mouse homologous genes was downloaded from Ensembl biomart (www.ensembl.org/biomart). The probesets of the same gene in human or mouse were next collapsed into a single value to represent the gene independently in each species by taking the mean value. The relative expression values from mouse and human were next calculated independently by dividing the expression values of the samples by the mean of expression values within the same genes across samples in the same species. Pair-wise comparisons were performed on the relative expression values from the human and mouse expression profiles using Pearson correlation coefficients (PCCs). The average linkage hierarchical clustering of the Pearson correlation was shown in the heatmap.

Chromatin Immunoprecipitation (ChIP)

Cells were cross-linked for 10 minutes at room temperature by the addition of one-tenth of the volume of 11% formaldehyde solution (11% formaldehyde, 50mM HEPES pH 7.3, 100 mM NaCl, 1 mM EDTA pH 8.0, 0.5 mM EGTA pH 8.0) to the growth media followed by quenching with 100 mM glycine. Cells were washed twice with PBS, then the supernatant was aspirated and the cell pellet was flash frozen in liquid nitrogen. Frozen cross-linked cells were stored at -80C. 20 ul of Dynal magnetic beads (Sigma) were blocked with 0.5% BSA (w/v) in PBS. Magnetic beads were bound with 2 ug of the indicated antibody. The antibodies used were as follows: H3K4me3 (Abcam ab8580) and H3K27me3 (Millipore 07-449). Cross-linked cells were lysed with lysis buffer 1 (50 mM HEPES pH 7.3, 140 mM NaCl, 1mM EDTA, 10% glycerol, 0.5% NP-40, and 0.25% Triton X-100) and resuspended and sonicated in sonication buffer (50 mM Tris-HCl [pH 7.5], 140 mM NaCl, 1 mM EDTA, 1% Triton X-100, 0.1% Na-deoxycholate, 0.1% SDS).

Cells were sonicated at 4°C with a Bioruptor (Diagenode) at high power for 25 cycles for 30s with 30s between cycles. Sonicated lysates were cleared and incubated overnight at 4°C with magnetic beads bound with antibody to enrich for DNA fragments bound by the indicated factor. Beads were washed two times with sonication buffer, one time with sonication buffer with 500 mM NaCl, one time with LiCl wash buffer (20 mM Tris pH 8.0, 1 mM EDTA, 250 mM LiCl, 0.5% NP-40, 0.5% Na-deoxycholate) and one time with TE with 50 mM NaCl. DNA was eluted in elution buffer (50 mM Tris-HCL pH 8.0, 10 mM EDTA, 1% SDS). Cross-links were reversed overnight. RNA and protein were digested using RNase A and Proteinase K, respectively and DNA was purified with phenol chloroform extraction and ethanol precipitation.

Illumina Sequencing and Library Generation

Purified ChIP DNA was used to prepare Illumina multiplexed sequencing libraries. Libraries for Illumina sequencing were prepared following the Illumina TruSeq DNA Sample Preparation v2 kit protocol with the following exceptions. After end-repair and A-tailing, Immunoprecipitated DNA (10-50 ng) or Whole Cell Extract DNA (50 ng) was ligated to a 1:50 dilution of Illumina Adaptor Oligo Mix assigning one of 24 unique indexes in the kit to each sample. Following ligation, libraries were amplified by 18 cycles of PCR using the HiFi NGS Library Amplification kit from KAPA Biosystems. Amplified libraries were then size-selected using a 2% gel cassette in the Pippin Prep system from Sage Science set to capture fragments between 200 and 400 bp. Libraries were quantified by qPCR using the KAPA Biosystems Illumina Library Quantification kit according to kit protocols. Libraries with distinct TruSeq indexes were multiplexed by mixing at equimolar ratios and running together in a lane on the Illumina HiSeq 2000 for 40 bases in single read mode.

Gene Sets and Annotations

All analyses were performed using RefSeq (NCBI37/HG19) (Pruitt et al., 2007) human gene annotations.

ChIP-Seq Data Processing

All ChIP-Seq datasets were aligned using Bowtie (version 0.12.9) (Langmead et al., 2009) to build version NCBI37/HG19 of the human genome using –n2, -e70, -m2, -k2, --best. We used the MACS version 1.4.1 (Model based analysis of ChIP-Seq) (Zhang et al., 2008) peak finding algorithm to identify regions of ChIP-Seq enrichment over background. A p-value threshold of enrichment of 1e-9 was used for all datasets.

Heatmap representation of read density profiles.

A gene was defined as Polycomb-associated if an enriched region for H3K27me3 (representing polycomb complexes) was located within +/- 1 kb of the TSS. H3K27me3 is a histone modification associated with Polycomb complexes (Boyer et al., 2006). The annotated TSS of Polycomb-associated genes were aligned at the center in the composite view of signal density profile. The average ChIP-seq read density (r.p.m./bp) around 5 kb centered on the centers in 50 bp bin was calculated.

Meta Representations of ChIP-Seq Occupancy

Genome-wide average ‘‘meta’’ representations of ChIP-seq occupancy of different factors were created by mapping ChIP-seq read density to Polycomb-associated genes. We created three sets of regions: upstream, gene body and downstream. 80 equally-sized bins divided the **-**2000 to 0 promoter region, 200 equally-sized bins divided the length of the gene body, and 80 equally-sized bins divided the 0 to +2kb downstream region. The average ChIP-Seq factor density in each bin was calculated to create a meta genome-wide average in units of rpm/bp.

Differentiation assays

Teratoma formation

Single cell dissociations of naive human ESCs were resuspended in 250 µl of medium and co-injected subcutaneously with 250 ul of matrigel in the flank of NOD/SCID mice. Tumors generally developed within 8 to 12 weeks and animals were sacrificed before tumor size exceeded 3 cm in diameter. Teratomas were isolated after sacrificing the mice and fixed in formalin. After sectioning, teratomas were diagnosed based on

hematoxylin and eosin staining. All animal experiments were performed in compliance with protocol # 1031-088-16 from the Committee on Animal Care at MIT.

Directed differentiation into hepatocytes

Differentiation of naive human ESCs into hepatocytes was obtained as described previously in conventional human ESCs (Si-Tayeb et al., 2010). Single cells were cultivated on Matrigel coated plates (2 mg/ml) under low oxygen conditions. Differentiation was initiated by cultivating cells for 5 days in Activin A (100 ng/ml) containing RPMI/B27 medium under ambient oxygen, followed by 5 days in BMP4 (20 ng/ml)/ FGF-2 (10 ng/ml) containing RPMI/B27 and 5 days in HGF (20 ng/ml) containing RPMI/B27 under 5% oxygen. Finally cells were cultured for 5 days in Hepatocyte Culture Medium supplemented with Oncostatin-M (20 ng/ml) under ambient oxygen conditions. Generated hepatocytes were identified by expression of AFP and HNF4a.

Morula and blastocyst injection

Six to eight weeks old B6D2F1 females were superovulated with 7.5 I.U of Pregnant Mare Serum (PMS) each given by intraperitoneal (IP) injections followed by an IP injection of 7.5 I.U of Human Chorionic Gonadotropin (HCG) 46 to 48 hours later. They were then mated with B6D2F1 stud males and checked for copulatory plugs the following day. One-cell fertilized embryos were harvested and incubated at 37°C, 5% O_2 for 2-3 days in KSOM medium (Zenith Biotech). At the 8-cell, morula, and blastocyst stage, embryos were injected with 10-15 naive human ESCs using a 16 μm piezo needle (Humagen). During injection, the human cells were kept continuously in drops of their own culture medium, whereas the embryos were kept in M2+ROCKi Y-27632 (Stemgent, 10 μM) drops. After injection, the injected embryos were cultured in KSOM+ROCKi for 3-4 hours, then washed in 5 individual drops of KSOM and cultured overnight to the blastocyst stage. When the injected embryos reached the blastocyst stage, 20 embryos were transferred into each E2.5 p.c pseudopregnant female by uterine transfer. Seven to eight days later, the post-op females were sacrificed by CO_2

asphyxiation and embryos were harvested at E9.5-E10.5 p.c for analysis. All animal experiments were performed in compliance with protocol # 1031-088-16 from the Committee on Animal Care at MIT. In addition, interspecies chimerism experiments were approved by the Embryonic Stem Cell Research Oversight *(*ESCRO*)* Committee at Whitehead Institute.

qRT-PCR

Total RNA was isolated using the Rneasy Kit (QIAGEN) and reversed transcribed using the Superscript III First Strand Synthesis kit (Invitrogen). Quantitative RT-PCR analysis was performed in triplicate using the ABI 7900 HT system with FAST SYBR Green Master Mix (Applied Biosystems). Gene expression was normalized to GAPDH. Error bars represent the standard deviation (SD) of the mean of triplicate reactions. Primer sequences are included in the table below.

Primers used in this study

**Gene**	**Primer sequence (5'- 3')**	**Application**
NANOG-F	GCAGAAGGCCTCAGCACCTA	RT-PCR
NANOG-R	AGGTTCCCAGTCGGGTTCA	
OCT4-F	GCTCGAGAAGGATGTGGTCC	RT-PCR
OCT4-R	CGTTGTGCATAGTCGCTGCT	
SOX2-F	CACTGCCCCTCTCACACATG	RT-PCR
SOX2-R	TCCCATTTCCCTCGTTTTTCT	
STELLA-F	GTTACTGGGCGGAGTTCGTA	RT-PCR
STELLA-R	TGAAGTGGCTTGGTGTCTTG	
KLF4-F	GATGGGGTCTGTGACTGGAT	RT-PCR
KLF4-R	CCCCCAACTCACGGATATAA	
GAPDH-F	CGAGATCCCTCCAAAATCAA	RT-PCR
GAPDH-R	ATCCACAGTCTTCTGGGTGG	

REX1-F	GGAATGTGGGAAAGCGTTCGT	RT-PCR
REX1-R	CCGTGTGGATGCGCACGT	
PRDM14-F	TGAGCCTTCAGGTCACAGAG	RT-PCR
PRDM14-R	ATTTCCTATCGCCCTTGTCC	
GFP-F	AGAACGGCATCAAGGTGAAC	RT-PCR
GFP-R	TGCTCAGGTAGTGGTTGTCG	
FUW-KLF2-F	GATTTTGCTGGGTTGGTTTTT	RT-PCR
FUW-KLF2-R	CCACATAGCGTAAAAGGAGCA	
FUW-NANOG-F	GCTGGGGAAGGCCTTAATGT	RT-PCR
FUW-NANOG-R	CCACATAGCGTAAAAGGAGCA	
PAX6-F	CTTTGCTTGGGAAATCCGAG	RT-PCR
PAX6-R	AGCCAGGTTGCGAAGAACTC	

II. Supplemental Figure and Table Legends

Figure S1. A reporter system for naive human pluripotency based on endogenous *OCT4* distal enhancer activity [associated with Figure 1]

(**A**) Southern blot analysis confirming deletion of the PE in *OCT4-2A-GFP* human ESCs and the removal of floxed PGK-puro cassette. NdeI-digested genomic DNA was hybridized with 5' and 3' external probes. Expected fragment size: WT (wild type) = 5.6kb, T (targeted) = 6.4 kb, ΔPE (targeted allele after PGK-puro removal) = 4.6kb. Note that some clones obtained after Cre excision exhibited deletion of the entire *OCT4-2A-GFP* sequence (*OCT4* KO allele) due to the presence of an additional loxP site from prior round of *OCT4-2A-GFP* targeting (Hockemeyer et al., 2011).

(**B**) Phase and GFP images of OCT4-ΔPE-GFP+ cells obtained after DOX induction of lentiviral KLF2+NANOG. Following primary infection, WIBR3 human ESCs containing the *OCT4-ΔPE-GFP* reporter allele were trypsinized and treated with hESM, 2i/L or 2i/L/DOX for one week. 40X magnification.

(**C**) Immunofluorescence staining for NANOG in a clonal line of OCT4-ΔPE-GFP-positive cells derived in 2i/L/DOX, and a clonal line of OCT4-ΔPE-GFP-negative primitive neural stem cells (pNSCs) derived in 2i/L. 100X magnification.

(**D**) Quantitative gene expression analysis for GFP, SOX2, PRMD14 and PAX6 in clonal OCT4-ΔPE-GFP+ human ESC lines generated in 2i/L/DOX, secondary primed cells generated by withdrawal of DOX and expansion in conventional hESM, and clonal lines of OCT4-ΔPE-GFP-negative pNSCs derived in 2i/L. Error bars indicate ±1 SD of technical replicates.

Figure S2. Identification of small molecules that maintain OCT4-ΔPE-GFP activity after transgene withdrawal [associated with Figure 2]

(**A**) Raw data obtained from high-throughput flow cytometric analysis of the proportion of OCT4-ΔPE-GFP+ cells in 96 wells supplemented with a kinase inhibitor library (n=2).

(**B**) Quantitative gene expression analysis for STELLA, KLF4, PRDM14 and SOX2 in a clonal line of OCT4-ΔPE-GFP+ cells maintained in 2i/L/DOX or for five passages without DOX in the presence of each candidate compound. Error bars indicate ±1 SD of technical replicates.

(**C**) Flow cytometric analyses of the proportion of viable (DAPI-negative) and OCT4-ΔPE-GFP+ cells in 2i/L/DOX or two passages after DOX withdrawal in 2i/L/SB590885 (1 μM).

Figure S3. Optimization of medium for maintaining viable OCT4-ΔPE-GFP+ cells [associated with Figure 3]

(**A**) Raw data obtained from high-throughput flow cytometric analysis of the proportion of DAPI-/OCT4-ΔPE-GFP+ cells in 96 wells supplemented with three plates of a kinase inhibitor library in the presence of the primary hit compound SB590885 (1 μM) (n=2). Asterisks denote autofluorescent compounds.

(**B**) Hit compounds from viability screen using a clonal line of WIBR3 OCT4-ΔPE-GFP+ ESCs established in 2i/L/DOX. Asterisks denote autofluorescent compounds.

(**C**) Phase images and flow cytometric analyses of OCT4-ΔPE-GFP+ cells maintained in 2i/L/SB590885 (0.5 μM) ± ROCK inhibitor Y-27632 (10 μM) for two passages. CHIR99021 was applied at the optimized concentration of 0.3 μM. 40X magnification.

(**D**) Phase and GFP images of OCT4-ΔPE-GFP+ cells maintained in 2i/L/DOX, 2i/L/SB590885 (0.5 μM) + ROCK inhibitor Y-27632 (10 μM) or the same medium in which CHIR99021 (0.3 μM) was replaced with an alternative GSK3 inhibitor, IM-12 (1.0 μM). 40X magnification.

Figure S4. Direct conversion of conventional human ESCs to naive pluripotency in 5i/L [associated with Figure 4]

(**A**) Karyotype analysis of naive human ESCs WIBR2 (P8 in 5i/L/A) indicates a normal diploid chromosome content. Cytogenetic analysis was performed on 20 metaphase cells.

(**B**) Karyotype analysis of naive human ESCs WIN1 (P7 in 5i/L/FA) indicates a normal diploid chromosome content. Cytogenetic analysis was performed on 20 metaphase cells.

(**C**) (Top) Strategy for generating naive human iPSCs from normal and patient-derived disease-specific primary fibroblasts through primary infection of OCT4, SOX2, KLF4 and MYC. (Bottom) Phase images of clonal naive iPSC lines reprogrammed from control and patient fibroblasts. 40X magnification.

(**D**) Phase images of OCT4-ΔPE-GFP+ cells three passages after withdrawal of individual inhibitors and growth factors. 40X magnification.

(**E**) Quantitative gene expression analysis for OCT4, SOX2, KLF2 and REX1 three passages after withdrawal of individual inhibitors and growth factors. Error bars indicate ±1 SD of technical replicates.

Figure S5. Evaluation of alternative culture conditions for naive human pluripotency. [associated with Figure 5]

(**A**) Phase and fluorescence images (Top) and flow cytometric analysis of the proportion of OCT4-ΔPE-GFP+ cells (Bottom) in OCT4-ΔPE-GFP+ cells derived in 5i/L/FA and maintained for three passages in the presence of additional media additives, as indicated. Where indicated recombinant human insulin (Sigma) was added at 12.5 μg/mL final concentration. 40X magnification.

(**B**) Quantitative gene expression analysis for KLF2 and KLF4 in OCT4-ΔPE-GFP-positive naive human ESCs cultured in 5i/L/A and supplemented with various components of the medium of Gafni et al. (2013). Error bars indicate ±1 SD of technical replicates.

(**C**) Phase and fluorescence images (Top) and flow cytometric analysis of the proportion of OCT4-ΔPE-GFP+ cells (Bottom) in OCT4-ΔPE-GFP+ cells derived in 5i/L/FA and maintained for three passages in 20% KSR basal medium supplemented with the media additives described in (A). 40X magnification.

Figure S6. Transcriptional profiling of naive human ESCs in 5i/L/A and 6i/L/A [associated with Figure 6]

(**A**) Gene ontology (GO) analysis showing up- and down-regulated gene categories with most significant p values between our naive human conditions and primed human ESCs.

(B) Quantitative gene expression analysis for KLF4 and REX1 in human ESCs cultured in parallel in primed medium, 5i/L/FA and the medium of Gafni et al. (2013). Error bars indicate ±1 SD of technical replicates.

(**C**) Expression profile of transcripts upregulated in 6i/L/A during human embryonic development. For each gene, the normalized expression values in human ESCs cultured in 6i/L/A vs. primed human ESCs are indicated (Left). An unpaired two-tailed t test was performed to establish the degree of significance. Expression of the corresponding transcript is shown at nine stages of human pre-implantation development, as detected by single cell RNA-Seq

profiling (Yan et al., 2013) (Right). This comparison indicates that naive-associated transcripts upregulated in 6i/L/A are enriched at the morula/epiblast stage of human development when compared to human ESCs at passage 0 or passage 10.

Figure S7. Additional characterization of naive human ESCs in 5i/L/A [associated with Figure 6]

(A) Single molecule (sm) RNA FISH analysis using OCT4 and NANOG probes in WIBR2 human ESCs cultured in primed medium, the medium of Gafni et al. (2013) or 5i/L/A (P5). Scale bar indicates 5 μm.

(B) Variability in NANOG expression compared between single human ESCs cultured in primed medium, 5i/L/A or the medium of Gafni et al. (2013).

(**C**) Teratomas generated from WIBR3 OCT4-ΔPE-GFP-positive human ESCs derived and maintained in 5i/L±FA and WIBR3 AAVS1-tdTomato human ESCs in 5i/L/FA. Representative tissues of the three germ layers are indicated. 200X magnification.

(**D**) Immunofluorescence staining for AFP and HNF4a following 20d of hepatic differentiation in naive WIBR2 human ESCs derived and maintained in 5i/L/A. Scale bars indicate 100 μm.

(**E**) Table summarizing injections of human ESCs maintained in 5i/L/FA (Top) or the medium of Gafni et al. (2013) (Bottom) into mouse embryos. C1-AAVS1-GFP human ESCs in the medium of Gafni et al. (2013) were cultured on MEFs, gelatin/vitronectin or matrigel prior to injection. (*) E10.5 embryos injected with human ESCs cultured in 5i/L/FA and the medium of Gafni et al. (2013) were mixed during collection, but none were identified as positive. (**) 30 injected embryos were lost during transfer.

Table S1. Gene expression of naive and primed human ESCs [associated with Figure 6]

Normalized gene expression values from microarray samples shown in Figure 6A. Dataset includes two primed human ESC samples (hESM) and five naive human ESC samples (5i/L/A or 6i/L/A). Genes are ordered according to Column I (log_2FC naive vs. primed) with the most upregulated genes in the naive state at the top of the list.

III. Supplemental Figures

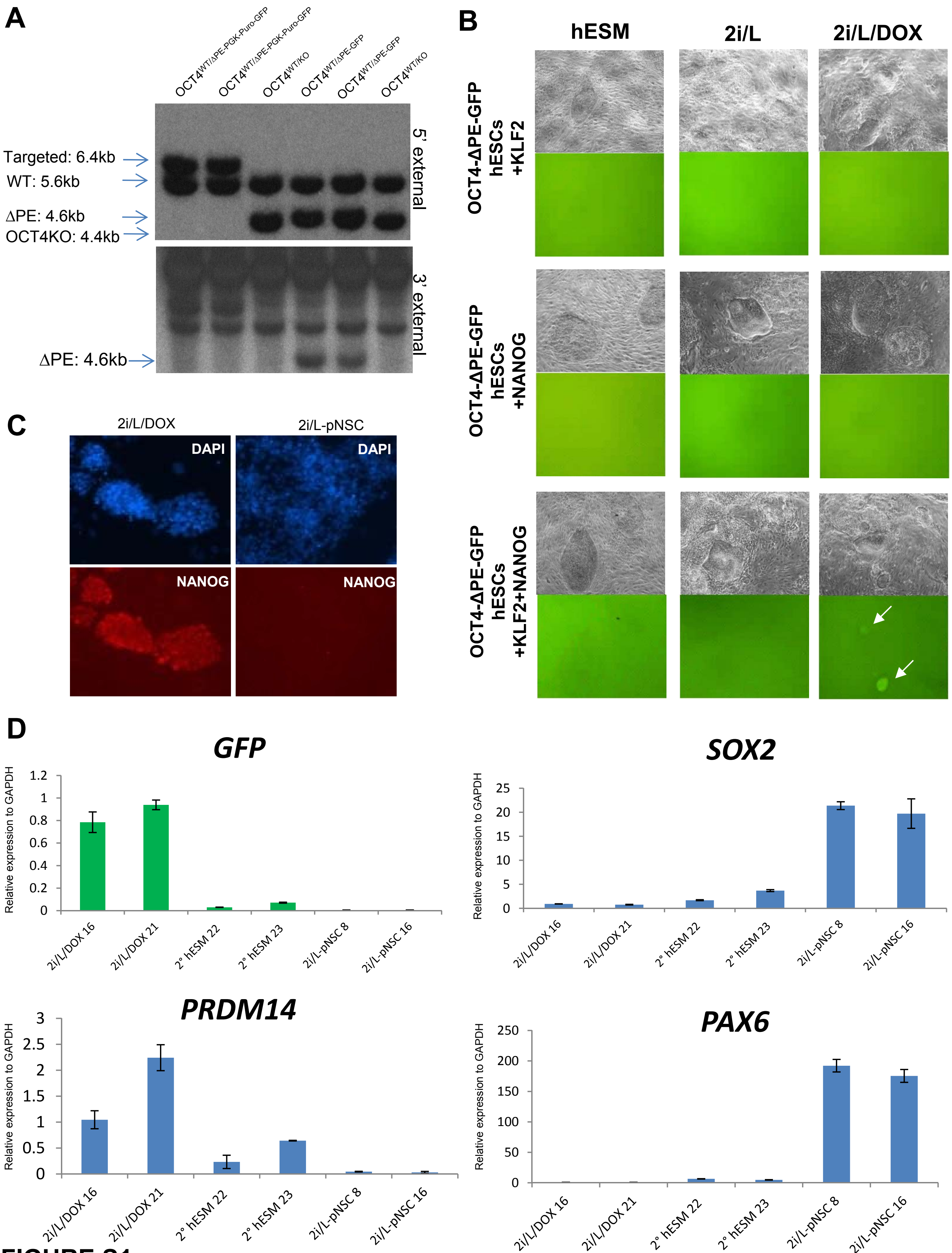
A
OCT4WT/ΔPE-PGK-Puro-GFP
OCT4WT/ΔPE-PGK-Puro-GFP
OCT4WT/KO
OCT4WT/ΔPE-GFP
OCT4WT/ΔPE-GFP
OCT4WT/KO
Targeted: 6.4kb
WT: 5.6kb
ΔPE: 4.6kb
OCT4KO: 4.4kb
5' external
3' external
ΔPE: 4.6kb
B
hESM
2i/L
2i/L/DOX
OCT4-ΔPE-GFP hESCs +KLF2
OCT4-ΔPE-GFP hESCs +NANOG
OCT4-ΔPE-GFP hESCs +KLF2+NANOG
C
2i/L/DOX
2i/L-pNSC
DAPI
DAPI
NANOG
NANOG
D
GFP
SOX2
PRDM14
PAX6
Relative expression to GAPDH
2i/L/DOX 16
2i/L/DOX 21
2° hESM 22
2° hESM 23
2i/L-pNSC 8
2i/L-pNSC 16

FIGURE S1

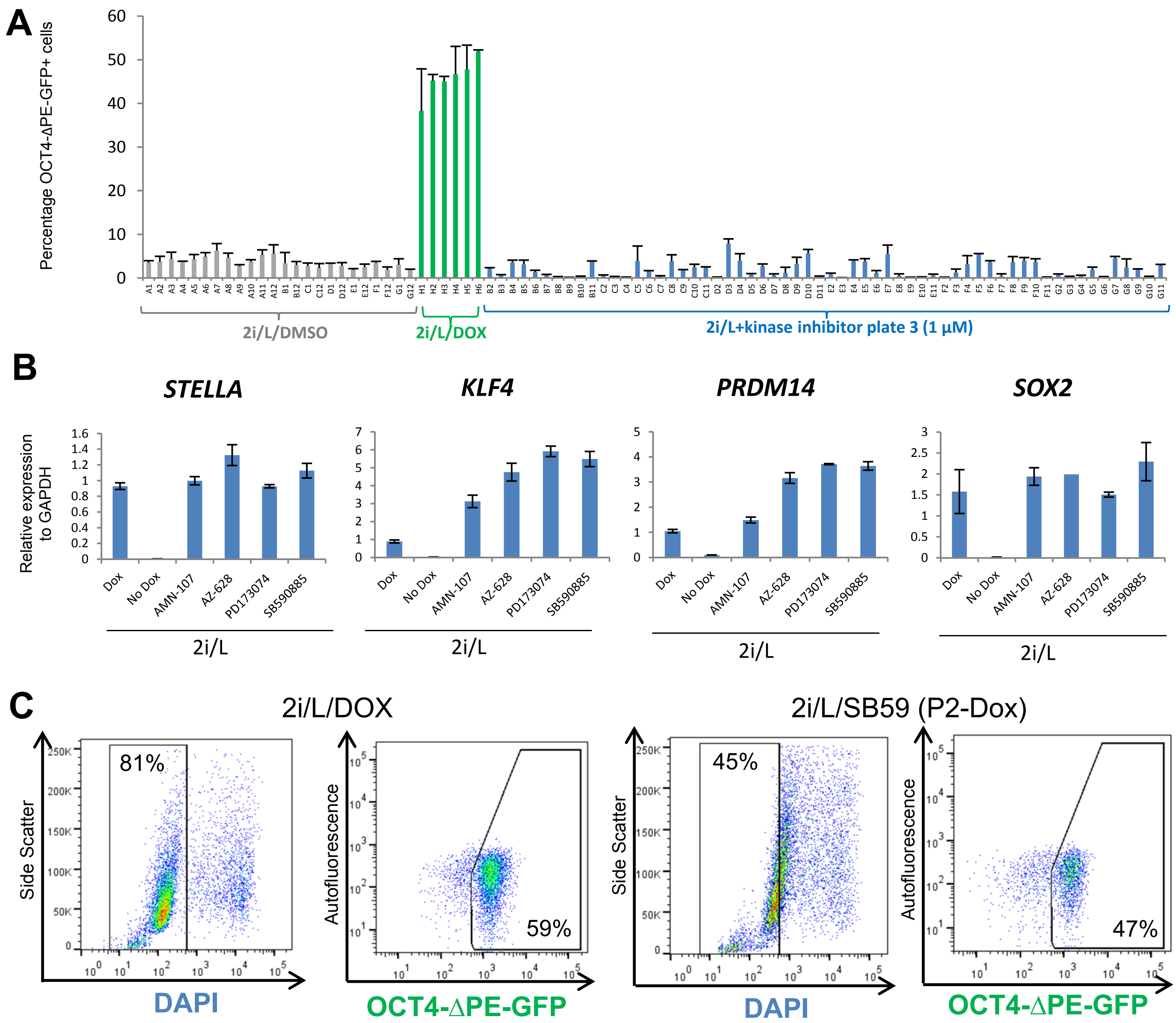

A
Percentage OCT4-ΔPE-GFP+ cells
2i/L/DMSO
2i/L/DOX
2i/L+kinase inhibitor plate 3 (1 μM)
B
STELLA
KLF4
PRDM14
SOX2
Relative expression to GAPDH
Dox
No Dox
AMN-107
AZ-628
PD173074
SB590885
2i/L
C
2i/L/DOX
2i/L/SB59 (P2-Dox)
Side Scatter
Autofluorescence
81%
59%
45%
47%
DAPI
OCT4-ΔPE-GFP

FIGURE S2

A

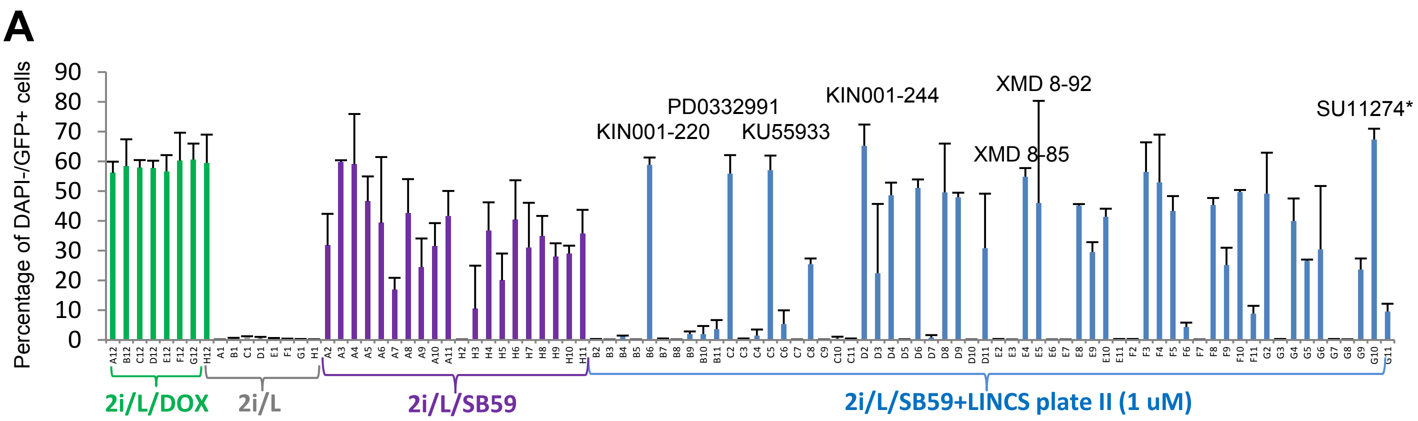

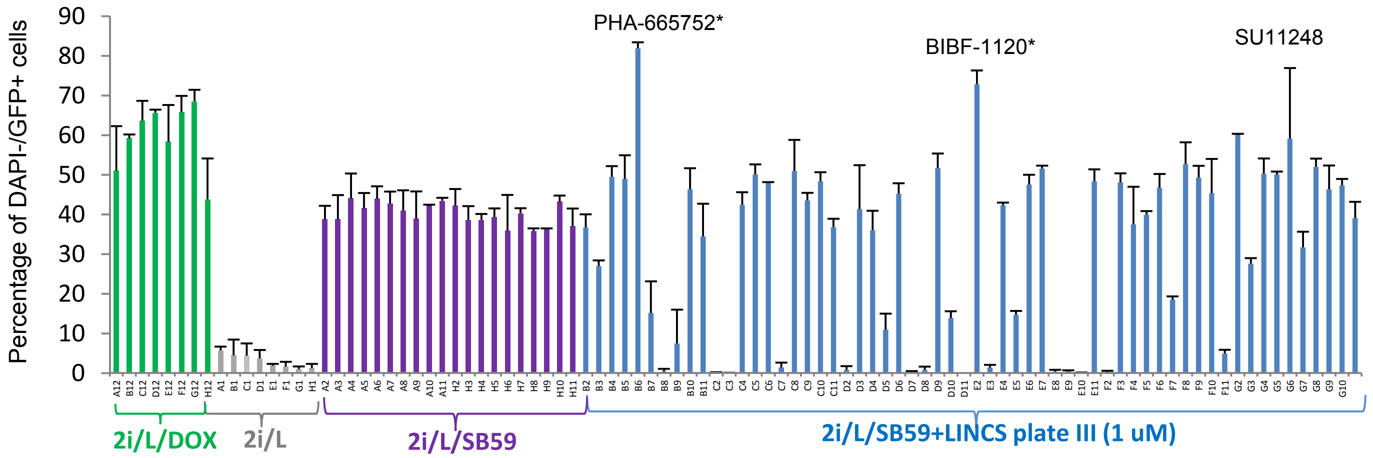

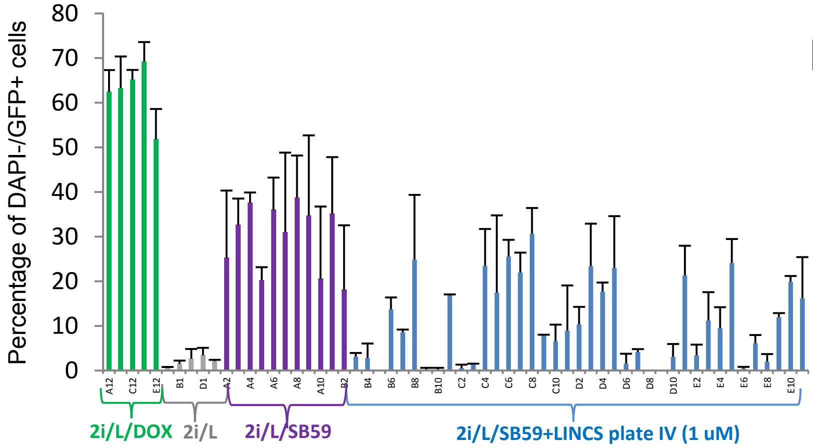

B

Compound	Target(s)
WH-4-023	SRC
KIN001-220	Aurora kinase
PD0332991	CDK4
KU55933	ATM
KIN001-244	PDK
XMD 8-85	ERK5
XMD 8-92	ERK5
SU11274*	MET
PHA-665752*	MET
BIBF-1120*	VEGFR, PDGFR, FGFR
SU11248	VEGFR1, PDGFR, KIT, FLT3

C

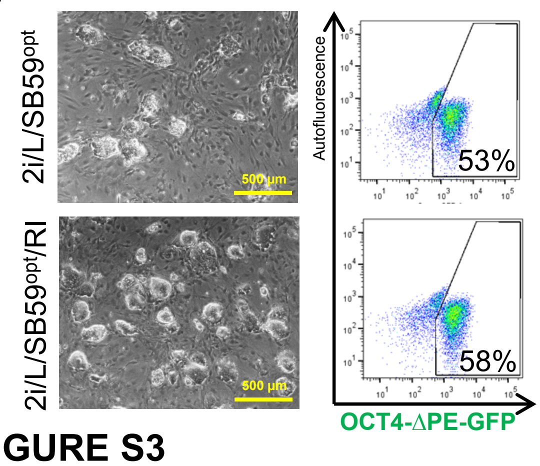

D

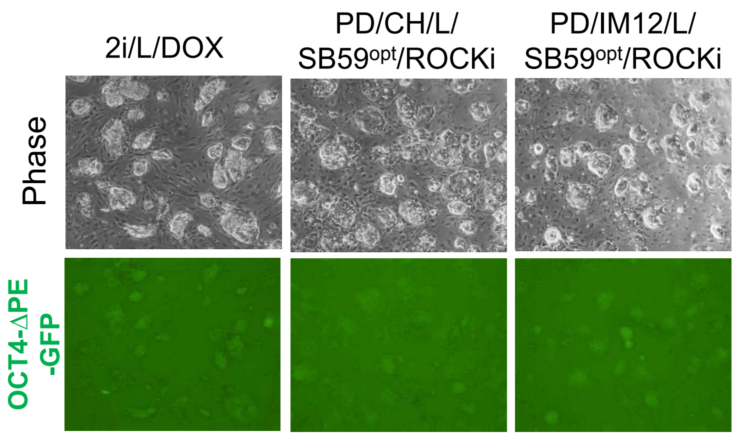

FIGURE S3

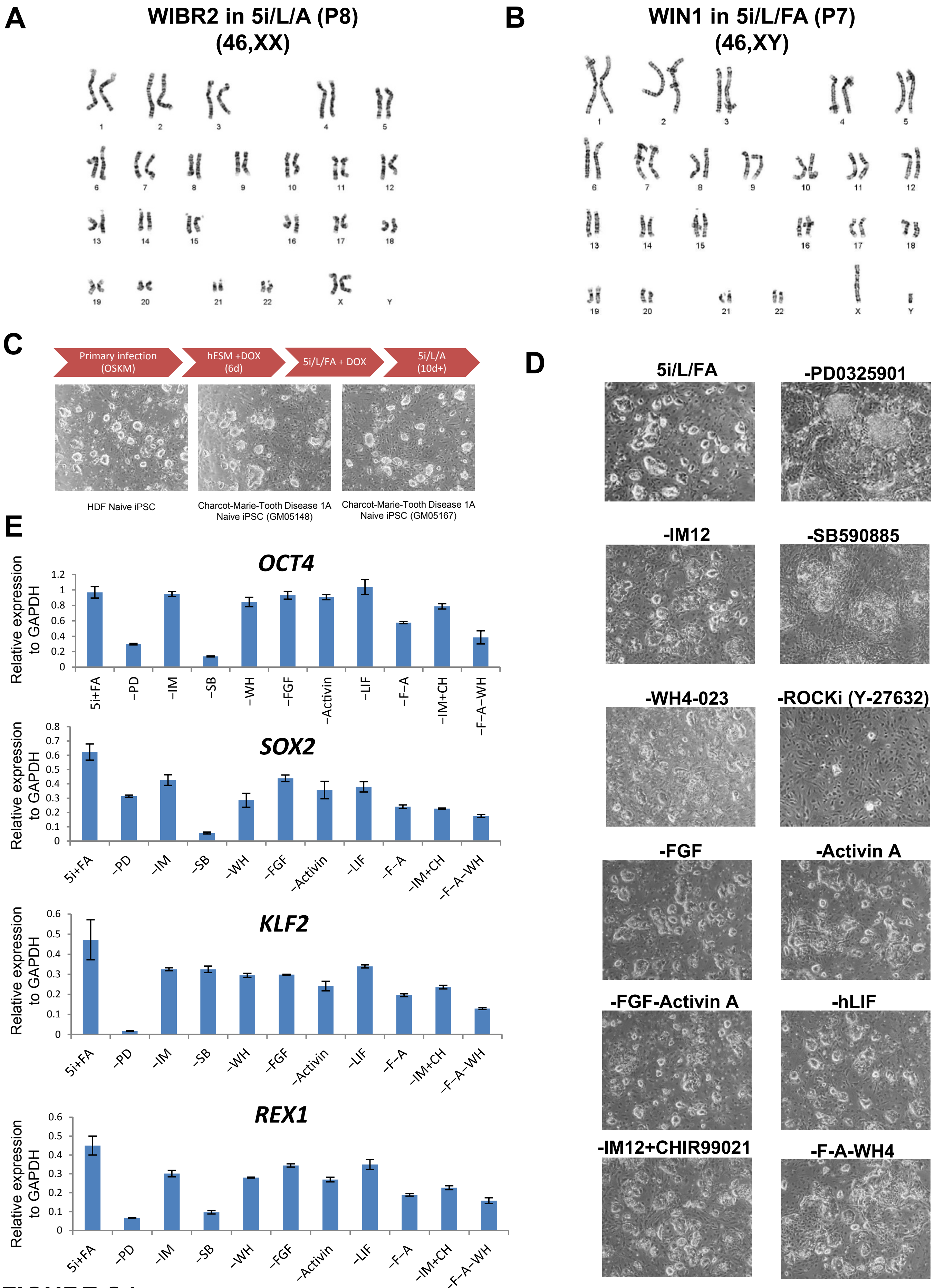
A
WIBR2 in 5i/L/A (P8)
(46,XX)
1 2 3 4 5
6 7 8 9 10 11 12
13 14 15 16 17 18
19 20 21 22 X Y
B
WIN1 in 5i/L/FA (P7)
(46,XY)
1 2 3 4 5
6 7 8 9 10 11 12
13 14 15 16 17 18
19 20 21 22 X Y
C
Primary infection (OSKM)
hESM +DOX (6d)
5i/L/FA + DOX
5i/L/A (10d+)
HDF Naive iPSC
Charcot-Marie-Tooth Disease 1A Naive iPSC (GM05148)
Charcot-Marie-Tooth Disease 1A Naive iPSC (GM05167)
D
5i/L/FA
-PD0325901
-IM12
-SB590885
-WH4-023
-ROCKi (Y-27632)
-FGF
-Activin A
-FGF-Activin A
-hLIF
-IM12+CHIR99021
-F-A-WH4
E
OCT4
Relative expression to GAPDH
0 0.2 0.4 0.6 0.8 1 1.2
5i+FA –PD –IM –SB –WH –FGF –Activin –LIF –F–A –IM+CH –F–A–WH
SOX2
Relative expression to GAPDH
0 0.1 0.2 0.3 0.4 0.5 0.6 0.7 0.8
5i+FA –PD –IM –SB –WH –FGF –Activin –LIF –F–A –IM+CH –F–A–WH
KLF2
Relative expression to GAPDH
0 0.1 0.2 0.3 0.4 0.5 0.6
5i+FA –PD –IM –SB –WH –FGF –Activin –LIF –F–A –IM+CH –F–A–WH
REX1
Relative expression to GAPDH
0 0.1 0.2 0.3 0.4 0.5 0.6
5i+FA –PD –IM –SB –WH –FGF –Activin –LIF –F–A –IM+CH –F–A–WH

FIGURE S4

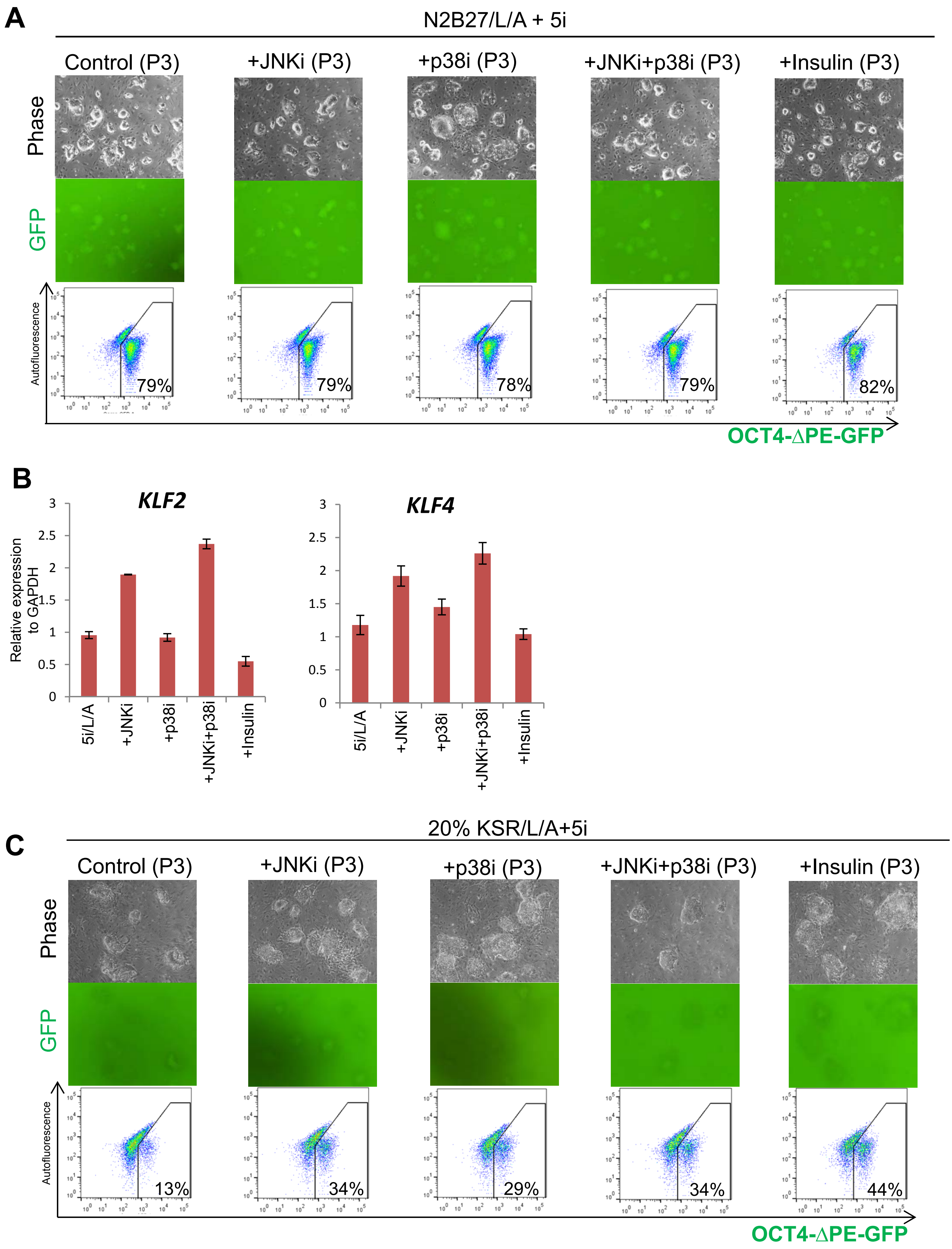
A
N2B27/L/A + 5i
Control (P3)
+JNKi (P3)
+p38i (P3)
+JNKi+p38i (P3)
+Insulin (P3)
Phase
GFP
Autofluorescence
79%
79%
78%
79%
82%
OCT4-ΔPE-GFP
B
KLF2
KLF4
Relative expression to GAPDH
3
2.5
2
1.5
1
0.5
0
5i/L/A
+JNKi
+p38i
+JNKi+p38i
+Insulin
C
20% KSR/L/A+5i
Control (P3)
+JNKi (P3)
+p38i (P3)
+JNKi+p38i (P3)
+Insulin (P3)
Phase
GFP
Autofluorescence
13%
34%
29%
34%
44%
OCT4-ΔPE-GFP

FIGURE S5

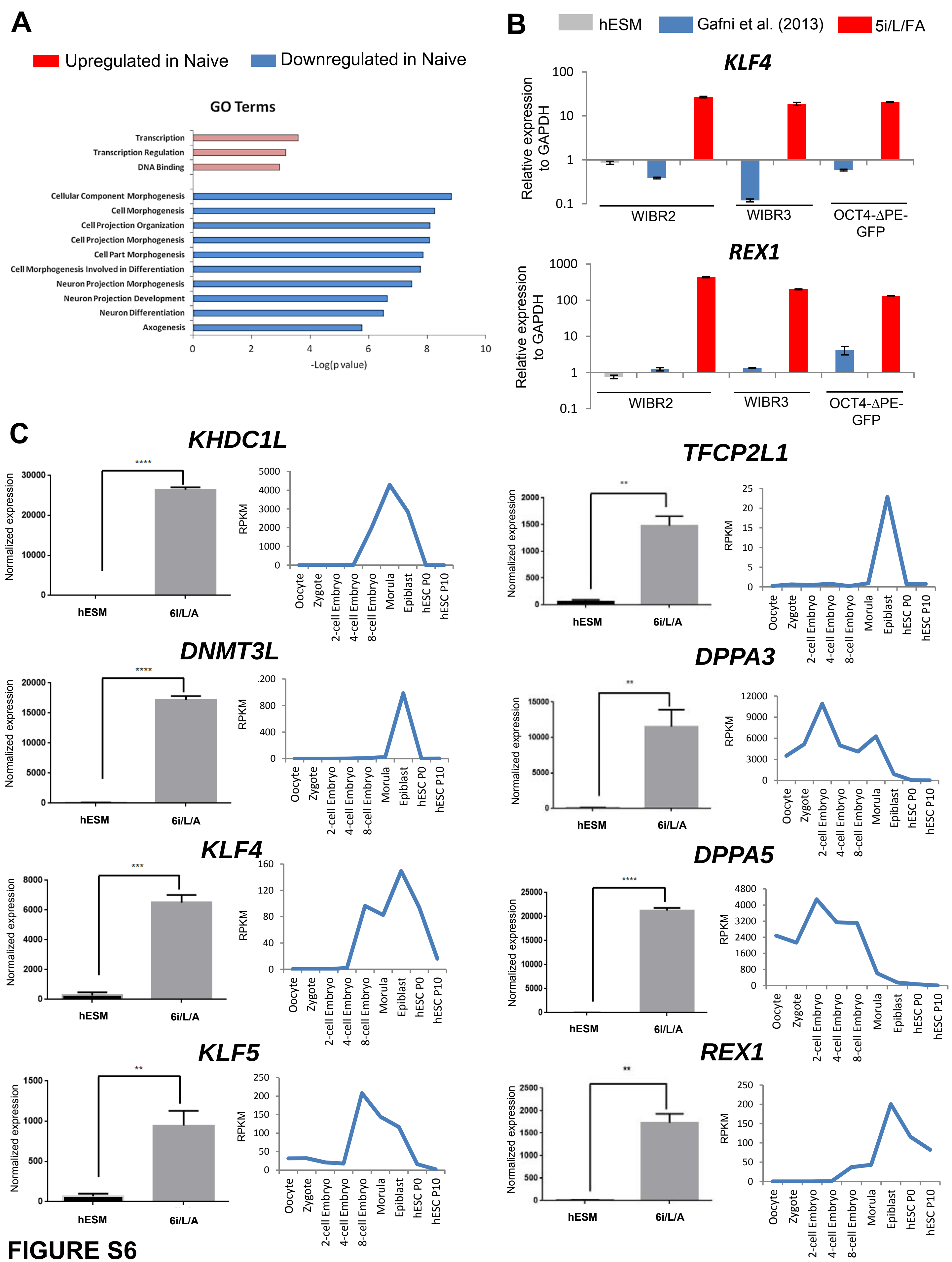
A
Upregulated in Naive
Downregulated in Naive
GO Terms
Transcription
Transcription Regulation
DNA Binding
Cellular Component Morphogenesis
Cell Morphogenesis
Cell Projection Organization
Cell Projection Morphogenesis
Cell Part Morphogenesis
Cell Morphogenesis Involved in Differentiation
Neuron Projection Morphogenesis
Neuron Projection Development
Neuron Differentiation
Axogenesis
-Log(p value)
B
hESM
Gafni et al. (2013)
5i/L/FA
KLF4
Relative expression to GAPDH
WIBR2
WIBR3
OCT4-ΔPE-GFP
REX1
C
KHDC1L
DNMT3L
KLF4
KLF5
TFCP2L1
DPPA3
DPPA5
REX1
Normalized expression
RPKM
hESM
6i/L/A
Oocyte
Zygote
2-cell Embryo
4-cell Embryo
8-cell Embryo
Morula
Epiblast
hESC P0
hESC P10

FIGURE S6

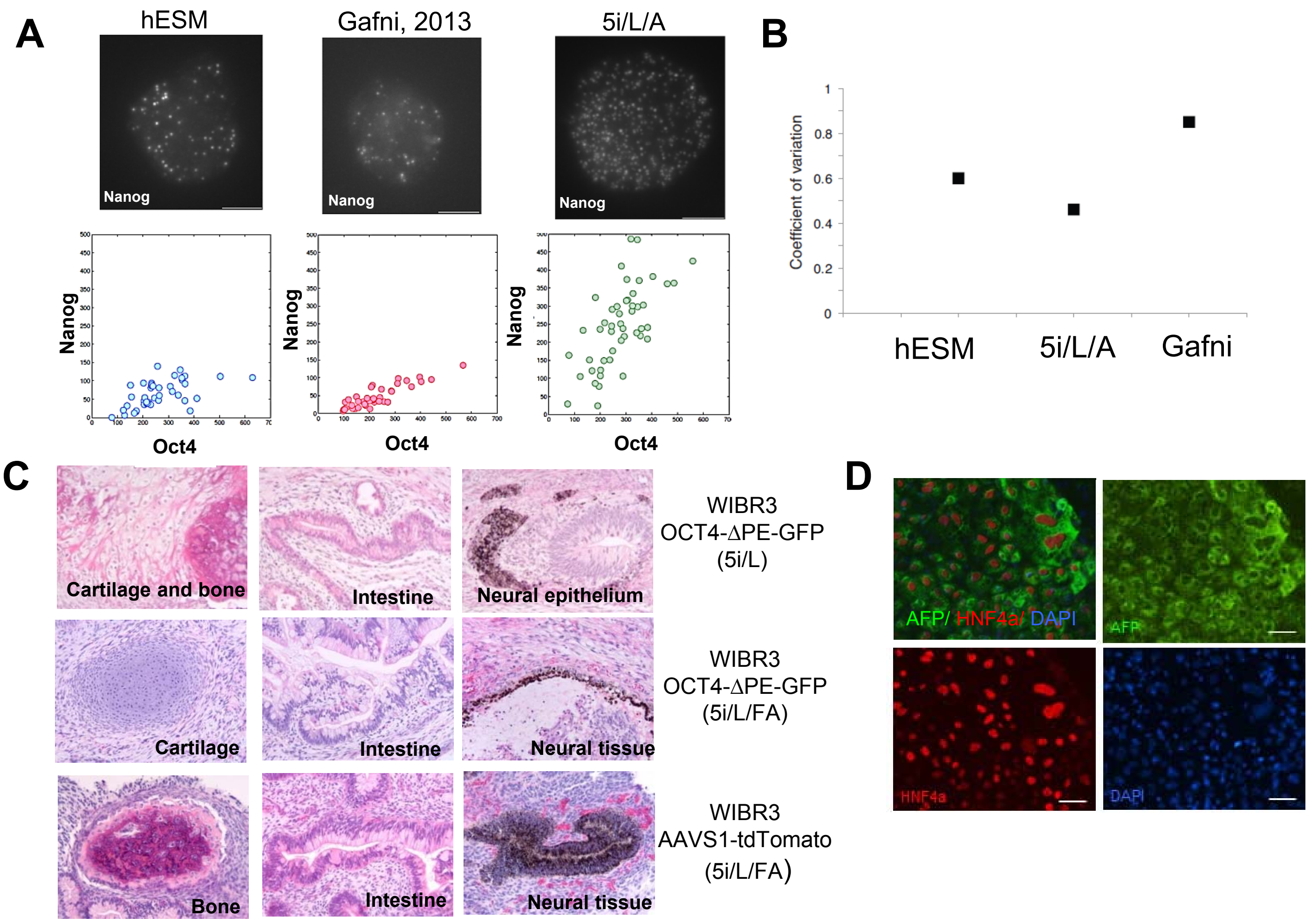

E

5i/L/FA medium Human-Mouse injections							
**Dev. Stage**	8-cell	8-cell	Morula	Morula	Blastocyst	Blastocyst	Total
**Cell line**	WIBR3 AAVS1-td Tomato	WIBR3 AAVS1-GFP	WIBR3 AAVS1-tdTomato	WIBR3 AAVS1-GFP	WIBR3 AAVS1-tdTomato	WIBR3 AAVS1-GFP	-
**Injected embryos**	245	363	92	60	50	50	860
**Embryos recovered at E10.5**	92	164*	47	28	11	26	368
**Positive embryos**	0	0	0	0	0	0	0
**Gafni et al. (2013) medium Human-Mouse injections**							
**Dev. stage**	8-cell	8-cell	8-cell	Morula	Morula	Blastocyst	Total
**Cell line**	C1 AAVS1-GFP on MEFs	C1 AAVS1-GFP on gel/vit	C1 AAVS1-GFP on matrigel	C1 AAVS1-GFP on MEFs	C1 AAVS1-GFP on gel/vit	C1 AAVS1-GFP on matrigel	-
**Injected embryos**	140	100	40	40	40	76	436
**Embryos recovered at E10.5**	96*	28	12	24	7**	28	195
**Positive embryos**	0	0	0	0	0	0	0

FIGURE S7

IV. Supplemental References

Boyer, L.A., Plath, K., Zeitlinger, J., Brambrink, T., Medeiros, L.A., Lee, T.I., Levine, S.S., Wernig, M., Tajonar, A., Ray, M.K., *et al.* (2006). Polycomb complexes repress developmental regulators in murine embryonic stem cells. Nature *441*, 349-353.

Brambrink, T., Foreman, R., Welstead, G.G., Lengner, C.J., Wernig, M., Suh, H., and Jaenisch, R. (2008). Sequential expression of pluripotency markers during direct reprogramming of mouse somatic cells. Cell stem cell *2*, 151-159.

Bruck, T., and Benvenisty, N. (2011). Meta-analysis of the heterogeneity of X chromosome inactivation in human pluripotent stem cells. Stem cell research *6*, 187-193.

Chan, Y.S., Goke, J., Ng, J.H., Lu, X., Gonzales, K.A., Tan, C.P., Tng, W.Q., Hong, Z.Z., Lim, Y.S., and Ng, H.H. (2013). Induction of a human pluripotent state with distinct regulatory circuitry that resembles preimplantation epiblast. Cell stem cell *13*, 663-675.

Costa, M., Dottori, M., Sourris, K., Jamshidi, P., Hatzistavrou, T., Davis, R., Azzola, L., Jackson, S., Lim, S., Pera, M., *et al.* (2007). A method for genetic modification of human embryonic stem cells using electroporation. Nat Protoc *2*, 792-796.

Faddah, D.A., Wang, H., Cheng, A.W., Katz, Y., Buganim, Y., and Jaenisch, R. (2013). Single-cell analysis reveals that expression of nanog is biallelic and equally variable as that of other pluripotency factors in mouse ESCs. Cell stem cell *13*, 23-29.

Gafni, O., Weinberger, L., Mansour, A.A., Manor, Y.S., Chomsky, E., Ben-Yosef, D., Kalma, Y., Viukov, S., Maza, I., Zviran, A., *et al.* (2013). Derivation of novel human ground state naive pluripotent stem cells. Nature *504*, 282-286.

Hockemeyer, D., Wang, H., Kiani, S., Lai, C.S., Gao, Q., Cassady, J.P., Cost, G.J., Zhang, L., Santiago, Y., Miller, J.C., *et al.* (2011). Genetic engineering of human pluripotent cells using TALE nucleases. Nature biotechnology *29*, 731-734.

Itzkovitz, S., Lyubimova, A., Blat, I.C., Maynard, M., van Es, J., Lees, J., Jacks, T., Clevers, H., and van Oudenaarden, A. (2011). Single-molecule transcript counting of stem-cell markers in the mouse intestine. Nat Cell Biol *14*, 106-114.

Langmead, B., Trapnell, C., Pop, M., and Salzberg, S.L. (2009). Ultrafast and memory-efficient alignment of short DNA sequences to the human genome. Genome biology *10*, R25.

Pruitt, K.D., Tatusova, T., and Maglott, D.R. (2007). NCBI reference sequences (RefSeq): a curated non-redundant sequence database of genomes, transcripts and proteins. Nucleic acids research *35*, D61-65.

Raj, A., Rifkin, S.A., Andersen, E., and van Oudenaarden, A. (2010). Variability in gene expression underlies incomplete penetrance. Nature *463*, 913-918.

Raj, A., van den Bogaard, P., Rifkin, S.A., van Oudenaarden, A., and Tyagi, S. (2008). Imaging individual mRNA molecules using multiple singly labeled probes. Nat Methods *5*, 877-879.

Si-Tayeb, K., Noto, F.K., Nagaoka, M., Li, J., Battle, M.A., Duris, C., North, P.E., Dalton, S., and Duncan, S.A. (2010). Highly efficient generation of human hepatocyte-like cells from induced pluripotent stem cells. Hepatology *51*, 297-305.

Smyth, G.K. (2004). Linear models and empirical bayes methods for assessing differential expression in microarray experiments. Statistical applications in genetics and molecular biology *3*, Article3.

Soldner, F., Hockemeyer, D., Beard, C., Gao, Q., Bell, G.W., Cook, E.G., Hargus, G., Blak, A., Cooper, O., Mitalipova, M.*, et al.* (2009). Parkinson's disease patient-derived induced pluripotent stem cells free of viral reprogramming factors. Cell *136*, 964-977.

Soldner, F., Laganiere, J., Cheng, A.W., Hockemeyer, D., Gao, Q., Alagappan, R., Khurana, V., Golbe, L.I., Myers, R.H., Lindquist, S.*, et al.* (2011). Generation of isogenic pluripotent stem cells differing exclusively at two early onset Parkinson point mutations. Cell *146*, 318-331.

Ware, C.B., Nelson, A.M., Mecham, B., Hesson, J., Zhou, W., Jonlin, E.C., Jimenez-Caliani, A.J., Deng, X., Cavanaugh, C., Cook, S.*, et al.* (2014). Derivation of naive human embryonic stem cells. Proc Natl Acad Sci U S A *111*, 4484-4489.

Xu, C., Jiang, J., Sottile, V., McWhir, J., Lebkowski, J., and Carpenter, M.K. (2004). Immortalized fibroblast-like cells derived from human embryonic stem cells support undifferentiated cell growth. Stem cells *22*, 972-980.

Yan, L., Yang, M., Guo, H., Yang, L., Wu, J., Li, R., Liu, P., Lian, Y., Zheng, X., Yan, J., *et al.* (2013). Single-cell RNA-Seq profiling of human preimplantation embryos and embryonic stem cells. Nature structural & molecular biology *20*, 1131-1139.

Zhang, Y., Liu, T., Meyer, C.A., Eeckhoute, J., Johnson, D.S., Bernstein, B.E., Nusbaum, C., Myers, R.M., Brown, M., Li, W., *et al.* (2008). Model-based analysis of ChIP-Seq (MACS). Genome biology *9*, R137.
