# Supplementary material for: Systematic Identification of Culture Conditions for Induction and Maintenance of Naive Human Pluripotency
Source: Cell Stem Cell. 2014 Oct 2;15(4):471–87. doi: 10.1016/j.stem.2014.07.002 (PMC4184977; doi:10.1016/j.stem.2014.07.002)
Supplement: Document S2. Article plus Supplemental Information [file mmc3.pdf]

Cell Stem Cell
Article

Systematic Identification of Culture Conditions for Induction and Maintenance of Naive Human Pluripotency

Thorold W. Theunissen,[1,5] Benjamin E. Powell,[1,5] Haoyi Wang,[1,5] Maya Mitalipova,[1] Dina A. Faddah,[1,2] Jessica Reddy,[1,2] Zi Peng Fan,[1,3] Dorothea Maetzel,[1] Kibibi Ganz,[1] Linyu Shi,[1] Tenzin Lungjangwa,[1] Sumeth Imsoonthornruksa,[1] Yonatan Stelzer,[1] Sudharshan Rangarajan,[1] Ana D'Alessio,[1] Jianming Zhang,[4] Qing Gao,[1] Meelad M. Dawlaty,[1] Richard A. Young,[1,2] Nathanael S. Gray,[4] and Rudolf Jaenisch[1,2,*]
[1]Whitehead Institute for Biomedical Research, Cambridge, MA 02142, USA
[2]Department of Biology, Massachusetts Institute of Technology, Cambridge, MA 02142, USA
[3]Computational and Systems Biology Program, Massachusetts Institute of Technology, Cambridge, MA 02139, USA
[4]Department of Cancer Biology, Dana-Farber Cancer Institute, Harvard Medical School, Boston, MA 02115, USA
[5]Co-first author
*Correspondence: jaenisch@wi.mit.edu
http://dx.doi.org/10.1016/j.stem.2014.07.002
This is an open access article under the CC BY license (http://creativecommons.org/licenses/by/3.0/).

SUMMARY

Embryonic stem cells (ESCs) of mice and humans have distinct molecular and biological characteristics, raising the question of whether an earlier, "naive" state of pluripotency may exist in humans. Here we took a systematic approach to identify small molecules that support self-renewal of naive human ESCs based on maintenance of endogenous *OCT4* distal enhancer activity, a molecular signature of ground state pluripotency. Iterative chemical screening identified a combination of five kinase inhibitors that induces and maintains *OCT4* distal enhancer activity when applied directly to conventional human ESCs. These inhibitors generate human pluripotent cells in which transcription factors associated with the ground state of pluripotency are highly upregulated and bivalent chromatin domains are depleted. Comparison with previously reported naive human ESCs indicates that our conditions capture a distinct pluripotent state in humans that closely resembles that of mouse ESCs. This study presents a framework for defining the culture requirements of naive human pluripotent cells.

INTRODUCTION

Human pluripotent stem cells, including embryonic stem cells (ESCs) and induced pluripotent stem cells (iPSCs), share molecular and functional properties with epiblast stem cells (EpiSCs) derived from the mouse postimplantation epiblast (Brons et al., 2007; Tesar et al., 2007). It has been suggested that these cells represent a primed state of pluripotency that is distinct from the naive pluripotent ground state of mouse ESCs and iPSCs (Nichols and Smith, 2009). EpiSCs can be converted to naive pluripotency by combined chemical and genetic manipulation (Guo et al., 2009; Hanna et al., 2009; Silva et al., 2009). A question of significant interest is whether human ESCs can be converted to the naive state. Initial studies reported that dual inhibition of MEK and GSK3 (2i), leukemia inhibitory factor (LIF), and overexpression of transcription factors associated with naive pluripotency can induce features of ground state pluripotency in human ESCs (Hanna et al., 2010; Wang et al., 2011).

Recently, several groups have described culture conditions for maintaining transgene-independent human ESCs that share various properties with mouse ESCs (Chan et al., 2013; Gafni et al., 2013; Valamehr et al., 2014; Ware et al., 2014). Of note, Hanna and colleagues tested combinations of 16 inhibitors and growth factors for maintenance of OCT4-GFP expression (Gafni et al., 2013). Because Oct4 is equally expressed between mouse ESCs and EpiSCs (Brons et al., 2007; Tesar et al., 2007), this marker does not distinguish a priori between naive and primed states. The most compelling evidence for acquisition of naive pluripotency in this study was the reported contribution of naive human ESCs to interspecies chimeras after their injection into mouse morulae (Gafni et al., 2013). Ng and colleagues screened a combination of 20 compounds for enhanced expression of NANOG in mTesr1, a customized medium for human ESCs containing high levels of FGF and TGFβ. This study reported that a combination of 2i, hLIF, and Dorsomorphin induced upregulation of a number of genes expressed in the human preimplantation embryo (Chan et al., 2013). In contrast with these two studies, two other recent papers reported that 2i and FGF are sufficient to maintain naive-like human ESCs in the presence (Valamehr et al., 2014) or absence (Ware et al., 2014) of hLIF.

Here we established a specific reporter system for naive human pluripotency using transcription activator-like effector nuclease (TALEN)-based genome editing, and we performed an iterative chemical screen to identify kinase inhibitors that induce and maintain activity of this reporter. These optimized conditions enable both the interconversion between conventional and naive human ESCs in the absence of reprogramming factors and the direct isolation of naive ESCs from human blastocysts. We also evaluate previously reported protocols for capturing naive human ESCs and observe substantial differences with our cells in terms of reporter activity, transcriptional

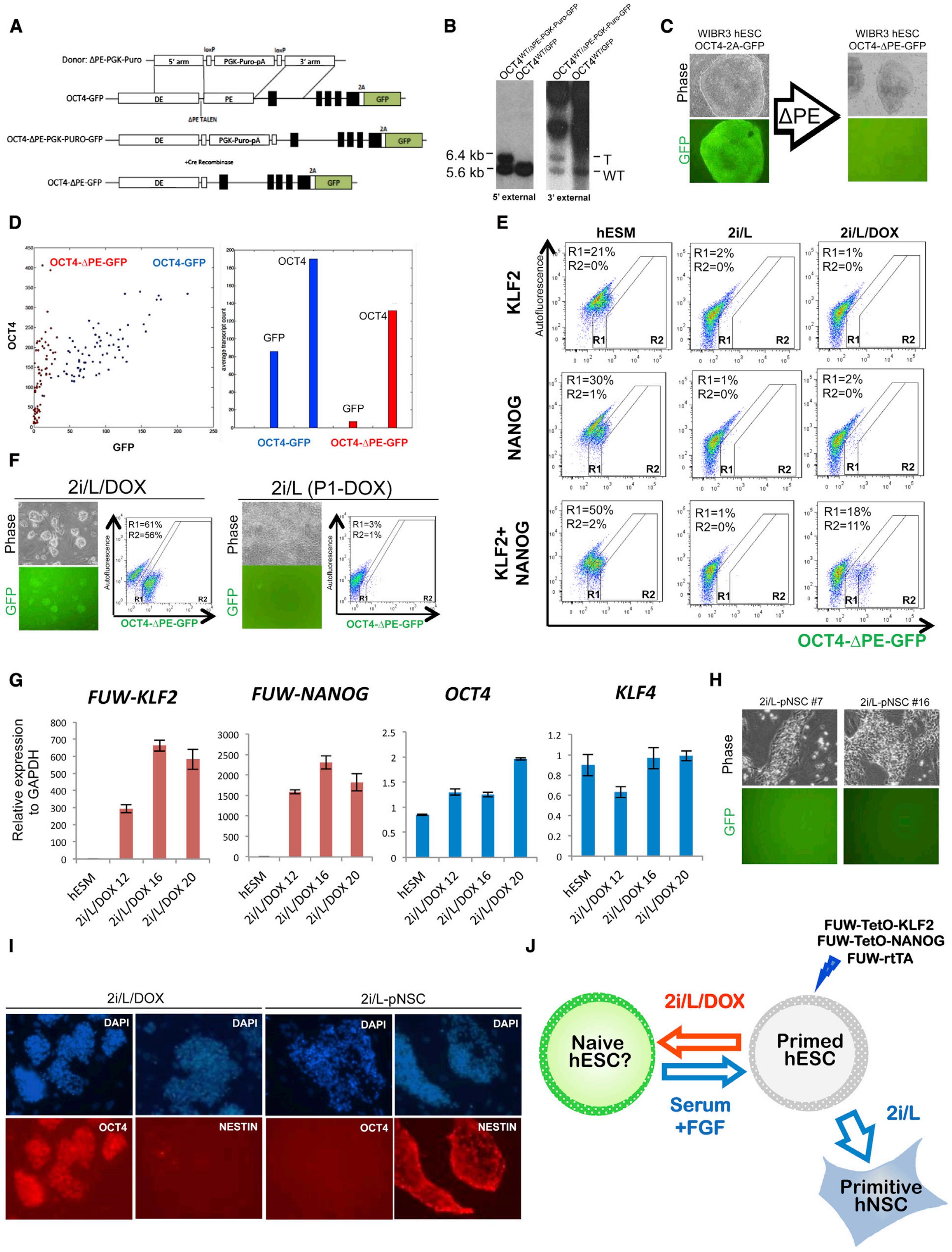

(legend on next page)

profile, and cellular homogeneity. Based on these findings we postulate that our combination of kinase inhibitors captures a distinct state of human pluripotency that shares defining features with mouse ESCs.

RESULTS

A Reporter System for Naive Human Pluripotency Based on *OCT4* Distal Enhancer Activity

An important molecular signature of naive pluripotency in the mouse system is the use of the distal enhancer (DE) of *OCT4*. This element controls *Oct4* expression in naive mouse ESCs, preimplantation mouse embryos, and germ cells (Yeom et al., 1996). In contrast, expression of *Oct4* in primed EpiSCs and in the mouse postimplantation embryo is under the control of the proximal enhancer (PE) element (Tesar et al., 2007). To detect rare naive human ESCs in a large population of primed cells, we engineered a reporter system for *OCT4* DE activity using TALENs. We deleted the PE element from an *OCT4-2A-GFP* allele (Hockemeyer et al., 2011) (Figure 1A and Figure S1A available online). TALENs were designed to cleave in the 5′ end of the PE, together with a donor vector containing LoxP sites bordering a selectable marker and gene sequences homologous to those flanking the PE. After being targeted, the allele harbors an approximately 1 kb deletion of the PE sequence. We confirmed successful integration of this PE targeting vector (Figure 1B) and subsequent removal of the selection cassette (Figure S1A). As expected, deletion of the PE resulted in substantial attenuation of the OCT4-2A-GFP signal in the resulting $OCT4^{WT/\Delta PE\text{-}GFP}$ (from here on referred to as *OCT4-ΔPE-GFP*) primed human ESCs (Figure 1C). Single-molecule (sm) RNA FISH analysis in individual cells showed that GFP expression was diminished while OCT4 expression was reduced approximately by 50% after PE removal (Figure 1D). These changes in OCT4 and GFP expression indicate that *OCT4* is predominantly transcribed from the wild-type allele containing an intact PE sequence rather than the *OCT4-ΔPE-GFP* allele. Hence, OCT4 expression in primed human ESCs is primarily dependent on the PE rather than the DE, as observed in mouse EpiSCs.

We investigated whether overexpression of transcription factors specific to naive pluripotency together with the application of serum-free 2i and hLIF (2i/L) conditions would result in augmented OCT4-ΔPE-GFP activity. We overexpressed the transcription factors KLF2 and NANOG using doxycycline (DOX)-inducible lentiviral expression vectors. Compared to its family members Klf4 and Klf5, Klf2 has an enhanced capability to induce naive pluripotency in mouse EpiSCs (Hall et al., 2009). In addition, the homeodomain transcription factor Nanog is critical for the establishment of naive pluripotency (Silva et al., 2009) and can revert EpiSCs to the naive state in the absence of kinase inhibition (Theunissen et al., 2011). Consistent with the potent effects of these factors in the mouse system, we found that combined overexpression of KLF2 and NANOG in primed human ESCs resulted in increased OCT4-ΔPE-GFP reporter levels in a fraction of cells (Figure 1E and Figure S1B). The appearance of cells with increased levels of reporter activity was dependent on the expression of both factors and could only be observed in the presence of 2i/L/DOX. GFP+ colonies were clonally expanded on a mouse embryonic fibroblast (MEF) feeder layer in the presence of 2i/L/DOX while retaining a dome-like colony morphology and pluripotency gene expression (Figures 1F and 1G). Strikingly, withdrawal of DOX-dependent KLF2 and NANOG expression resulted in the rapid loss of colony morphology, the appearance of differentiated cells, and the shutdown of OCT4-ΔPE-GFP reporter activity within 7 days (Figure 1F, right). Thus, 2i/L culture conditions are insufficient to maintain the self-renewal of OCT4-ΔPE-GFP+ cells after withdrawal of exogenous factor expression. We considered that the rapid downregulation of naive reporter activity provides a defined time window in which to screen for small molecules that support the maintenance of a putative naive human pluripotent state.

Our inability to maintain OCT4-ΔPE-GFP activity in 2i/L led us to further investigate the consequences of these culture conditions when applied directly to conventional human ESCs in the absence of transgene expression. In the mouse system dual MEK and GSK3 inhibition is thought to consolidate the ground state and eliminate EpiSCs and other differentiated phenotypes

Figure 1. A Reporter System for Naive Human Pluripotency Based on Endogenous *OCT4* Distal Enhancer Activity

(A) Proximal enhancer (PE) targeting strategy in human ESCs containing a 2A-GFP sequence in frame with the 3′ UTR of *OCT4*.

(B) Southern blot analysis confirming disruption of PE in OCT4-2A-GFP ESCs. NdeI-digested genomic DNA was hybridized with 5′ and 3′ external probes. Expected fragment size: WT (wild-type) = 5.6 kb, T (targeted) = 6.4 kb.

(C) Images of OCT4-2A-GFP human ESCs before (left) and after (right) TALEN-mediated deletion of the PE. 40× magnification.

(D) Single-molecule RNA FISH analysis for OCT4 and GFP transcripts in OCT4-2A-GFP human ESCs before and after TALEN-mediated disruption of the PE.

(E) Flow cytometric analysis of the proportion of OCT4-ΔPE-GFP+ cells obtained after DOX induction of lentiviral KLF2, NANOG, or KLF2+NANOG. After primary infection WIBR3 human ESCs containing the *OCT4-ΔPE-GFP* reporter allele were trypsinized and treated with primed human ESC medium (hESM), 2i/L, or 2i/L/DOX for 1 week. R1, total proportion of GFP+ cells, includes weak GFP activity observed in primed human ESCs; R2, subset of cells with high GFP activity observed only upon combined overexpression of KLF2+NANOG.

(F) Phase and fluorescence images and flow cytometric analysis of a clonal line of WIBR3 OCT4-ΔPE-GFP+ cells derived in 2i/L/DOX (left). Phase and fluorescence images and flow cytometric analysis after replating in the absence of DOX for 1 week (right) are also shown. 40× magnification.

(G) Quantitative gene expression analysis for lentiviral FUW-KLF2, lentiviral FUW-NANOG, endogenous OCT4, and endogenous KLF4 in WIBR3 hESCs cultured in hESM and clonal OCT4-ΔPE-GFP+ derivatives generated in 2i/L/DOX. Error bars indicate ± 1 SD of technical replicates.

(H) Phase and fluorescence images of primitive neural stem cells (pNSCs) derived by treating WIBR3 hESCs containing the *OCT4-ΔPE-GFP* allele with 2i/L for three passages. 100× magnification.

(I) Immunofluorescence staining for OCT4 and NESTIN in a clonal line of OCT4-ΔPE-GFP+ cells derived in 2i/L/DOX and a clonal line of OCT4-ΔPE-GFP− pNSCs derived in 2i/L. 100× magnification.

(J) Model representing the distinct phenotypic responses of hESCs to treatment with 2i/L and 2i/L/DOX. OCT4-ΔPE-GFP+ cells generated in 2i/L/DOX do not maintain reporter activity upon transgene withdrawal. OCT4-ΔPE-GFP+ cells can revert back to the conventional “primed” hESC state by re-exposure to serum and FGF.

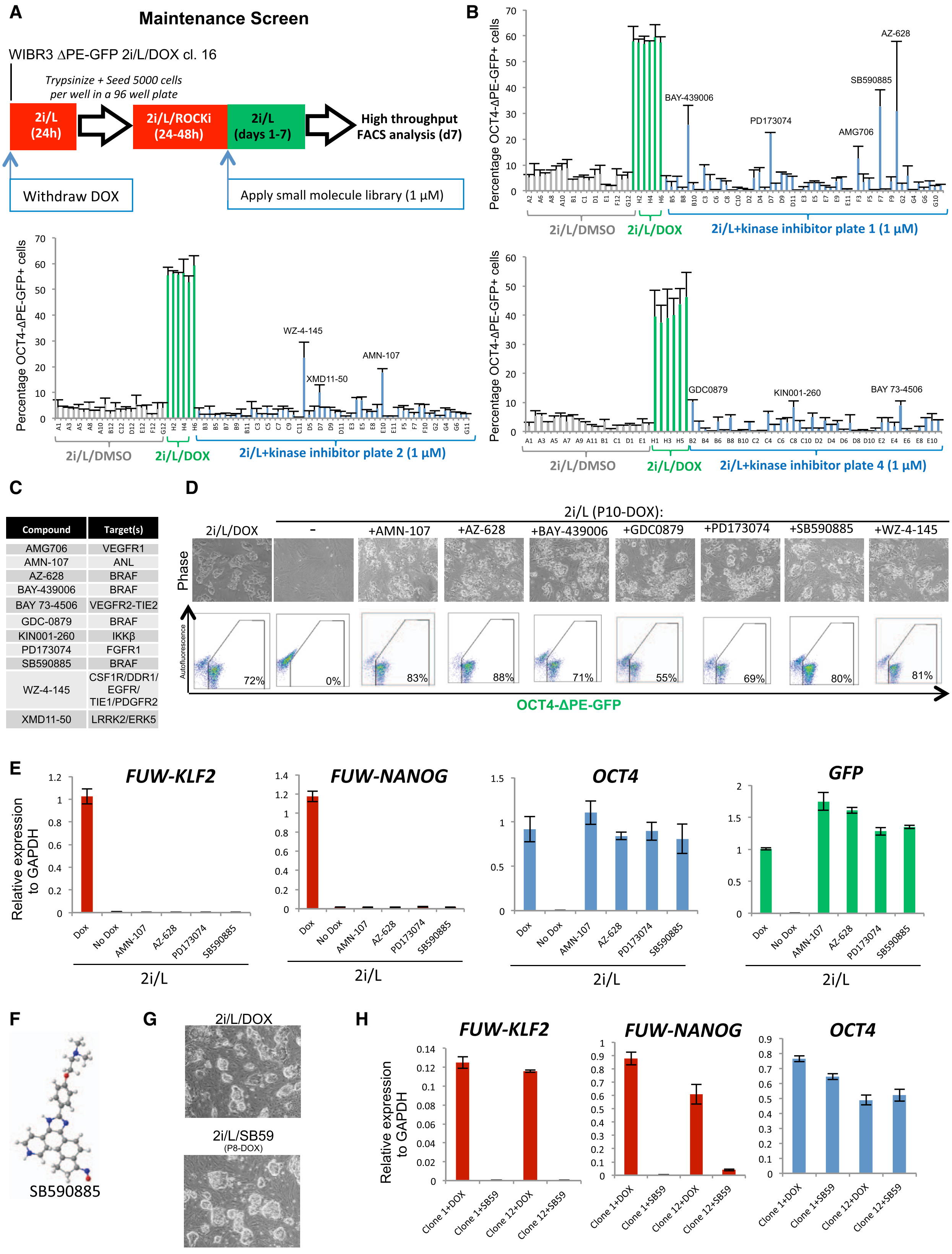

Compound	Target(s)
AMG706	VEGFR1
AMN-107	ANL
AZ-628	BRAF
BAY-439006	BRAF
BAY 73-4506	VEGFR2-TIE2
GDC-0879	BRAF
KIN001-260	IKKβ
PD173074	FGFR1
SB590885	BRAF
WZ-4-145	CSF1R/DDR1/ EGFR/ TIE1/PDGFR2
XMD11-50	LRRK2/ERK5

(legend on next page)

that cannot survive under these minimal conditions (Silva and Smith, 2008). In contrast, we observed rapid expansion in serum-free 2i/L of initially dome-shaped human ESC colonies that assumed a neural morphology upon further passaging (Figure 1H). Flow cytometric analysis revealed that weak levels of OCT4-ΔPE-GFP detected in primed human ESCs completely disappeared in 2i/L alone (Figure 1E). Consistent with the morphological change, we observed the loss of OCT4 and NANOG expression and upregulation of NESTIN and PAX6 expression in primed human ESCs expanded in serum-free 2i/L medium (Figure 1I and Figures S1C and S1D). These observations are in agreement with a report that 2i/L treatment induces differentiation of human ESCs into primitive neural stem cells (Hirano et al., 2012). We conclude that 2i/L conditions do not interfere with expansion of differentiated cell types in human cells to the same extent as in the mouse system and are consequently insufficient for stabilizing naive human pluripotency (Figure 1J).

Identification of Compounds that Maintain Naive Reporter Activity upon Transgene Withdrawal

To identify compounds that sustain OCT4-ΔPE-GFP activity in the absence of KLF2 and NANOG expression, we screened a kinase inhibitor library in the presence of 2i/L for a period of 7 days after DOX withdrawal in 96-well format (Figure 2A). This screen identified 10 different hit compounds that partially rescued the proportion of GFP+ cells when assayed by high-throughput FACS analysis (Figures 2B–2C and Figure S2A). Notable among these hits were four separate inhibitors of BRAF and four inhibitors of upstream receptor tyrosine kinases (RTKs) including DDR1, VEGFR1/2, and FGFR1. We then performed a validation experiment in 6-well format by withdrawing DOX and culturing the cells in 2i/L supplemented with each hit compound for up to 10 passages (Figure 2D). Nearly all hit compounds maintained a proportion of GFP+ cells similar to that observed with DOX. However, we noticed that the BRAF inhibitor SB590885 preserved the best colony morphology and proliferation. In contrast, treatment with the FGF receptor inhibitor PD173074 or pan-RTK inhibitor WZ-4-145 resulted in maintenance of OCT4-ΔPE-GFP activity in colonies with a disorganized morphology. Gene expression analysis five passages after DOX withdrawal confirmed the absence of KLF2 and NANOG transgene expression and the retention of endogenous OCT4 and GFP transcripts (Figure 2E and Figure S2B). To validate these findings in an independent system, we introduced DOX-inducible KLF2 and NANOG transgenes in a line of wild-type WIBR3 human ESCs. Upon receiving application of DOX and 2i/L, dome-shaped colonies appeared that could be expanded clonally. After DOX withdrawal these lines were maintained in 2i/L/SB590885 while retaining both colony morphology and the expression of endogenous *OCT4* (Figures 2F–2H). Thus, high-throughput chemical screening identified a number of kinase inhibitors, most notably the BRAF inhibitor SB590885, that synergize with 2i/L to maintain OCT4-ΔPE-GFP reporter activity and pluripotency gene expression in human ESCs after removal of exogenous KLF2 and NANOG expression.

Using a FACS-based live/dead discrimination assay, we determined that cells maintained in 2i/L/SB590885 had reduced viability (Figure S2C). This led us to consider whether other small molecules could cooperate with 2i/L/SB590885 to improve the fraction of viable GFP+ cells. Inclusion of a live/dead assay enhanced the resolution of our 96-well high-throughput FACS analysis, allowing more sensitive discrimination between the proportion of viable OCT4-ΔPE-GFP+ cells cultured in 2i/L/DOX and that of those cultured in 2i/L/SB590885 (Figure 3A). We included this viability assay in a modified screen in which GFP+ cells were cultured for two passages after DOX withdrawal in 2i/L/SB590885 supplemented with the kinase inhibitor library (Figures 3B–3C and Figure S3A). This screen identified several hit compounds that improved the fraction of viable GFP+ cells, including the LCK/SRC inhibitor WH-4-023 (Figure S3B). We also performed a titration of the concentrations of PD0325901, CHIR99021, and SB590885 during two passages after DOX withdrawal followed by high-throughput FACS analysis (Figure 3D). We observed a significant improvement in the proportion of viable GFP+ cells using a concentration of 1 μM PD0325901, 0.3 μM CHIR99021, and 0.5 μM SB590885. Notably, lower concentrations of CHIR99021 improved the proportion of viable GFP+ cells, whereas higher concentrations of CHIR99021 had an opposite effect. This is reminiscent of recent evidence that lowered GSK3 inhibition reduces differentiation and enhances the self-renewal of rat ESCs (Meek et al., 2013).

The proportion of viable OCT4-ΔPE-GFP+ cells after DOX withdrawal was further improved by a combination of optimized concentrations of PD0325901, CHIR99021, and SB590885 with 1 μM WH-4-023 (Figure 3F). We also included the ROCK inhibitor Y-27632 (Figure S3C). Finally, we observed improved morphology upon long-term culture without DOX by replacing

Figure 2. Identification of Small Molecules that Maintain OCT4-ΔPE-GFP Activity after Transgene Withdrawal

(A) Strategy for screening a kinase inhibitor library to identify compounds that maintain OCT4-ΔPE-GFP reporter activity upon withdrawal of DOX-dependent KLF2 and NANOG expression.

(B) Raw data obtained from high-throughput flow cytometric analysis of the proportion of OCT4-ΔPE-GFP+ cells in 96-well plates supplemented with a kinase inhibitor library (n = 2).

(C) Hit compounds from maintenance screen using a clonal line of WIBR3 OCT4-ΔPE-GFP+ ESCs established in 2i/L/DOX.

(D) Phase images (top) and flow cytometric analysis of the proportion of OCT4-ΔPE-GFP+ cells (bottom) in a clonal line of OCT4-ΔPE-GFP+ cells derived in 2i/L/DOX and maintained for 10 passages without DOX in the presence of each candidate compound. 40× magnification.

(E) Quantitative gene expression analysis for lentiviral FUW-KLF2, lentiviral FUW-NANOG, endogenous OCT4, and GFP in a clonal line of OCT4-ΔPE-GFP+ cells maintained in 2i/L/DOX or for five passages without DOX in the presence of each candidate compound. Error bars indicate ± 1 SD of technical replicates.

(F) Chemical structure of the BRAF inhibitor SB590885.

(G) Phase images of a clonal line of WIBR3 human ESCs established in 2i/L upon DOX-mediated induction of KLF2 and NANOG (top), and the same line maintained for eight passages without DOX in 2i/L/SB590885 (1 μM) (bottom). 40× magnification.

(H) Quantitative gene expression analysis for lentiviral FUW-KLF2, lentiviral FUW-NANOG, and endogenous OCT4 in two clonal lines of WIBR3 human ESCs maintained for eight passages without DOX in 2i/L/SB590885 (1 μM). Error bars indicate ± 1 SD of technical replicates.

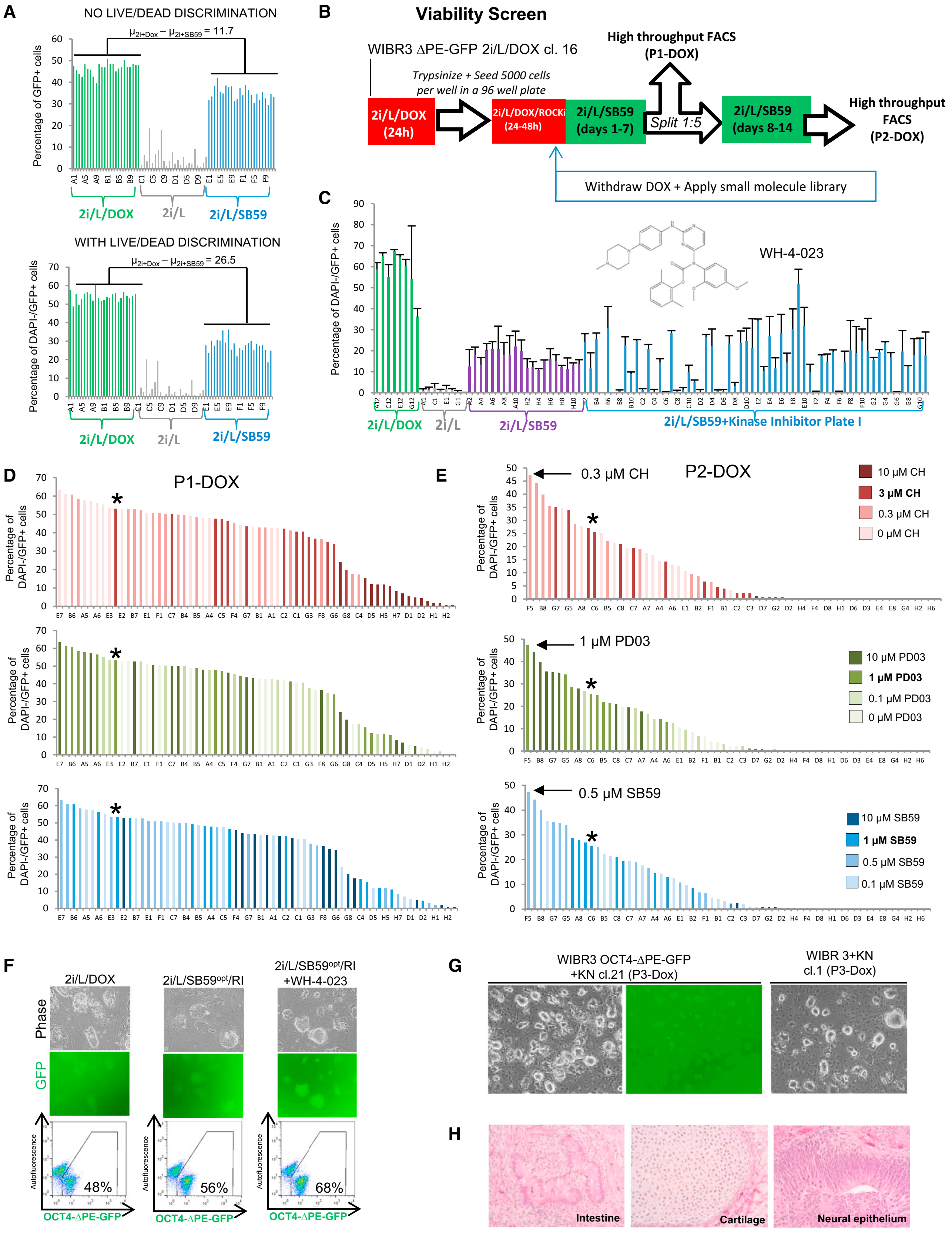

(legend on next page)

CHIR99021 with an alternative GSK3 inhibitor, IM12 (Figure 3G and Figure S3D). Pluripotency of putative naive human ESCs maintained under these conditions was confirmed by the generation of high-grade teratomas, including tissues representing all three germ layers, after their subcutaneous injection into NOD/SCID mice (Figure 3H). In summary, we have identified a combination of five compounds, including inhibitors of MEK, GSK3, BRAF, ROCK, and SRC, which supports the expansion of viable OCT4-ΔPE-GFP+ human pluripotent cells after exogenous transcription factor expression has been removed.

Generation of Transgene-free Naive Human ESCs in 5i/L

We then investigated the consequences of applying our optimized 5i/L medium to conventional human ESCs in the absence of ectopic factor expression. Not surprisingly, this highly selective inhibitor cocktail generated widespread cell death within 2 days of treatment. However, we observed the emergence of a small number of dome-shaped colonies within 10 days that were positive for the OCT4-ΔPE-GFP reporter (Figure 4B). These colonies were isolated and clonally expanded after dissociation in trypsin (Figure 4B). The appearance of these OCT4-ΔPE-GFP+ cells in our optimized chemical conditions suggested that overexpression of KLF2 and NANOG may be dispensable for driving primed human ESCs to the naive state. However, the slow and inefficient kinetics of this chemical conversion event led us to consider whether providing additional growth factor support might boost the efficiency of naive cell induction. Provision of 5i/L supplemented with FGF and Activin A (5i/L/FA) enhanced the kinetics of OCT4-ΔPE-GFP induction (Figure 4C) and enabled conversion of wild-type WIBR2 primed human ESCs into a cell state with identical morphology (Figure 4D) and normal karyotype at passage 8 (Figure S4A). Given the selective nature of the 5i culture regimen, we speculate that additional growth factor support prolongs the time window during which primed human ESCs are amenable to convert to the naive state. We then considered whether these culture conditions would support the direct derivation of ESC lines from human blastocysts. Application of 5i/L/FA to human blastocyst outgrowths resulted in the establishment of human ESCs with a similar dome-shaped colony morphology (Figure 4E) and normal karyotype at passage 7 (Figure S4B). Furthermore, application of 5i/L/FA after activation of OSK(M) in secondary C1 OCT4-ΔPE-GFP fibroblasts or primary patient-derived fibroblasts enabled the isolation of naive human iPSCs (Figure 4F and Figure S4C). Hence, 5i/L/FA promotes induction of OCT4-ΔPE-GFP activity in the absence of reprogramming transgenes and the derivation of putative naive human pluripotent cells from blastocysts or fibroblasts.

Genome stability is a subject of considerable interest in human pluripotent stem cell culture. It has been reported that single-cell dissociation using trypsin can accelerate the acquisition of chromosomal abnormalities (Chan et al., 2008). We have observed an abnormal karyotype in several converted and embryo-derived human ESC lines in 5i/L/FA. This suggests that naive human ESCs may be susceptible to acquiring chromosomal abnormalities, similar to previous observations with naive rat ESCs (Li et al., 2008). Further work is needed to assess the long-term karyotypic stability of naive human ESCs.

To investigate the role of individual components in 5i/L/FA medium, we removed single kinase inhibitors or growth factors from a clonal line of OCT4-ΔPE-GFP+ cells derived in the absence of transgenes. Withdrawal of the MEK inhibitor PD0325901 or BRAF inhibitor SB590885 resulted in the rapid and widespread loss of colony morphology (Figure S4D), OCT4-ΔPE-GFP reporter activity (Figure 4G), and pluripotency gene expression (Figure 4H and Figure S4E). Withdrawal of the SRC inhibitor WH-4-023 caused a change in morphology (Figure S4D) and a slight reduction in OCT4-ΔPE-GFP reporter activity (Figure 4G). In addition, withdrawal of the ROCK inhibitor Y-27632 caused a significant reduction in proliferation (Figure S4D). On the other hand, withdrawal of recombinant FGF had no apparent effect on any of the parameters examined, which is consistent with this growth factor being involved in the maintenance of primed pluripotency. Similarly, withdrawal of Activin A did not cause a reduction in OCT4-ΔPE-GFP activity (Figure 4G). However, we observed more differentiation and reduced expression of pluripotency genes when FGF and Activin A were removed together (Figure 4H and Figures S4D and S4E). Surprisingly, reporter activity and pluripotency gene expression were unaffected by the removal of either GSK3 inhibition or hLIF (Figures 4G–4H and Figure S4E). Therefore, the maintenance of OCT4-ΔPE-GFP reporter activity is dependent primarily on MEK inhibition and BRAF inhibition, while robust proliferation of GFP+ cells requires

Figure 3. Optimization of Medium for Maintaining Viable OCT4-ΔPE-GFP+ Cells

(A) Flow cytometric analyses of the proportion of OCT4-ΔPE-GFP+ cells in a 96-well plate 1 week after culture in 2i/L/DOX, 2i/L alone, or 2i/L/SB590885 (1 μM). Top panel shows quantification of OCT4-ΔPE-GFP+ cells without including live/dead discrimination. Bottom panel shows quantification of OCT4-ΔPE-GFP+ cells after gating out DAPI+ cells.

(B) Strategy for screening a kinase inhibitor library to identify compounds that improve the fraction of viable (DAPI−) OCT4-ΔPE-GFP+ cells maintained without DOX for two passages in 2i/L/SB590995 (1 μM).

(C) Raw data obtained from high-throughput flow cytometric analysis of the proportion of DAPI−/OCT4-ΔPE-GFP+ cells in 96-well plates supplemented with one plate of a kinase inhibitor library (n = 2). Hit compound WH-4-023 is indicated with chemical structure (Martin et al., 2006).

(D and E) High-throughput flow cytometric quantification of the proportion of DAPI−/OCT4-ΔPE-GFP+ cells in 96 wells cultured for one passage (D) or two passages (E) in 64 different concentrations of PD0325901, CHIR99021, and SB590885. Asterisk denotes score of the standard concentration of the three inhibitors used in the preceding experiments (1 μM PD0325901, 3 μM CHIR99021, and 1 μM SB590885). Arrows indicate concentrations producing the highest score at P2-DOX.

(F) Phase and fluorescence images (top) and flow cytometric analysis of the proportion of OCT4-ΔPE-GFP+ cells (bottom) in a clonal line of OCT4-ΔPE-GFP+ cells derived in 2i/L/DOX and maintained for two passages without DOX in 2i/L/SB590885^{opt}/Y-27632 or 2i/L/SB590885^{opt}/Y-27632/WH-4-023. Opt, optimized concentrations of PD0325901, CHIR99021 and SB590885 (see Figure 3E). 100× magnification.

(G) Phase and fluorescence images of a clonal line of WIBR3 OCT4-ΔPE-GFP+ cells (left) and a clonal line of wild-type WIBR3 human ESCs generated in 2i/L/DOX (right) and maintained for three passages in PD0325901/IM12/SB590885/Y-27632/WH-4-023 (5i) and hLIF. 40× magnification.

(H) Teratoma generated from wild-type WIBR3 human ESCs maintained in PD0325901/IM12/SB590885/Y-27632/WH-4-023 (5i) and hLIF after transgene withdrawal. Representative tissues of the three germ layers are indicated. 200× magnification.

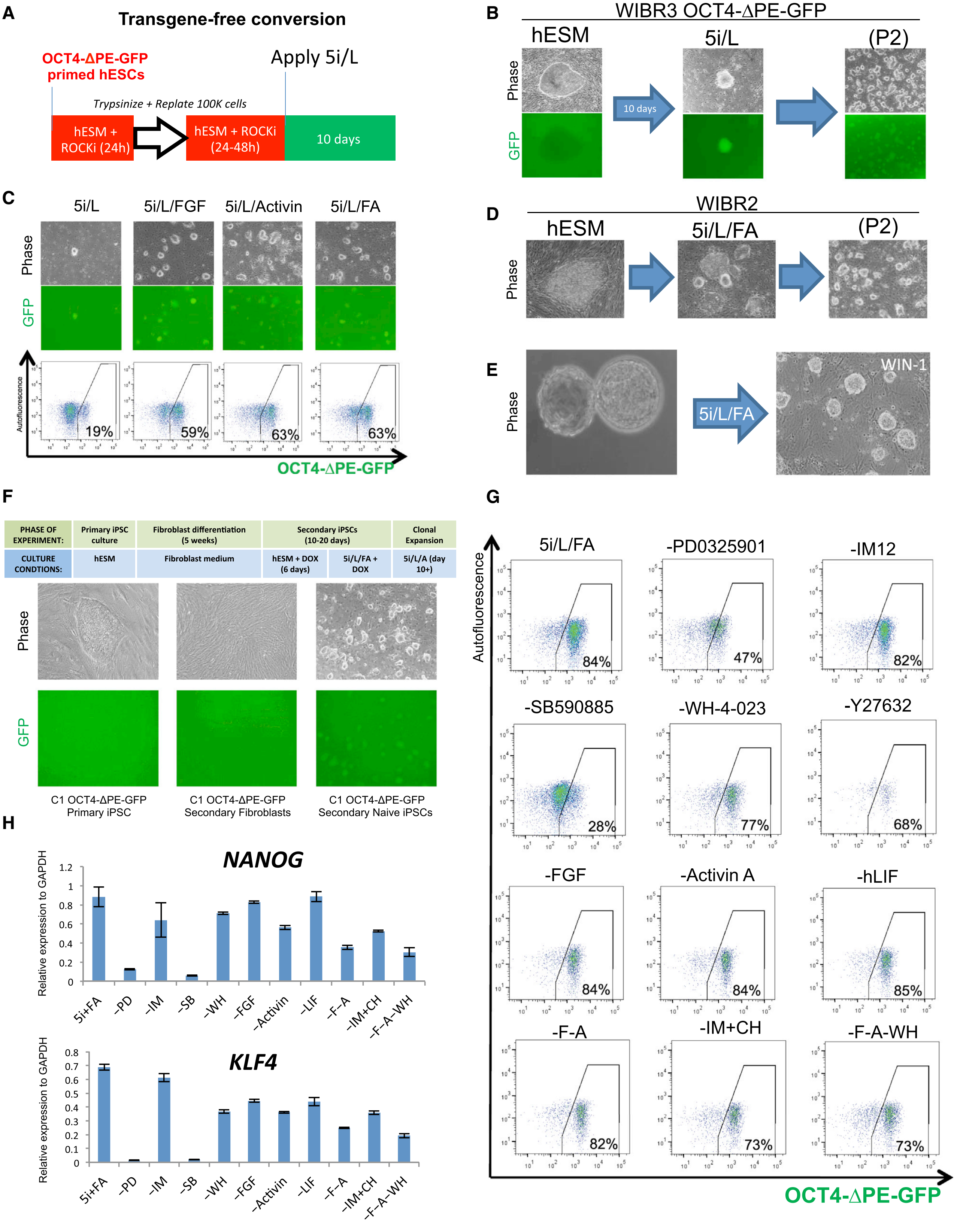

(legend on next page)

ROCK inhibition. Recombinant FGF enhances the induction of naive reporter activity but can be omitted in established GFP+ cells. Because the cells were grown on feeders we formally cannot exclude the possibility that factors produced by the MEFs contributed to cell growth.

Evaluation of Alternative Culture Systems for Naive Human Pluripotency

Recently, several groups reported alternative conditions for inducing a naive pluripotent state in conventional human ESCs (Chan et al., 2013; Gafni et al., 2013; Valamehr et al., 2014; Ware et al., 2014). Comparison of the culture components in these studies with our media shows both commonalities and differences (Figure 5A). All previously published protocols for naive human pluripotency include 2i. Another ubiquitous component is FGF, which is either added as a recombinant protein (Gafni et al., 2013; Valamehr et al., 2014; Ware et al., 2014) or present at high levels in mTesr basal medium (Chan et al., 2013). However, the use of additional RTK or BRAF inhibitors, the primary hits from our kinase inhibitor screen, was not previously reported. We examined whether previously published culture conditions for naive human pluripotency could activate our reporter system for endogenous *OCT4* distal enhancer activity. Remarkably, increased levels of OCT4-ΔPE-GFP activity were exclusively observed upon application of 5i/L/A (Figure 5B). Whereas the naive conditions described in Gafni et al. (2013) were capable of maintaining regular OCT4-GFP reporter activity after removal of KLF2 and NANOG expression, these conditions did not maintain OCT4-ΔPE-GFP activity (Figure 5C). This result is consistent with the observation that none of six JNK inhibitors and seven p38 MAP kinase inhibitors present in the kinase inhibitor library showed an ability to maintain OCT4-ΔPE-GFP activity after the withdrawal of KLF2 and NANOG expression (Figure 2B and Figure S2A). These findings suggest that our combination of kinase inhibitors induces a distinct state of human pluripotency.

We then considered whether the kinase inhibitors reported by Hanna and colleagues may have an additive effect in combination with our 5i/L/A medium. Inclusion of the JNK inhibitor SP600125 and/or p38 MAP kinase inhibitor BIRB796 did not affect the proportion of OCT4-ΔPE-GFP+ cells (Figure S5A). However, an increase in expression of KLF4 and KLF2 was observed upon addition of SP600125 (Figure S5B). Another difference between the naive human studies reported to date is the use of 20% knockout serum replacement (KSR) or Albumax-containing medium (Gafni et al., 2013; Valamehr et al., 2014; Ware et al., 2014) versus serum-free N2B27 medium (this study). We therefore investigated the consequences of applying our inhibitor cocktail in the presence of 20% KSR, and we found that this switch in basal medium resulted in the rapid attenuation of OCT4-ΔPE-GFP signal concomitant with morphological changes (Figure 5D and Figure S5C). Hence, a high concentration of KSR appears to be detrimental to naive reporter activity independently of additional kinase inhibition. In fact, a reduction in pluripotency gene expression was observed upon provision of KSR or fetal bovine serum (FBS) at concentrations >5% (Figure 5E). However, including 0.5%–1% KSR in combination with 5i/L, JNK inhibition, and Activin A (6i/L/A) enhanced the efficiency of OCT4-ΔPE-GFP induction from the primed state (Figure 5F). We conclude that the induction and maintenance of OCT4-ΔPE-GFP+ human ESCs is highly sensitive to the choice of basal medium.

Molecular Characterization of Naive Human Pluripotency

To characterize the gene expression profile of naive human ESCs derived under our conditions, RNA was collected from WIBR2 and WIBR3 human ESCs cultured in primed medium or 6i/L/A and embryo-derived Whitehead Institute Naive Human 1 (WIN1) ESCs cultured in 5i/L/A or 6i/L/A. We then performed expression analysis on Affymetrix arrays using RNA spike-in normalization (Lovén et al., 2012). Cross-species gene expression comparison demonstrated that primed WIBR2 and WIBR3 human ESCs clustered with primed mouse EpiSCs, while naive human ESCs cultured in 5i/L/A or 6i/L/A clustered with naive mouse ESCs (Figure 6A). Our naive human ESC samples clustered together closely, and addition of the JNK inhibitor had little impact on overall gene expression in the naive state (Figure 6A). The most upregulated gene ontology (GO) categories in the naive state were associated with transcriptional control, while the most downregulated categories were implicated in neural differentiation and cell adhesion (Figure S6A). Intriguingly, our naive human ESCs exhibited marked downregulation of the transcription factors OTX2 and ZIC2/3, which were recently shown to direct Oct4 to primed state-specific enhancer sites (Buecker et al., 2014). A number of transcription factors typically associated with the self-renewal and pluripotency of mouse ESCs ranked

Figure 4. Direct Conversion of Conventional Human ESCs to Naive Pluripotency in 5i/L

(A) Strategy for assessing direct conversion of primed human ESCs into OCT4-ΔPE-GFP+ cells under optimized chemical conditions.

(B) Phase and fluorescence images of emerging naive colony and expanded cells from WIBR3 OCT4-ΔPE-GFP human ESCs treated with 5i/L for 10 days. Left and right panels are 40× magnification, and middle panel is 100×.

(C) Phase and fluorescence images and flow cytometric analyses of the proportion of GFP+ cells during conversion experiments in 5i/L supplemented with FGF and/or Activin A (FA). 40× magnification.

(D) Phase images of wild-type naive WIBR2 human ESCs converted in 5i/L supplemented with FGF and/or Activin A (FA). 40× magnification.

(E) Phase image of a primary human ESC line derived in 5i/L/FA from an explanted human blastocyst. Cell line is designated as Whitehead Institute Naive Human ESC line 1 (WIN1). 100× magnification.

(F) Top, green: strategy for generating secondary naive human iPSCs from secondary derived fibroblasts (Hockemeyer et al., 2008) harboring inducible OCT4, SOX2 and KLF4 transgenes and *OCT4-ΔPE-GFP* allele. Top, blue: cell culture media conditions used at representative stages of reprogramming experiment. Bottom: phase and fluorescence images of primary primed iPSCs and secondary derived fibroblasts and the reactivation of GFP in naive reprogrammed secondary iPSCs. 40× magnification.

(G) Flow cytometric analysis of the proportion of OCT4-ΔPE-GFP+ cells three passages after withdrawal of individual inhibitors and growth factors.

(H) Quantitative gene expression analysis for NANOG and KLF4 three passages after withdrawal of individual inhibitors and growth factors from 5i/L/FA control cells. Error bars indicate ± 1 SD of technical replicates.

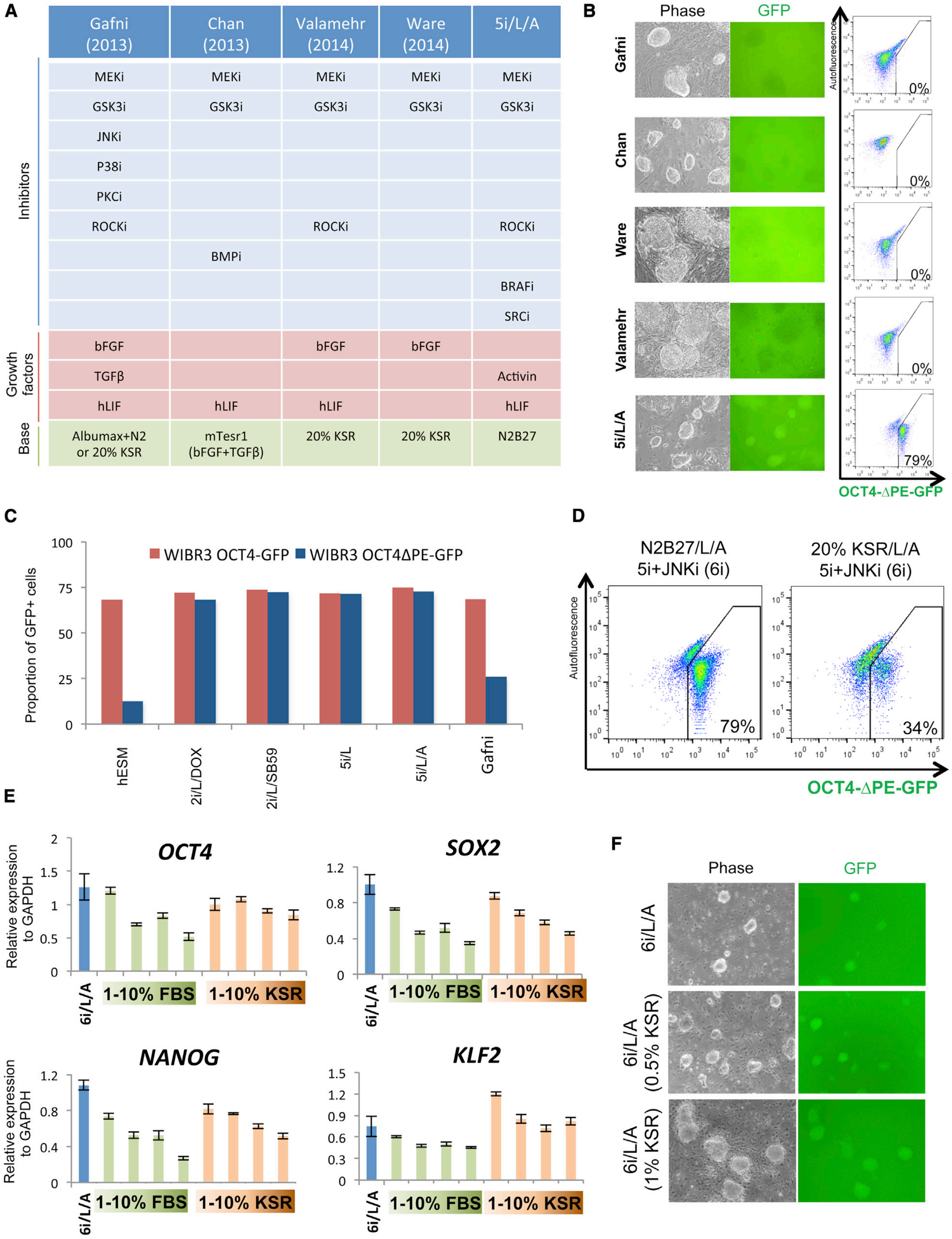

(legend on next page)

among the most highly upregulated genes in 5i/L/A or 6i/L/A, including DPPA5, DPPA3 (also known as STELLA), DPPA2, REX1, KLF4, KLF5, TFCP2L1, and NANOG (Figures 6B–6D and Table S1 available online). Expression of these factors was largely unaffected in the conditions for naive human pluripotency described by Gafni et al. (2013) and Ware et al. (2014), whereas several transcripts associated with naive pluripotency were upregulated, though not as significantly, in the conditions of Chan et al. (2013) (Figure 6B). Upregulation of transcripts specific to naive pluripotency under our conditions was confirmed by qRT-PCR (Figure 6E and Figure S6B). However, we noticed that expression of ESRRB was not significantly affected in 5i/L/A compared to the primed state. This suggests that not all aspects of the naive transcriptional program are conserved between mice and humans. A recent single-cell RNA-Seq analysis revealed that markers of naive pluripotency were highly upregulated at the morula and epiblast stages of human preimplantation development compared to conventional (primed) human ESCs (Yan et al., 2013) (Figure S6C). This suggests that our conditions may reestablish an early preimplantation epiblast-specific gene expression signature that is lost during derivation of human ESCs under conventional conditions. Hence, 5i/L/A induces a unique transcriptional profile in human ESCs, characterized by upregulation of naive-specific transcription factors and suppression of neural differentiation genes.

It was surprising that, compared to XIST levels in primed hESM conditions, *XIST* was upregulated in female WIBR2 and WIBR3 human ESCs cultured under our optimized conditions for naive human pluripotency (Table S1). This raises the question of whether the process was associated with X inactivation. Indeed, consistent with *XIST* upregulation, analysis of X-linked gene expression indicated that both female human ESC lines had undergone X chromosome inactivation upon conversion in 6i/L/A as shown in Figure 6F. A moving averages expression plot shows that the level of X-linked gene expression, while substantially higher in the starting primed cells, was reduced to a level identical to that seen in male cells after conversion to the naive state. These results raise the possibility that X inactivation in naive pluripotent cells is different in the human as compared to the mouse system.

A defining feature of ground state pluripotency in the mouse system is that transcriptional regulators such as *Nanog* are expressed homogeneously (Wray et al., 2010). We therefore investigated whether human ESCs cultured under our conditions are more homogeneous with respect to expression of NANOG by sm RNA FISH analysis. The mean number of OCT4 mRNAs per cell was approximately similar between WIBR2 human ESCs in primed medium, the naive medium of Gafni et al. (2013), and 5i/L/A (Figure 6G and Figure S7A). As expected from array and qRT-PCR analyses (Figures 6C and 6E), the mean number of NANOG mRNAs per cell was significantly higher in 5i/L/A (Figure 6G and Figure S7A). Intriguingly, 5i/L/A culture also resulted in reduced cell-to-cell variability in NANOG expression compared to both primed medium and the naive medium of Gafni et al. (2013) (Figure S7B). Thus, the increased expression level of NANOG in 5i/L/A does not arise from a subset of cells, but it is uniform across the population. We also confirmed by RNA FISH that single cells cultured in 5i/L/A express significantly higher numbers of KLF4 and REX1 mRNAs (Figure 6G). In conclusion, our data suggest that expression profiling can serve as an informative benchmark to distinguish different states of primed and naive human pluripotency.

We performed chromatin immunoprecipitation followed by DNA sequencing (ChIP-Seq) analysis to determine the genome-wide distribution of the activation-associated marker trimethylation of histone 3 lysine 4 (H3K4me3) and the transcriptional-silencing-associated marker trimethylation of histone 3 lysine 27 (H3K27me3). Developmental genes with bivalent domains marked by the presence of both H3K4me3 and H3K27me3 in the primed state, such as *HOXA9*, *FOXA2*, and *GATA6*, exhibited a reduced H3K27me3 signal in the naive state (Figure 7A). We then examined the histone methylation profile at loci encoding naive-specific pluripotency regulators. *KLF2*, *KLF4*, and *KLF5* were bivalent in the primed state, but they almost entirely lost the H3K27me3 mark in the naive state (Figure 7B). Other markers of ground state pluripotency that are highly upregulated in our system, such as *DPPA5*, *DPPA3*, and *REX1*, acquired the H3K4me3 signal during conversion from primed to naive pluripotency (Figure 7C). The core pluripotency determinants *OCT4*, *SOX2*, and *NANOG* were marked exclusively by H3K4me3 in naive and primed human ESCs (Figure 7D). However, a slightly higher H3K4me3 signal was observed at the *NANOG* promoter in the naive state, consistent with increased transcription (Figure 6E). Finally, while H3K27me3 was generally depleted from promoter regions in the naive state, some genes that were strongly downregulated acquired H3K27me3 signal (Figure 7E). Consistent with observations in naive pluripotent mouse ESCs (Marks et al., 2012), H3K27me3 was strongly reduced at the transcriptional start site (TSS) of Polycomb group target genes in naive human ESCs (Figures 7F and 7G). We conclude that the conversion from primed to naive human pluripotency is accompanied by the dynamic rearrangement of

Figure 5. Evaluation of Alternative Culture Conditions for Naive Human Pluripotency

(A) Table comparing the components of four recent protocols for capturing naive-like human ESCs with 5i/L/A medium. Note that the protocol for naive conversion from Ware et al. (2014) involves preculture with the HDAC inhibitors butyrate and suberoylanilide hydroxamic acid.

(B) Phase and fluorescence images and flow cytometric analyses showing the response of OCT4-ΔPE-GFP− primed cells to recently reported protocols for naive human pluripotency (see Figure 5A) and 5i/L/A. 40× magnification.

(C) Quantification of the proportion of GFP+ cells in WIBR3 OCT4-GFP and OCT4-ΔPE-GFP human ESCs upon removal of DOX-inducible KLF2 and NANOG expression in primed human ES medium (hESM) and four alternative conditions for naive human pluripotency.

(D) Flow cytometric analysis of the proportion of OCT4-ΔPE-GFP+ cells in 5i/L/A and the JNK inhibitor SP600125 (6i/L/A) in serum-free N2B27 basal medium versus 20% KSR basal medium.

(E) Quantitative gene expression analysis for OCT4, SOX2, KLF2, and NANOG in human ESCs cultured in 6i/L/A and supplemented with 1%, 5%, 7.5%, or 10% FBS or KSR. Error bars indicate ± 1 SD of technical replicates.

(F) Phase and fluorescence images of induction of OCT4-ΔPE-GFP activity from the primed state in 6i/L/A, and 6i/L/A supplemented with 1%, 5%, 7.5%, or 10% KSR. 40× magnification.

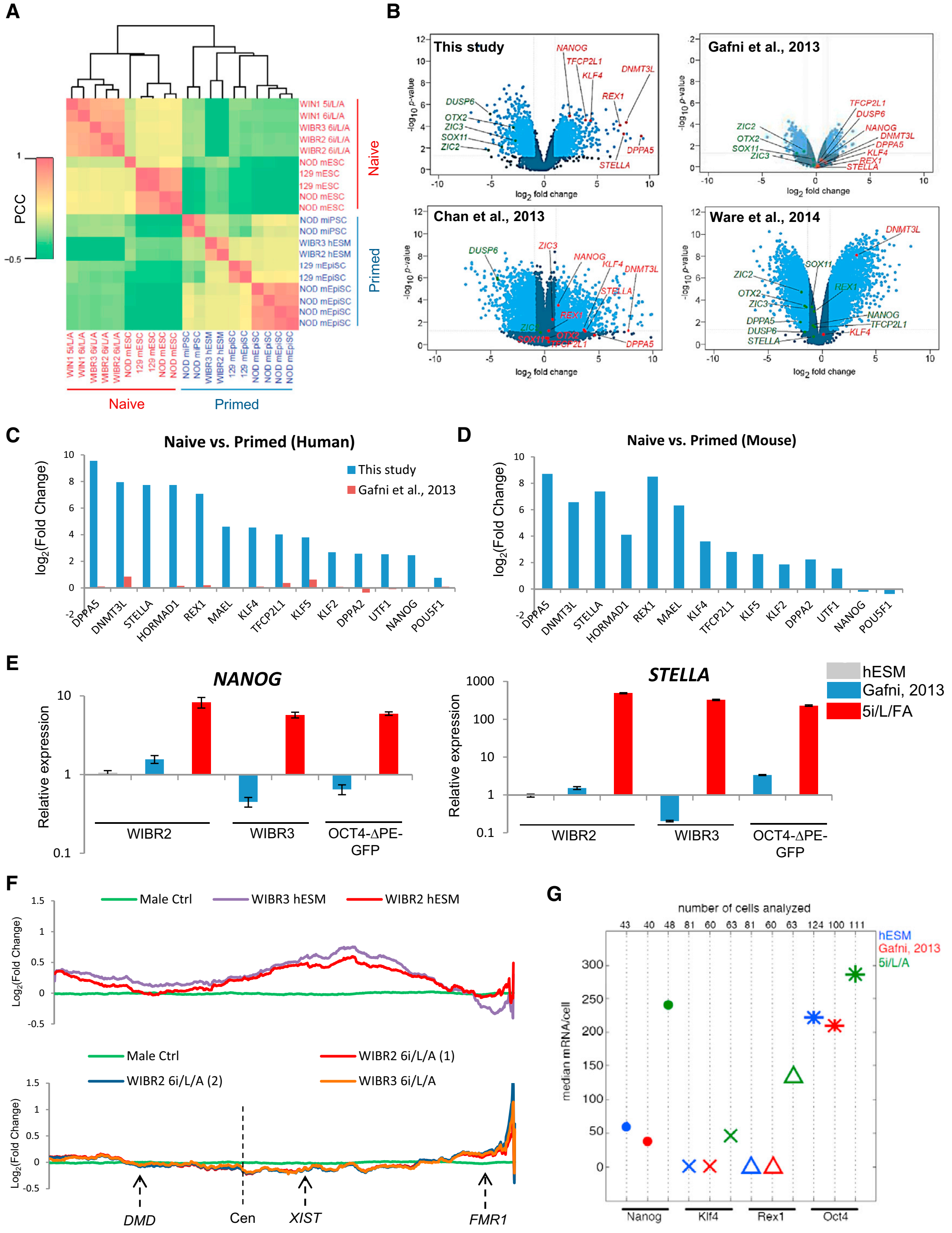

Figure 6. Transcriptional Profiling of Naive Human ESCs in 5i/L/A

(A) Cross-species hierarchical clustering of naive and primed pluripotent cells from mice and humans, as performed previously by Gafni et al. (2013). Affymetrix expression data were normalized using RNA spike-in. Two groups of human ESC samples are included: WIBR2 (P12 and P14), WIBR3 (P9), and WIN1 (P10) human ESCs derived in our optimized naive medium (5i/L/A or 6i/L/A, as indicated) and parental WIBR2 and WIBR3 human ESCs in primed human ESC medium (hESM). Correlation matrix of gene expression was clustered using Pearson correlation coefficients (PCCs). The average linkage hierarchical clustering of the Pearson correlation is shown in the heatmap. mEpiSCs, mouse EpiSCs; mESC, mouse ESC; miPSC, mouse iPSC. Note that NOD miPSC samples described in Hanna et al. (2009) were metastable and acquired an EpiSC-like identity after undergoing removal of exogenous transcription factors.

(B) Volcano plots showing fold change (x axis) between naive human ESC samples and primed human ESCs on all genes, as reported in this study, Gafni et al. (2013), Chan et al. (2013), and Ware et al. (2014). The light blue dots represent those genes that exhibit the most significant gene expression changes (defined as

(legend continued on next page)

activating and repressive histone modifications. In particular, transcriptional upregulation of specific regulators of ground state pluripotency is associated with the gain of H3K4me3 or loss of H3K27me3 in a locus-specific manner.

Developmental Potential of Human ESCs in 5i/L/A

The classical assay for pluripotency of human ESCs and iPSCs is to examine teratomas formed after their subcutaneous injection into NOD/SCID mice. We found that naive human ESCs converted under our optimized conditions contributed to high-grade teratomas containing tissues representing all three germ layers, regardless of the presence of FGF and Activin A in the medium (Figure S7C). We also investigated the suitability of naive human ESCs in directed differentiation. For this purpose we subjected human ESCs derived from WIBR2 and WIBR3 primed cells in 5i/L/A to a five-step protocol for hepatic differentiation (Si-Tayeb et al., 2010). We readily generated hepatocytes positive for the mature markers AFP and HNF4a from both lines of naive ESCs (Figure S7D).

In the mouse system, naive ESCs are functionally distinguished by their capability to colonize the embryo and contribute to chimeric animals. In contrast, primed EpiSCs contribute only very inefficiently to chimeras (Brons et al., 2007; Tesar et al., 2007). Hanna and colleagues reported that human ESCs cultured in the presence of 2i, hLIF, JNK inhibition, p38 inhibition, FGF, and TGFβ contributed robustly to interspecies chimeric embryos after injection into mouse morulae (Gafni et al., 2013), a result that provided a defining functional read-out for naive human pluripotency. Therefore, we attempted to reproduce this experiment using human ESCs containing constitutive GFP or tdTomato transgenes in the *AAVS1* locus. In total, we injected 860 embryos (eight-cell, morula, and blastocyst stages) with GFP or tdTomato-labeled human ESCs cultured in 5i/L/A. We also injected 436 embryos with C1-AAVS1-GFP cells provided by the Hanna laboratory, which were cultured using the conditions reported by Gafni et al. (2013). We recovered only a fraction of embryos (43%–45%) from both experimental groups at E10.5, suggesting that the majority of embryos had been reabsorbed. No fluorescent signal was detected by microscopy in any embryos recovered at E10.5 (Figure S7E). Thus, contribution of human ESCs in 5i/L/A or the medium of Gafni et al. (2013) to interspecies chimeras is in our hands too inefficient for detection by standard visual inspection. Because interspecific chimera formation is a complex and demanding assay, it is possible that slight variations in cell culture conditions or embryo handling could explain the different results.

DISCUSSION

The morphological, molecular, and functional similarity between human ESCs and mouse postimplantation epiblast-derived EpiSCs has prompted widespread interest in capturing the equivalent of a naive pluripotent stem cell in humans (De Los Angeles et al., 2012; Nichols and Smith, 2009). At the outset of this study, we considered that the identification of putative naive human ESCs would be greatly facilitated by the availability of a specific reporter system. In the mouse, enhancer-specific regulation of *Oct4* expression is a defining molecular distinction between ESCs and EpiSCs in vitro (Brons et al., 2007; Tesar et al., 2007) and between the inner cell mass (ICM) and postimplantation epiblast in vivo (Yeom et al., 1996). We therefore deleted the primed-specific PE from an *OCT4-2A-GFP* allele using TALEN-mediated genome editing in heterozygously targeted $OCT4^{WT/GFP}$ human ESCs (Hockemeyer et al., 2011). The observation that GFP expression was downregulated after PE removal demonstrated that this reporter behaves as expected in the primed state. Conversely, OCT4-ΔPE-GFP activity was strongly induced by combined overexpression of KLF2 and NANOG. We then took a systematic approach to screen a diverse collection of 230 kinase inhibitors for their capacity to maintain OCT4-ΔPE-GFP activity after the removal of ectopic KLF2 and NANOG expression. Through iterative screening we identified a combination of five kinase inhibitors that maintained viable GFP+ cells upon transgene withdrawal. Moreover, this kinase inhibitor cocktail was capable of inducing OCT4-ΔPE-GFP activity when applied directly to conventional (primed) human ESCs in the complete absence of reprogramming factors.

Previous studies describing the isolation of naive human ESCs also reported transgene-free interconversion from primed to naive pluripotency (Chan et al., 2013; Gafni et al., 2013; Valamehr et al., 2014; Ware et al., 2014). However, these published protocols did not induce endogenous OCT4-ΔPE-GFP activity in our hands. This finding was surprising given that Gafni et al. (2013) reported activation of luciferase and BAC reporter constructs under the control of the DE of human *OCT4*, while Ware et al. (2014) observed increased DNaseI hypersensitivity at the DE in human ESCs derived in 2i and FGF. For a possible explanation we speculate that activation of the DE of endogenous *OCT4* may represent a more stringent criterion for the naive pluripotent state than activation of a transgene. A case in point is the ready activation of an Oct4 reporter transgene in the absence of endogenous *Oct4* expression (Hou et al., 2013). We also noticed a difference in the kinetics of primed to naive conversion using

those that have a log2 fold change > 1 and < −1 and wherein $p < 0.05$). Highlighted in red or green are genes of interest that are upregulated in the naive state or downregulated in the naive state, respectively.

(C) Fold changes in expression of naive pluripotency-associated transcripts in our naive human ESC samples versus primed human ESCs (blue), and the naive human samples published by Gafni et al. (2013) versus primed human ESCs (red).

(D) For comparison with (C), fold changes in expression of naive pluripotency-associated transcripts in naive mouse ESCs versus primed mouse EpiSCs were curated from a previously published study (Najm et al., 2011).

(E) Quantitative gene expression analysis for NANOG and STELLA in human ESCs cultured in parallel in primed medium, the medium of Gafni et al. (2013), and 5i/L/FA (P4). Error bars indicate ± 1 SD of technical replicates.

(F) Moving average plots of expressed genes along the X chromosome in female primed human ESCs (WIBR2 and WIBR3 hESM) compared to the average of three male control iPSCs in hESM (top) and converted naive human ESCs in 6i/L/A (bottom). Shown are representative genes on the X chromosome and the location of the centromere (Cen).

(G) Single-molecule (sm) RNA FISH analysis using OCT4, NANOG, KLF4, and REX1 probes in human ESCs cultured in primed hESM, the medium of Gafni et al. (2013), or 5i/L/A.

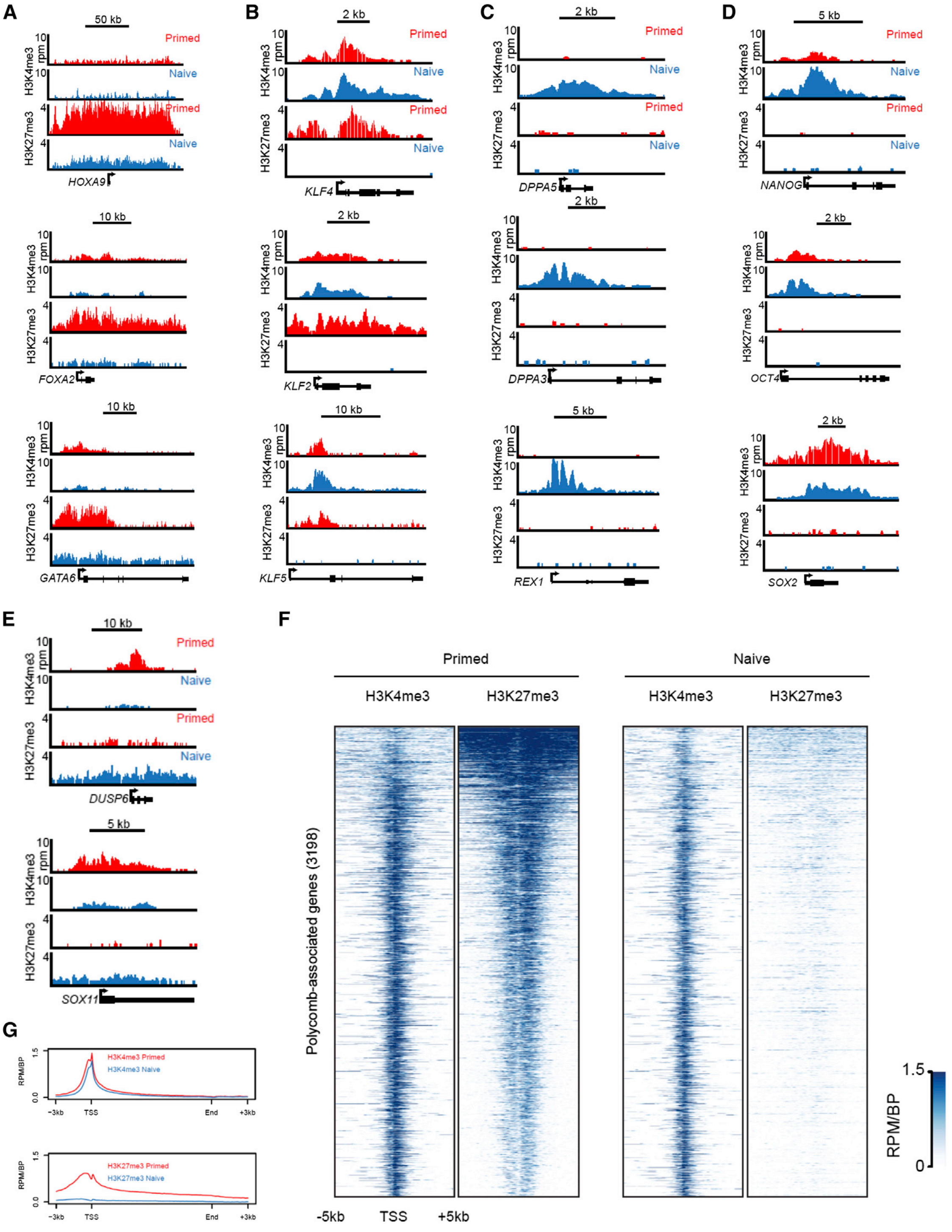

Figure 7. Chromatin Landscape of Naive Human Pluripotency

(A–E) ChIP-Seq tracks for H3K4me3 and H3K27me3 in WIBR2 human ESCs cultured in primed hESM (red) or naive 6i/L/A medium (P18) (blue) at five classes of genes: (A) developmental genes that are bivalent in the primed state and exhibit loss of H3K27me3 in the naive state; (B) naive pluripotency genes that are bivalent in the primed state and exhibit loss of H3K27me3 in the naive state; (C) naive pluripotency genes that acquire H3K4me3 in the naive state; (D) master transcription factors that have a signal for H3K4me3, but not H3K27me3, in both naive and primed states; and (E) genes that are strongly downregulated in the naive state and acquire increased H3K27me3 signal.

(F) ChIP-Seq analysis for H3K4me3 and H3K27me3 at Polycomb target genes in WIBR2 human ESCs cultured in primed medium (left) or naive 6i/L/A medium (right).

(G) Average H3K4me3 and H3K27me3 signal at Polycomb target genes in WIBR2 human ESCs cultured in primed medium (red) or naive 6i/L/A medium (blue).

previously reported protocols: whereas the emergence of naive colonies under our conditions is a relatively protracted process (ca. 10 days) that occurs after widespread cell death, the application of previously described naive conditions was accompanied with little cell death and rapid expansion of the cells. The slow conversion observed in 5i/L/A is consistent with a reprogramming event toward a distinct cellular state, rather than adaptation to an identity closer to conventional (primed) human ESCs. Critically, our conditions also induced a dramatic upregulation of transcription factors typically associated with naive pluripotency and human preimplantation development. Thus, our observations suggest that the chemical screens used in this study have identified a distinct state of human pluripotency.

Our observation that conversion of primed human ESCs into a naive state is associated with upregulation of *XIST* and inactivation of X-linked gene expression is unexpected because conversion of mouse EpiSCs to the naive state leads to X chromosome reactivation (Guo et al., 2009; Silva et al., 2009). Previous observations demonstrating that *XIST* is transcribed in the ICM of human blastocysts (Okamoto et al., 2011) suggested that the process of X inactivation in human embryos is different from that seen in mouse and that the regulatory intersection between induction of the naive pluripotency circuitry and X chromosome reactivation may not be conserved from mice to humans. However, our results must be interpreted with caution because they are based on conversion of extensively passaged primed human ESCs, which may have undergone X chromosome erosion (Mekhoubad et al., 2012). A more thorough examination of the state of X inactivation will require the direct derivation of naive ESCs from female human blastocysts (the ESC lines derived in this study were all male).

This work suggests that the concept of naive human pluripotency may need to be reevaluated. Previously described protocols for capturing naive human ESCs may offer practical advantages over conventional human ESCs, including enhanced proliferation and single-cell cloning, which could benefit applications such as gene targeting. As such, these studies are of considerable interest because they may facilitate the application of human iPSCs in disease modeling and regenerative medicine. However, here we describe a distinct state of human pluripotency with a transcriptional and epigenomic profile that closely resembles naive mouse pluripotency. This suggests that human ESCs can exist in different pluripotent states with distinct biological and functional characteristics.

Our work opens several avenues for future experimental work. First, while the culture conditions described here generate pluripotent cells that more closely approximate the naive state of mouse ESCs, they fall short of establishing a ground state in which self-renewal is fully independent of extrinsic instruction (Ying et al., 2008). Thus, conditions will need to be developed that support propagation of naive human ESCs in the absence of feeders, recombinant growth factors, and cytokines. Second, naive human ESCs derived in 5i/L/A proliferate slowly when compared to mouse ESCs. Thus, it will be interesting to identify culture conditions that lead to higher proliferation. In addition, the long-term karyotypic stability of human ESCs in 5i/L/A needs to be assessed. Finally, the examination of naive human pluripotency is currently complicated by the absence of a defining functional assay. The generation of interspecies chimeras by injection of human ESCs into mouse morulae was proposed as a stringent assay for naive human pluripotency (Gafni et al., 2013), but in our hands this assay proved too inefficient as a routine functional assay. Further improvements in the culture methods for naive human ESCs and techniques for whole-embryo imaging may provide a better platform for assessing the developmental potential of naive human ESCs in vivo. Alternatively, the use of our optimized culture conditions to isolate chimera-competent naive ESCs from nonhuman primates could provide more rigorous evidence of naive pluripotency.

EXPERIMENTAL PROCEDURES

Cell Culture

Conventional (primed) human iPSC line C1 (Whitehead Institute Center for Human Stem Cell Research, Cambridge, MA) (Hockemeyer et al., 2008) and human ESC lines WIBR2 and WIBR3 (Whitehead Institute Center for Human Stem Cell Research, Cambridge, MA) (Lengner et al., 2010) were maintained on mitomycin C inactivated MEF feeder layers and passaged mechanically using a drawn Pasteur pipette or enzymatically by treatment for 20 min with 1 mg/ml Collagenase type IV (GIBCO) followed by sequential sedimentation steps in human ESC medium (hESM) to remove single cells. Primed human ESCs and human iPSCs were cultured in hESM—DMEM/F12 (Invitrogen) supplemented with 15% FBS (Hyclone), 5% KSR (Invitrogen), 1 mM glutamine (Invitrogen), 1% nonessential amino acids (Invitrogen), penicillin-streptomycin (Invitrogen), 0.1 mM β-mercaptoethanol (Sigma), and 4 ng/ml FGF2 (R&D systems). Naive human ESCs/hiPSCs were cultured on mitomycin C-inactivated MEF feeder cells and were passaged every 5–7 days by a brief PBS wash followed by single-cell dissociation using 3–5 min treatment with Accutase (GIBCO) and centrifugation in fibroblast medium (DMEM [Invitrogen] supplemented with 10% FBS [Hyclone], 1 mM glutamine [Invitrogen], 1% nonessential amino acids [Invitrogen], penicillin-streptomycin [Invitrogen], and 0.1 mM β-mercaptoethanol). For conversion of preexisting primed human ESC lines, we seeded 2×10^5 trypsinized single cells on an MEF feeder layer in hESM supplemented with ROCK inhibitor Y-27632 (Stemgent, 10 μM). One or two days later, medium was switched to 5i/L/A-containing naive hESM. Following an initial wave of widespread cell death, dome-shaped naive colonies appeared within 10 days and could be picked or expanded polyclonally using 3–5 min treatment with Accutase (GIBCO) on an MEF feeder layer. Naive human pluripotent cells were derived and maintained in serum-free N2B27-based media supplemented with 5i/L/A. Medium (500 ml) was generated by inclusion of the following: 240 ml DMEM/F12 (Invitrogen; 11320), 240 ml Neurobasal (Invitrogen; 21103), 5 ml N2 supplement (Invitrogen; 17502048), 10 ml B27 supplement (Invitrogen; 17504044), 10 μg recombinant human LIF (made in-house), 1 mM glutamine (Invitrogen), 1% nonessential amino acids (Invitrogen), 0.1 mM β-mercaptoethanol (Sigma), penicillin-streptomycin (Invitrogen), 50 μg/ml BSA (Sigma), and the following small molecules and cytokines: PD0325901 (Stemgent, 1 μM), IM-12 (Enzo, 1 μM), SB590885 (R&D systems, 0.5 μM), WH-4-023 (A Chemtek, 1 μM), Y-27632 (Stemgent, 10 μM), and Activin A (Peprotech, 20 ng/ml). 0.5% KSR (GIBCO) can be included to enhance conversion efficiency. FGF2 (R&D systems, 8 ng/ml) enhanced the generation of OCT4-ΔPE-GFP+ cells from the primed state, but it was dispensable for maintenance of naive human ESCs. Additional chemicals described in this work include: CHIR99021 (Stemgent, 0.3–3 μM as indicated), SP600125 (R&D systems, 10 μM), PD173074 (Stemgent, 0.1 μM), SB431542 (Tocris, 5 μM), BIRB796 (Axon Medchem, 2 μM), and doxycycline (Sigma-Aldrich, 2 μg/ml). Tissue culture media were filtered using a low protein-binding binding 0.22 μM filter (Corning). Alternative formulations for naive human ESC culture were followed as described elsewhere (Chan et al., 2013; Gafni et al., 2013; Valamehr et al., 2014; Ware et al., 2014). All experiments in this paper were performed under physiological oxygen conditions (5% O_2, 3% CO_2) in the presence of a MEF feeder layer unless stated otherwise.

ACCESSION NUMBERS

The GEO accession number for the gene expression and ChIP-Seq analyses in this paper is GSE59435.

SUPPLEMENTAL INFORMATION

Supplemental Information for this article includes Supplemental Experimental Procedures, seven figures, and one table and can be found with this article online at http://dx.doi.org/10.1016/j.stem.2014.07.002.

AUTHOR CONTRIBUTIONS

T.W.T., B.E.P., H.W., and R.J. conceived of the study and designed experiments. H.W. performed gene targeting experiments. T.W.T and B.E.P. performed chemical screens with assistance from J.Z. and N.S.G. and optimized the conditions for naive human pluripotency. M.M. performed ESC derivation. T.W.T., B.E.P., D.A.F., J.R., Z.P.F., T.L., S.I., Y.S., S.R., A.D., and R.A.Y. analyzed molecular properties of naive human ESCs. T.W.T., D.M., K.G., L.S., M.M.D., and Q.G. assessed the developmental potential of naive human ESCs. T.W.T. wrote the manuscript with input from B.E.P. and R.J.

ACKNOWLEDGMENTS

We thank Dongdong Fu for sectioning, Raaji Alagappan and Ping Xu for preparation of MEFs, Ruth Flannery for embryo processing, Patti Wisniewski and Colin Zollo for cell sorting, David Sabatini and Kathleen Ottina for use of the Zymark Liquid Handling system, the Whitehead Genome Technology Core for assistance with expression array and ChIP-Seq analysis, and Jacob Hanna (Weizmann Institute) for providing C1-AAVS1-GFP human ESCs. This study was supported by a grant from the Simons Foundation (SFLIFE #286977 to R.J) and was supported in part by NIH grant RO1-CA084198 to R.J. T.W.T. is supported by a Sir Henry Wellcome Postdoctoral Fellowship (098889/Z/12/Z). B.E.P. is supported by a Boehringer Ingelheim Fonds Ph.D. Fellowship and a Jerome and Florence Brill Graduate Student Fellowship. D.A.F. is supported by a National Science Foundation Graduate Research Fellowship and a Jerome and Florence Brill Graduate Student Fellowship. Y.S. is supported by a Human Frontier Science Program Postdoctoral Fellowship. R.J. is a cofounder of Fate Therapeutics and an adviser to Stemgent.

Received: June 6, 2014
Revised: June 27, 2014
Accepted: July 13, 2014
Published: July 24, 2014

REFERENCES

Brons, I.G., Smithers, L.E., Trotter, M.W., Rugg-Gunn, P., Sun, B., Chuva de Sousa Lopes, S.M., Howlett, S.K., Clarkson, A., Ahrlund-Richter, L., Pedersen, R.A., and Vallier, L. (2007). Derivation of pluripotent epiblast stem cells from mammalian embryos. Nature *448*, 191–195.

Buecker, C., Srinivasan, R., Wu, Z., Calo, E., Acampora, D., Faial, T., Simeone, A., Tan, M., Swigut, T., and Wysocka, J. (2014). Reorganization of enhancer patterns in transition from naive to primed pluripotency. Cell Stem Cell *14*, 838–853.

Chan, E.M., Yates, F., Boyer, L.F., Schlaeger, T.M., and Daley, G.Q. (2008). Enhanced plating efficiency of trypsin-adapted human embryonic stem cells is reversible and independent of trisomy 12/17. Cloning Stem Cells *10*, 107–118.

Chan, Y.S., Göke, J., Ng, J.H., Lu, X., Gonzales, K.A., Tan, C.P., Tng, W.Q., Hong, Z.Z., Lim, Y.S., and Ng, H.H. (2013). Induction of a human pluripotent state with distinct regulatory circuitry that resembles preimplantation epiblast. Cell Stem Cell *13*, 663–675.

De Los Angeles, A., Loh, Y.H., Tesar, P.J., and Daley, G.Q. (2012). Accessing naïve human pluripotency. Curr. Opin. Genet. Dev. *22*, 272–282.

Gafni, O., Weinberger, L., Mansour, A.A., Manor, Y.S., Chomsky, E., Ben-Yosef, D., Kalma, Y., Viukov, S., Maza, I., Zviran, A., et al. (2013). Derivation of novel human ground state naive pluripotent stem cells. Nature *504*, 282–286.

Guo, G., Yang, J., Nichols, J., Hall, J.S., Eyres, I., Mansfield, W., and Smith, A. (2009). Klf4 reverts developmentally programmed restriction of ground state pluripotency. Development *136*, 1063–1069.

Hall, J., Guo, G., Wray, J., Eyres, I., Nichols, J., Grotewold, L., Morfopoulou, S., Humphreys, P., Mansfield, W., Walker, R., et al. (2009). Oct4 and LIF/Stat3 additively induce Krüppel factors to sustain embryonic stem cell self-renewal. Cell Stem Cell *5*, 597–609.

Hanna, J., Markoulaki, S., Mitalipova, M., Cheng, A.W., Cassady, J.P., Staerk, J., Carey, B.W., Lengner, C.J., Foreman, R., Love, J., et al. (2009). Metastable pluripotent states in NOD-mouse-derived ESCs. Cell Stem Cell *4*, 513–524.

Hanna, J., Cheng, A.W., Saha, K., Kim, J., Lengner, C.J., Soldner, F., Cassady, J.P., Muffat, J., Carey, B.W., and Jaenisch, R. (2010). Human embryonic stem cells with biological and epigenetic characteristics similar to those of mouse ESCs. Proc. Natl. Acad. Sci. USA *107*, 9222–9227.

Hirano, K., Nagata, S., Yamaguchi, S., Nakagawa, M., Okita, K., Kotera, H., Ainscough, J., and Tada, T. (2012). Human and mouse induced pluripotent stem cells are differentially reprogrammed in response to kinase inhibitors. Stem Cells Dev. *21*, 1287–1298.

Hockemeyer, D., Soldner, F., Cook, E.G., Gao, Q., Mitalipova, M., and Jaenisch, R. (2008). A drug-inducible system for direct reprogramming of human somatic cells to pluripotency. Cell Stem Cell *3*, 346–353.

Hockemeyer, D., Wang, H., Kiani, S., Lai, C.S., Gao, Q., Cassady, J.P., Cost, G.J., Zhang, L., Santiago, Y., Miller, J.C., et al. (2011). Genetic engineering of human pluripotent cells using TALE nucleases. Nat. Biotechnol. *29*, 731–734.

Hou, P., Li, Y., Zhang, X., Liu, C., Guan, J., Li, H., Zhao, T., Ye, J., Yang, W., Liu, K., et al. (2013). Pluripotent stem cells induced from mouse somatic cells by small-molecule compounds. Science *341*, 651–654.

Lengner, C.J., Gimelbrant, A.A., Erwin, J.A., Cheng, A.W., Guenther, M.G., Welstead, G.G., Alagappan, R., Frampton, G.M., Xu, P., Muffat, J., et al. (2010). Derivation of pre-X inactivation human embryonic stem cells under physiological oxygen concentrations. Cell *141*, 872–883.

Li, P., Tong, C., Mehrian-Shai, R., Jia, L., Wu, N., Yan, Y., Maxson, R.E., Schulze, E.N., Song, H., Hsieh, C.L., et al. (2008). Germline competent embryonic stem cells derived from rat blastocysts. Cell *135*, 1299–1310.

Lovén, J., Orlando, D.A., Sigova, A.A., Lin, C.Y., Rahl, P.B., Burge, C.B., Levens, D.L., Lee, T.I., and Young, R.A. (2012). Revisiting global gene expression analysis. Cell *151*, 476–482.

Marks, H., Kalkan, T., Menafra, R., Denissov, S., Jones, K., Hofemeister, H., Nichols, J., Kranz, A., Stewart, A.F., Smith, A., and Stunnenberg, H.G. (2012). The transcriptional and epigenomic foundations of ground state pluripotency. Cell *149*, 590–604.

Martin, M.W., Newcomb, J., Nunes, J.J., McGowan, D.C., Armistead, D.M., Boucher, C., Buchanan, J.L., Buckner, W., Chai, L., Elbaum, D., et al. (2006). Novel 2-aminopyrimidine carbamates as potent and orally active inhibitors of Lck: Synthesis, SAR, and in vivo antiinflammatory activity. J. Med. Chem. *49*, 4981–4991.

Meek, S., Wei, J., Sutherland, L., Nilges, B., Buehr, M., Tomlinson, S.R., Thomson, A.J., and Burdon, T. (2013). Tuning of β-catenin activity is required to stabilize self-renewal of rat embryonic stem cells. Stem Cells *31*, 2104–2115.

Mekhoubad, S., Bock, C., de Boer, A.S., Kiskinis, E., Meissner, A., and Eggan, K. (2012). Erosion of dosage compensation impacts human iPSC disease modeling. Cell Stem Cell *10*, 595–609.

Najm, F.J., Chenoweth, J.G., Anderson, P.D., Nadeau, J.H., Redline, R.W., McKay, R.D., and Tesar, P.J. (2011). Isolation of epiblast stem cells from preimplantation mouse embryos. Cell Stem Cell *8*, 318–325.

Nichols, J., and Smith, A. (2009). Naive and primed pluripotent states. Cell Stem Cell *4*, 487–492.

Okamoto, I., Patrat, C., Thépot, D., Peynot, N., Fauque, P., Daniel, N., Diabangouaya, P., Wolf, J.P., Renard, J.P., Duranthon, V., and Heard, E. (2011). Eutherian mammals use diverse strategies to initiate X-chromosome inactivation during development. Nature *472*, 370–374.

Si-Tayeb, K., Noto, F.K., Nagaoka, M., Li, J., Battle, M.A., Duris, C., North, P.E., Dalton, S., and Duncan, S.A. (2010). Highly efficient generation of human hepatocyte-like cells from induced pluripotent stem cells. Hepatology *51*, 297–305.

Silva, J., and Smith, A. (2008). Capturing pluripotency. Cell *132*, 532–536.

Silva, J., Nichols, J., Theunissen, T.W., Guo, G., van Oosten, A.L., Barrandon, O., Wray, J., Yamanaka, S., Chambers, I., and Smith, A. (2009). Nanog is the gateway to the pluripotent ground state. Cell *138*, 722–737.

Tesar, P.J., Chenoweth, J.G., Brook, F.A., Davies, T.J., Evans, E.P., Mack, D.L., Gardner, R.L., and McKay, R.D. (2007). New cell lines from mouse epiblast share defining features with human embryonic stem cells. Nature *448*, 196–199.

Theunissen, T.W., van Oosten, A.L., Castelo-Branco, G., Hall, J., Smith, A., and Silva, J.C. (2011). Nanog overcomes reprogramming barriers and induces pluripotency in minimal conditions. Curr. Biol. *21*, 65–71.

Valamehr, B., Robinson, M., Abujarour, R., Rezner, B., Vranceanu, F., Le, T., Medcalf, A., Lee, T.T., Fitch, M., Robbins, D., et al. (2014). Platform for Induction and Maintenance of Transgene-free hiPSCs Resembling Ground State Pluripotent Stem Cells. Stem Cell Reports *2*, 366–381.

Wang, W., Yang, J., Liu, H., Lu, D., Chen, X., Zenonos, Z., Campos, L.S., Rad, R., Guo, G., Zhang, S., et al. (2011). Rapid and efficient reprogramming of somatic cells to induced pluripotent stem cells by retinoic acid receptor gamma and liver receptor homolog 1. Proc. Natl. Acad. Sci. USA *108*, 18283–18288.

Ware, C.B., Nelson, A.M., Mecham, B., Hesson, J., Zhou, W., Jonlin, E.C., Jimenez-Caliani, A.J., Deng, X., Cavanaugh, C., Cook, S., et al. (2014). Derivation of naive human embryonic stem cells. Proc. Natl. Acad. Sci. USA *111*, 4484–4489.

Wray, J., Kalkan, T., and Smith, A.G. (2010). The ground state of pluripotency. Biochem. Soc. Trans. *38*, 1027–1032.

Yan, L., Yang, M., Guo, H., Yang, L., Wu, J., Li, R., Liu, P., Lian, Y., Zheng, X., Yan, J., et al. (2013). Single-cell RNA-Seq profiling of human preimplantation embryos and embryonic stem cells. Nat. Struct. Mol. Biol. *20*, 1131–1139.

Yeom, Y.I., Fuhrmann, G., Ovitt, C.E., Brehm, A., Ohbo, K., Gross, M., Hübner, K., and Schöler, H.R. (1996). Germline regulatory element of Oct-4 specific for the totipotent cycle of embryonal cells. Development *122*, 881–894.

Ying, Q.L., Wray, J., Nichols, J., Batlle-Morera, L., Doble, B., Woodgett, J., Cohen, P., and Smith, A. (2008). The ground state of embryonic stem cell self-renewal. Nature *453*, 519–523.

Supplemental Information

Systematic Identification of Culture Conditions for Induction and Maintenance of Naive Human Pluripotency

Thorold W. Theunissen[1,*], Benjamin E. Powell[1,*], Haoyi Wang[1,*], Maya Mitalipova[1], Dina A. Faddah[1,2], Jessica Reddy[1,2], Zi Peng Fan[1,3], Dorothea Maetzel[1], Kibibi Ganz[1], Linyu Shi[1], Tenzin Lungjangwa[1], Sumeth Imsoonthornruksa[1], Yonatan Stelzer[1], Sudharshan Rangarajan[1], Ana D'Alessio[1], Jianming Zhang[4], Qing Gao[1], Meelad M. Dawlaty[1], Richard A. Young[1,2], Nathanael Gray[4] and Rudolf Jaenisch[1,2,#]

Contents:

I. Extended Experimental Procedures

II. Supplemental Figure and Table Legends

III. Supplemental Figures

IV. Supplemental References

I. Extended Experimental Procedures

Gene targeting

Human ESCs and iPSCs were cultured in ROCK inhibitor Y-27632 (Stemgent, 10 μM) 24 hours prior to electroporation. Cell were harvested using 0.25% trypsin/EDTA solution (Invitrogen) and 1 x 10^7 cells resuspended in phosphate buffered saline (PBS) were electroporated with 40 μg of donor plasmids and 5 μg of each TALEN-encoding plasmid (Gene Pulser Xcell System, Bio-Rad: 250 V, 500μ F, 0.4 cm cuvettes (Costa et al., 2007)). Cells were subsequently plated on MEF feeder layers (DR4 MEFs for puromycin selection) in human ESC medium supplemented with ROCK inhibitor for the

first 24 hours. Individual colonies were picked and expanded after puromycin selection (0.5 µg/ml) 10 to 14 days after electroporation. Correctly targeted clones were confirmed by southern blot (NdeI digested) and used for the removal of floxed PGK-puro cassette. Cells were harvested using 0.25% trypsin/EDTA solution (Invitrogen) and 1×10^7 cells resuspended in PBS were electroporated with pTurbo-Cre (40 µg; GenBank accession number AF334827) (Gene Pulser Xcell System, Bio-Rad; 250 V, 500 µF, 0.4 cm cuvettes). Cells were subsequently plated on MEF feeder layers at a low density in human ESC medium supplemented with ROCK inhibitor. Individual colonies were picked 10–14 d after electroporation. The excision of PGK-puro was confirmed by Southern blot analysis.

Lentiviral infection

VSVG coated lentiviruses were generated in HEK-293 cells as described previously (Brambrink et al., 2008; Soldner et al., 2009; Soldner et al., 2011). Briefly, culture medium was changed 12 hr post-transfection and virus-containing supernatant was collected 48 to 72 hr post-transfection. Viral supernatant was filtered through a 0.45 mm filter. Virus-containing supernatants of the 2 reprogramming viruses (FUW-tetO-lox-hKLF2 and FUW-tetO-lox-hNANOG) were pooled and supplemented with the FUW-lox-M2rtTA virus and an equal volume of fresh culture medium. 1×10^6 human ESCs were seeded 24 hr before transduction in T75 flasks on matrigel in mTesr1 medium (STEMCELL Technologies). Two consecutive infections in the presence of 2 mg/ml of polybrene were performed over a period of 24 hr. Culture medium was changed 12 hr after the last infection. Five days after transduction, human ESCs were passaged using 0.25% trypsin/EDTA solution (Invitrogen) and re-plated on mitomycin C inactivated mouse embryonic fibroblast (MEF) feeder layers in conventional human ESC medium. To induce conversion to the naive state, human ESCs were trypsinized and seeded at a density of 1×10^5 cells per individual 6 well on a MEF feeder layer in the presence of ROCK inhibitor Y-27632. Medium was replaced 24 hr later with N2B27 basal medium supplemented with PD0325901 (Stemgent, 1 µM), CHIR99021 (Stemgent, 3 µM), 20 ng/mL hLIF (2i/L) and doxycycline (DOX) (Sigma-Aldrich; 2 µg/ml). OCT4-ΔPE-GFP+ human ESC colonies were picked manually within 10 days after DOX induction and

passaged using Accutase (Gibco) on a MEF feeder layer. Upon the addition of 2i/L/DOX, latent OCT4-ΔPE-GFP-negative cells could be removed almost entirely by additional treatment with 0.1 μM PD173074 and 5 μM SB431542, which effectively inhibit the signaling pathways on which primed human ESCs are reliant. These additional inhibitors also facilitated the isolation of transgene-dependent naive human ESCs from wild-type WIBR3 human ESCs.

Chemical screening

To screen for small molecules that support naive human pluripotency, doxycycline (DOX) was withdrawn from a clonal line of WIBR3 OCT4-ΔPE-GFP+ human ESCs derived in 2i/L/DOX. 24h after DOX withdrawal, cells were dissociated in 0.25% trypsin/EDTA solution (Invitrogen) and seeded at a density of 5000 cells per individual well in 96 well plates on a MEF feeder layer in 2i/L supplemented with the ROCK inhibitor Y-27632 (Stemgent, 10 μM). After an additional 24h, 2i/L medium in each individual 96 well was supplemented with a kinase inhibitor from the LINCS inhibitor library (Gray Laboratory, Dana Farber Cancer Institute, Boston, MA) at a final concentration of 1 μM. To improve experimental consistency the small molecule library was applied using the Caliper RapidPlate 96 well Liquid Handling System (Zymark, Westborough, MA). Briefly, a master plate containing 10 mM stock solution of the library in DMSO was first diluted to 100 μM in HEPES aqueous solution (daughter plate), and subsequently to 10 μM in N2B27 basal medium supplemented with 2i/L (granddaughter plate). This granddaughter plate was then diluted a further 10X in 2i/L medium, the final medium was pre-mixed and applied slowly to the 96 well assay plate. Following two medium changes during a seven day period the proportion of OCT4-ΔPE-GFP+ human ESCs in each well was assessed using the High-Throughput System on the LSRFortessa SORP (Beckton-Dickinson, San Jose, CA). To screen for small molecules that improve the proportion of viable OCT4-ΔPE-GFP+ cells, this assay was modified by the addition of 1.0 μM SB590885 to each well and inclusion of a 10 minute DAPI (Life Technologies) staining prior to high-throughput FACS analysis. Viable cells were gated from the DAPI-negative fraction and subsequently assessed for GFP status.

Flow cytometry

To assess the proportion of OCT4-ΔPE-GFP+ human ESCs, a single cell suspension was filtered, stained with DAPI (Life Technologies), and assessed on the LSR II SORP (Beckton-Dickinson, San Jose, CA) or LSRFortessa SORP (Beckton-Dickinson, San Jose, CA). On the LSR II SORP, mTomato (for detection of autofluorescence) was excited by a Coherent Compass 561 nm (25 mW) yellow/green laser and detected using a bandpass filter (emission) of 610/20, GFP was excited by a Coherent Sapphire Solid State 488 nm (100 mW) blue laser and detected using a bandpass filter (emission) of 525/50, and DAPI was excited by a Lightwave Xcyte 355 nm (60 mW) UV laser and detected using a bandpass filter (emission) of 450/50. On the LSRFortessa SORP, PE-Texas Red (for detection of autofluorescence) was excited by a Coherent Compass 561 nm (50 mW) yellow/green laser and detected using a bandpass filter (emission) of 610/20, GFP was excited by a Coherent Sapphire Solid State 488 nm (100 mW) blue laser and detected using a bandpass filter (emission) of 530/30, and DAPI was excited by a Lightwave Xcyte 355 nm (60 mW) UV laser and detected using a bandpass filter (emission) of 450/50.

Immunostaining

Immunostaining was performed according to standard protocols using the following primary antibodies: Oct-3/4 (mouse monoclonal, Santa Cruz Biotechnology); hNANOG (Cat. No. AF1997, goat polyclonal, R&D Systems); AFP (Cat. No. A8452, mouse monoclonal, Sigma); HNF4a (goat polyclonal, Santa Cruz); Nestin (Cat. No. AB5922, mouse monoclonal, Milipore); Pax6 (Cat. No. PRB-278P, rabbit polyclonal, Covance). Appropriate Alexa Fluor dye conjugated secondary antibodies (Invitrogen) were used. Nuclei were stained with DAPI (Life Technologies). Images were taken using LSM710 confocal microscope (Zeiss) or Inverted microscope (Eclipse Ti-Nikon).

Human ESC derivation

Human embryos at 8-cell or blastocyst stage produced by in vitro fertilization for clinical

purposes were obtained with informed written consent and approved by MIT Institutional review board. Embryos were thawed and cultured in Multiblast medium (Irvine Scientific) supplemented with 20% serum protein substitute (50 mg/ml Quinn's Advantage SPS, InVitro Fertilization) until day 6 in 5% O_2, 5% CO_2 and 90% N_2 gas mixture. Embryos were plated as a whole blastocyst after removal of the zona pellucida by brief incubation in protease solution (Sigma), and further cultured in optimized medium for naive human pluripotency on a MEF feeder layer. At day 6-12 after plating, inner cell mass outgrowths were passaged by brief incubation in Accutase solution and dispersed to clumps of 5-10 cells. Freshly derived naive human ESCs proliferated slowly and were passaged by Accutase to single cells and frozen and used for further analysis. Karyotype analysis was performed by the Cytogenetics Laboratory at Tufts Medical Center in Boston, MA.

Generation of naive human iPSCs

Primary C1 *OCT4-ΔPE-GFP* human iPSCs harboring proviral integrations of OCT4, SOX2 and KLF4 were differentiated into fibroblast-like cells as described previously (Xu et al., 2004). iPSCs were differentiated by embryoid body formation in fibroblast medium for 5 days and subsequently plated onto adherent tissue culture dishes and passaged according to primary fibroblast protocols using trypsin for at least four passages before the start of experiments. For the derivation of naive secondary iPSCs, secondary fibroblasts were plated at 1×10^4 per 35 mm on MEF feeder layers. Twenty four hours later, fibroblast medium was replaced by primed human ESC medium supplemented with DOX (Sigma-Aldrich; 2 μg/ml) for six days. After six days the medium was replaced with naive 5i/L/A medium supplemented with FGF2 and DOX. After day 10 post DOX induction, GFP positive naive human iPSC colonies could be readily isolated and clonally expanded in 5i/L/A medium in the absence of doxycycline.

For primary reprogramming experiments fibroblasts were seeded on gelatin at a density of 1×10^5 cells per 35 mm. Viral supernatants of tetO-hSTEMCCA-loxP and FUW-lox-M2rtTA virus were used for transduction at a 3:1 ratio. Two consecutive infections in the presence of 2 mg/ml of polybrene were performed over a period of 24 hr. Culture

medium was changed 12 hr after the last infection. 24 hours later transduced fibroblasts were trypsinized and plated at 1×10^4 per 35 mm on MEF feeder layers. Twenty four hours later, fibroblast medium was replaced by primed human ESC medium supplemented with DOX (Sigma-Aldrich; 2 μg/ml) for six days. After six days the medium was replaced with naive 5i/L/A medium supplemented with FGF2 and DOX. After day 10 post DOX naive human iPSC colonies could be readily isolated and clonally expanded in 5i/L/A medium in the absence of DOX.

RNA FISH and imaging

We performed RNA FISH as outlined previously (Faddah et al., 2013; Raj et al., 2010; Raj et al., 2008). All hybridizations were performed in solution using probes coupled to either tetramethylrhodamine (TMR) (Invitrogen), Alexa 594 (Invitrogen) or Cy5 (GE Amersham). We used TMR for the probes against human KLF4 mRNA, Alexa 594 for the probes against human OCT4, REX1, and NANOG mRNA, and Cy5 for the probes against human OCT4 mRNA. Optimal probe concentrations during hybridization were determined empirically. Imaging involved taking stacks of images spaced 0.4 μm apart using filters appropriate for DAPI, TMR, Alexa 594 and Cy5. All images were taken with a Nikon Ti-E inverted fluorescence microscope equipped with a 100X oil-immersion objective and a Photometrics Pixis 1024 CCD camera using MetaMorph software (Molecular Devices, Downington, PA). During imaging, we minimized photobleaching through the use of an oxygen-scavenging solution using glucose oxidase. We segmented the cells manually and counted the number of fluorescent spots, each of which corresponds to an individual mRNA, using a combination of a semi-automated method described in (Itzkovitz et al., 2011; Raj et al., 2008) and custom software written in MATLAB (Mathworks).

RNA Extraction and Synthetic RNA Spike-In

Total RNA and sample preparation for microarray analysis was performed as previously described (Loven et al., 2012). Briefly, 1 million naive or primed human ESCs were trypsinized and purified from GFP-labeled MEFs using the FACSAria (Beckton-

Dickinson) prior to lysis and RNA extraction. Biological duplicates were subsequently collected and homogenized in 1 ml of TRIzol Reagent (Life Technologies, 15596-026), purified using the mirVANA miRNA isolation kit (Ambion, AM1560) following the manufacturer's instructions and re-suspended in 100 ml nuclease-free water (Ambion, AM9938). Total RNA was spiked-in with ERCC RNA Spike-In Mix (Ambion, 4456740), treated with DNA-freeTM DNase I (Ambion, AM1906) and analyzed on Agilent 2100 Bioanalyzer for integrity. RNA with the RNA Integrity Number (RIN) above 9.8 was hybridized to GeneChip PrimeView Human Gene Expression Arrays (Affymetrix).

Microarray Sample Preparation and Analysis

100 ng of total RNA was used to prepare biotinylated cRNA (cRNA) according to the manufacturer's protocol (30 IVT Express Kit, Affymetrix 901228). GeneChip arrays (Primeview, Affymetrix 901837) were hybridized and scanned according to standard Affymetrix protocols. The raw data was obtained by using Affymetrix Gene Chip Operating Software using default settings. A Primeview CDF provided by Affymetrix was used to generate .CEL files. The CEL files were processed with the expresso command to convert the raw probe intensities to probeset expression values with MAS5 normalization using the standard tools available within the affy package in R. The probesets of the same gene were next collapsed into a single value to represent the gene by taking the mean value. Differential gene expression was determined using moderated t-statistic in the "limma" package (http://bioinf.wehi.edu.au/limma/) from Bioconductor (www.bioconductor.org) (Smyth, 2004). A gene was considered differentially expressed if it met the following criteria: 1) absolute log2 fold- change ≥1 between the mean expression of the two conditions, 2) adjusted p-value less than 0.1 by a moderated t-test within the limma package with BH multiple hypothesis testing correction.

We also processed expression profiles of naive human ESCs and primed human ESCs published in previous studies (Chan et al., 2013; Gafni et al., 2013; Ware et al., 2014). For the expression profiles from the naive human ESCs (GSM1139484 and

GSM1139494) and primed human ESCs (GSM1139488 and GSM1139495) published in Gafni et al., 2013, the Affymetric CDF file (version V1.r3) was used to generate .CEL files. The CEL files were processed with RMA normalization using the standard tools variable within the affy package in R. For the expression profiles from the naive human ESCs (ERR361240, ERR361242, ERR361244) and primed human ESCs (ERR361241, ERR361243, ERR361245) published in Chan et al., 2013, RNA-seq reads were aligned using the software Bowtie (Langmead et al., 2009) to NCBI build 37 (hg19) of the mouse genome with the settings: -e 70 -k 1 -m 1 - n 2. The RPKM (reads per kilobase per million) for each RefSeq gene was calculated using RPKM_count.py (v2.3.5) counting only exonic reads (-e option). For the expression profiles from the naive human ESCs and primed human ESCs published in Ware et al., 2014, the processed expression tables were downloaded from the Sage Synapse Commons Repository: https://www.synapse.org/#!Synapse:syn1447088. The probesets of the same gene were next collapsed into a single value to represent the gene by taking the mean value. The p-values for differential gene expression analysis between the naive and primed human ESCs from each study were determined using moderated t-statistic in the "limma" package (http://bioinf.wehi.edu.au/limma/) from Bioconductor (www.bioconductor.org) (Smyth, 2004). The log2 fold changes between the naive and primed human ESCs and p-values for all Refseq genes from each study were displayed in Volcano plots.

To determine gene ontology (GO) categories, up-regulated (> 3 fold) and down-regulated (> 3 fold) gene lists were subjected to DAVID bioinformatics tool (http://david.abcc.ncifcrf.gov/). Categories were subsequently sorted according to Benjamini multiple testing correction (p-value < 0.01).

To assess X-linked gene expression in female human ESCs in conventional (primed) and naive medium, the expression of individual genes was compared with the average of three male control human iPSC samples previously analyzed on the same platform (GSM1142930, GSM1142931, GSM1142932). Moving average plots were generated as described previously (Bruck and Benvenisty, 2011).

Cross-Species Gene Expression Analysis

Cross-species gene expression analysis was performed as previously described (Gafni et al., 2013) with some modifications. Human ESC array data was compared to previously published naive mouse ESC and primed mouse EpiSC gene expression datasets on an Agilent 4 X 44k array platform (GSE15603). Probeset mapping between the Agilent 4 X 44k array platform and Affymetric Primeview platform of human-mouse homologous genes was downloaded from Ensembl biomart (www.ensembl.org/biomart). The probesets of the same gene in human or mouse were next collapsed into a single value to represent the gene independently in each species by taking the mean value. The relative expression values from mouse and human were next calculated independently by dividing the expression values of the samples by the mean of expression values within the same genes across samples in the same species. Pair-wise comparisons were performed on the relative expression values from the human and mouse expression profiles using Pearson correlation coefficients (PCCs). The average linkage hierarchical clustering of the Pearson correlation was shown in the heatmap.

Chromatin Immunoprecipitation (ChIP)

Cells were cross-linked for 10 minutes at room temperature by the addition of one-tenth of the volume of 11% formaldehyde solution (11% formaldehyde, 50mM HEPES pH 7.3, 100 mM NaCl, 1 mM EDTA pH 8.0, 0.5 mM EGTA pH 8.0) to the growth media followed by quenching with 100 mM glycine. Cells were washed twice with PBS, then the supernatant was aspirated and the cell pellet was flash frozen in liquid nitrogen. Frozen cross-linked cells were stored at -80C. 20 ul of Dynal magnetic beads (Sigma) were blocked with 0.5% BSA (w/v) in PBS. Magnetic beads were bound with 2 ug of the indicated antibody. The antibodies used were as follows: H3K4me3 (Abcam ab8580) and H3K27me3 (Millipore 07-449). Cross-linked cells were lysed with lysis buffer 1 (50 mM HEPES pH 7.3, 140 mM NaCl, 1mM EDTA, 10% glycerol, 0.5% NP-40, and 0.25% Triton X-100) and resuspended and sonicated in sonication buffer (50 mM Tris-HCl [pH 7.5], 140 mM NaCl, 1 mM EDTA, 1% Triton X-100, 0.1% Na-deoxycholate, 0.1% SDS).

Cells were sonicated at 4°C with a Bioruptor (Diagenode) at high power for 25 cycles for 30s with 30s between cycles. Sonicated lysates were cleared and incubated overnight at 4°C with magnetic beads bound with antibody to enrich for DNA fragments bound by the indicated factor. Beads were washed two times with sonication buffer, one time with sonication buffer with 500 mM NaCl, one time with LiCl wash buffer (20 mM Tris pH 8.0, 1 mM EDTA, 250 mM LiCl, 0.5% NP-40, 0.5% Na-deoxycholate) and one time with TE with 50 mM NaCl. DNA was eluted in elution buffer (50 mM Tris-HCL pH 8.0, 10 mM EDTA, 1% SDS). Cross-links were reversed overnight. RNA and protein were digested using RNase A and Proteinase K, respectively and DNA was purified with phenol chloroform extraction and ethanol precipitation.

Illumina Sequencing and Library Generation

Purified ChIP DNA was used to prepare Illumina multiplexed sequencing libraries. Libraries for Illumina sequencing were prepared following the Illumina TruSeq DNA Sample Preparation v2 kit protocol with the following exceptions. After end-repair and A-tailing, Immunoprecipitated DNA (10-50 ng) or Whole Cell Extract DNA (50 ng) was ligated to a 1:50 dilution of Illumina Adaptor Oligo Mix assigning one of 24 unique indexes in the kit to each sample. Following ligation, libraries were amplified by 18 cycles of PCR using the HiFi NGS Library Amplification kit from KAPA Biosystems. Amplified libraries were then size-selected using a 2% gel cassette in the Pippin Prep system from Sage Science set to capture fragments between 200 and 400 bp. Libraries were quantified by qPCR using the KAPA Biosystems Illumina Library Quantification kit according to kit protocols. Libraries with distinct TruSeq indexes were multiplexed by mixing at equimolar ratios and running together in a lane on the Illumina HiSeq 2000 for 40 bases in single read mode.

Gene Sets and Annotations

All analyses were performed using RefSeq (NCBI37/HG19) (Pruitt et al., 2007) human gene annotations.

ChIP-Seq Data Processing

All ChIP-Seq datasets were aligned using Bowtie (version 0.12.9) (Langmead et al., 2009) to build version NCBI37/HG19 of the human genome using –n2, -e70, -m2, -k2, --best. We used the MACS version 1.4.1 (Model based analysis of ChIP-Seq) (Zhang et al., 2008) peak finding algorithm to identify regions of ChIP-Seq enrichment over background. A p-value threshold of enrichment of 1e-9 was used for all datasets.

Heatmap representation of read density profiles.

A gene was defined as Polycomb-associated if an enriched region for H3K27me3 (representing polycomb complexes) was located within +/- 1 kb of the TSS. H3K27me3 is a histone modification associated with Polycomb complexes (Boyer et al., 2006). The annotated TSS of Polycomb-associated genes were aligned at the center in the composite view of signal density profile. The average ChIP-seq read density (r.p.m./bp) around 5 kb centered on the centers in 50 bp bin was calculated.

Meta Representations of ChIP-Seq Occupancy

Genome-wide average ‘‘meta’’ representations of ChIP-seq occupancy of different factors were created by mapping ChIP-seq read density to Polycomb-associated genes. We created three sets of regions: upstream, gene body and downstream. 80 equally-sized bins divided the -2000 to 0 promoter region, 200 equally-sized bins divided the length of the gene body, and 80 equally-sized bins divided the 0 to +2kb downstream region. The average ChIP-Seq factor density in each bin was calculated to create a meta genome-wide average in units of rpm/bp.

Differentiation assays

Teratoma formation

Single cell dissociations of naive human ESCs were resuspended in 250 μl of medium and co-injected subcutaneously with 250 ul of matrigel in the flank of NOD/SCID mice. Tumors generally developed within 8 to 12 weeks and animals were sacrificed before tumor size exceeded 3 cm in diameter. Teratomas were isolated after sacrificing the mice and fixed in formalin. After sectioning, teratomas were diagnosed based on

hematoxylin and eosin staining. All animal experiments were performed in compliance with protocol # 1031-088-16 from the Committee on Animal Care at MIT.

Directed differentiation into hepatocytes

Differentiation of naive human ESCs into hepatocytes was obtained as described previously in conventional human ESCs (Si-Tayeb et al., 2010). Single cells were cultivated on Matrigel coated plates (2 mg/ml) under low oxygen conditions. Differentiation was initiated by cultivating cells for 5 days in Activin A (100 ng/ml) containing RPMI/B27 medium under ambient oxygen, followed by 5 days in BMP4 (20 ng/ml)/ FGF-2 (10 ng/ml) containing RPMI/B27 and 5 days in HGF (20 ng/ml) containing RPMI/B27 under 5% oxygen. Finally cells were cultured for 5 days in Hepatocyte Culture Medium supplemented with Oncostatin-M (20 ng/ml) under ambient oxygen conditions. Generated hepatocytes were identified by expression of AFP and HNF4a.

Morula and blastocyst injection

Six to eight weeks old B6D2F1 females were superovulated with 7.5 I.U of Pregnant Mare Serum (PMS) each given by intraperitoneal (IP) injections followed by an IP injection of 7.5 I.U of Human Chorionic Gonadotropin (HCG) 46 to 48 hours later. They were then mated with B6D2F1 stud males and checked for copulatory plugs the following day. One-cell fertilized embryos were harvested and incubated at 37°C, 5% O_2 for 2-3 days in KSOM medium (Zenith Biotech). At the 8-cell, morula, and blastocyst stage, embryos were injected with 10-15 naive human ESCs using a 16 μm piezo needle (Humagen). During injection, the human cells were kept continuously in drops of their own culture medium, whereas the embryos were kept in M2+ROCKi Y-27632 (Stemgent, 10 μM) drops. After injection, the injected embryos were cultured in KSOM+ROCKi for 3-4 hours, then washed in 5 individual drops of KSOM and cultured overnight to the blastocyst stage. When the injected embryos reached the blastocyst stage, 20 embryos were transferred into each E2.5 p.c pseudopregnant female by uterine transfer. Seven to eight days later, the post-op females were sacrificed by CO_2

asphyxiation and embryos were harvested at E9.5-E10.5 p.c for analysis. All animal experiments were performed in compliance with protocol # 1031-088-16 from the Committee on Animal Care at MIT. In addition, interspecies chimerism experiments were approved by the Embryonic Stem Cell Research Oversight *(*ESCRO*)* Committee at Whitehead Institute.

qRT-PCR

Total RNA was isolated using the Rneasy Kit (QIAGEN) and reversed transcribed using the Superscript III First Strand Synthesis kit (Invitrogen). Quantitative RT-PCR analysis was performed in triplicate using the ABI 7900 HT system with FAST SYBR Green Master Mix (Applied Biosystems). Gene expression was normalized to GAPDH. Error bars represent the standard deviation (SD) of the mean of triplicate reactions. Primer sequences are included in the table below.

Primers used in this study

Gene	Primer sequence (5'- 3')	Application
NANOG-F	GCAGAAGGCCTCAGCACCTA	RT-PCR
NANOG-R	AGGTTCCCAGTCGGGTTCA	
OCT4-F	GCTCGAGAAGGATGTGGTCC	RT-PCR
OCT4-R	CGTTGTGCATAGTCGCTGCT	
SOX2-F	CACTGCCCCTCTCACACATG	RT-PCR
SOX2-R	TCCCATTTCCCTCGTTTTTCT	
STELLA-F	GTTACTGGGCGGAGTTCGTA	RT-PCR
STELLA-R	TGAAGTGGCTTGGTGTCTTG	
KLF4-F	GATGGGGTCTGTGACTGGAT	RT-PCR
KLF4-R	CCCCCAACTCACGGATATAA	
GAPDH-F	CGAGATCCCTCCAAAATCAA	RT-PCR
GAPDH-R	ATCCACAGTCTTCTGGGTGG	

REX1-F	GGAATGTGGGAAAGCGTTCGT	RT-PCR
REX1-R	CCGTGTGGATGCGCACGT	
PRDM14-F	TGAGCCTTCAGGTCACAGAG	RT-PCR
PRDM14-R	ATTTCCTATCGCCCTTGTCC	
GFP-F	AGAACGGCATCAAGGTGAAC	RT-PCR
GFP-R	TGCTCAGGTAGTGGTTGTCG	
FUW-KLF2-F	GATTTTGCTGGGTTGGTTTTT	RT-PCR
FUW-KLF2-R	CCACATAGCGTAAAAGGAGCA	
FUW-NANOG-F	GCTGGGGAAGGCCTTAATGT	RT-PCR
FUW-NANOG-R	CCACATAGCGTAAAAGGAGCA	
PAX6-F	CTTTGCTTGGGAAATCCGAG	RT-PCR
PAX6-R	AGCCAGGTTGCGAAGAACTC	

II. Supplemental Figure and Table Legends

Figure S1. A reporter system for naive human pluripotency based on endogenous *OCT4* distal enhancer activity [associated with Figure 1]

(**A**) Southern blot analysis confirming deletion of the PE in *OCT4-2A-GFP* human ESCs and the removal of floxed PGK-puro cassette. NdeI-digested genomic DNA was hybridized with 5’ and 3’ external probes. Expected fragment size: WT (wild type) = 5.6kb, T (targeted) = 6.4 kb, ∆PE (targeted allele after PGK-puro removal) = 4.6kb. Note that some clones obtained after Cre excision exhibited deletion of the entire *OCT4-2A-GFP* sequence (*OCT4* KO allele) due to the presence of an additional loxP site from prior round of *OCT4-2A-GFP* targeting (Hockemeyer et al., 2011).

(**B**) Phase and GFP images of OCT4-∆PE-GFP+ cells obtained after DOX induction of lentiviral KLF2+NANOG. Following primary infection, WIBR3 human ESCs containing the *OCT4-∆PE-GFP* reporter allele were trypsinized and treated with hESM, 2i/L or 2i/L/DOX for one week. 40X magnification.

(**C**) Immunofluorescence staining for NANOG in a clonal line of OCT4-∆PE-GFP-positive cells derived in 2i/L/DOX, and a clonal line of OCT4-∆PE-GFP-negative primitive neural stem cells (pNSCs) derived in 2i/L. 100X magnification.

(**D**) Quantitative gene expression analysis for GFP, SOX2, PRMD14 and PAX6 in clonal OCT4-∆PE-GFP+ human ESC lines generated in 2i/L/DOX, secondary primed cells generated by withdrawal of DOX and expansion in conventional hESM, and clonal lines of OCT4-∆PE-GFP-negative pNSCs derived in 2i/L. Error bars indicate ±1 SD of technical replicates.

Figure S2. Identification of small molecules that maintain OCT4-∆PE-GFP activity after transgene withdrawal [associated with Figure 2]

(**A**) Raw data obtained from high-throughput flow cytometric analysis of the proportion of OCT4-∆PE-GFP+ cells in 96 wells supplemented with a kinase inhibitor library (n=2).

(**B**) Quantitative gene expression analysis for STELLA, KLF4, PRDM14 and SOX2 in a clonal line of OCT4-∆PE-GFP+ cells maintained in 2i/L/DOX or for five passages without DOX in the presence of each candidate compound. Error bars indicate ±1 SD of technical replicates.

(**C**) Flow cytometric analyses of the proportion of viable (DAPI-negative) and OCT4-ΔPE-GFP+ cells in 2i/L/DOX or two passages after DOX withdrawal in 2i/L/SB590885 (1 µM).

Figure S3. Optimization of medium for maintaining viable OCT4-ΔPE-GFP+ cells [associated with Figure 3]

(**A**) Raw data obtained from high-throughput flow cytometric analysis of the proportion of DAPI-/OCT4-ΔPE-GFP+ cells in 96 wells supplemented with three plates of a kinase inhibitor library in the presence of the primary hit compound SB590885 (1 µM) (n=2). Asterisks denote autofluorescent compounds.

(**B**) Hit compounds from viability screen using a clonal line of WIBR3 OCT4-ΔPE-GFP+ ESCs established in 2i/L/DOX. Asterisks denote autofluorescent compounds.

(**C**) Phase images and flow cytometric analyses of OCT4-ΔPE-GFP+ cells maintained in 2i/L/SB590885 (0.5 µM) ± ROCK inhibitor Y-27632 (10 µM) for two passages. CHIR99021 was applied at the optimized concentration of 0.3 µM. 40X magnification.

(**D**) Phase and GFP images of OCT4-ΔPE-GFP+ cells maintained in 2i/L/DOX, 2i/L/SB590885 (0.5 µM) + ROCK inhibitor Y-27632 (10 µM) or the same medium in which CHIR99021 (0.3 µM) was replaced with an alternative GSK3 inhibitor, IM-12 (1.0 µM). 40X magnification.

Figure S4. Direct conversion of conventional human ESCs to naive pluripotency in 5i/L [associated with Figure 4]

(**A**) Karyotype analysis of naive human ESCs WIBR2 (P8 in 5i/L/A) indicates a normal diploid chromosome content. Cytogenetic analysis was performed on 20 metaphase cells.

(**B**) Karyotype analysis of naive human ESCs WIN1 (P7 in 5i/L/FA) indicates a normal diploid chromosome content. Cytogenetic analysis was performed on 20 metaphase cells.

(**C**) (Top) Strategy for generating naive human iPSCs from normal and patient-derived disease-specific primary fibroblasts through primary infection of OCT4, SOX2, KLF4 and MYC. (Bottom) Phase images of clonal naive iPSC lines reprogrammed from control and patient fibroblasts. 40X magnification.

(**D**) Phase images of OCT4-ΔPE-GFP+ cells three passages after withdrawal of individual inhibitors and growth factors. 40X magnification.

(**E**) Quantitative gene expression analysis for OCT4, SOX2, KLF2 and REX1 three passages after withdrawal of individual inhibitors and growth factors. Error bars indicate ±1 SD of technical replicates.

Figure S5. Evaluation of alternative culture conditions for naive human pluripotency. [associated with Figure 5]

(**A**) Phase and fluorescence images (Top) and flow cytometric analysis of the proportion of OCT4-ΔPE-GFP+ cells (Bottom) in OCT4-ΔPE-GFP+ cells derived in 5i/L/FA and maintained for three passages in the presence of additional media additives, as indicated. Where indicated recombinant human insulin (Sigma) was added at 12.5 μg/mL final concentration. 40X magnification.

(**B**) Quantitative gene expression analysis for KLF2 and KLF4 in OCT4-ΔPE-GFP-positive naive human ESCs cultured in 5i/L/A and supplemented with various components of the medium of Gafni et al. (2013). Error bars indicate ±1 SD of technical replicates.

(**C**) Phase and fluorescence images (Top) and flow cytometric analysis of the proportion of OCT4-ΔPE-GFP+ cells (Bottom) in OCT4-ΔPE-GFP+ cells derived in 5i/L/FA and maintained for three passages in 20% KSR basal medium supplemented with the media additives described in (A). 40X magnification.

Figure S6. Transcriptional profiling of naive human ESCs in 5i/L/A and 6i/L/A [associated with Figure 6]

(**A**) Gene ontology (GO) analysis showing up- and down-regulated gene categories with most significant p values between our naive human conditions and primed human ESCs.

(B) Quantitative gene expression analysis for KLF4 and REX1 in human ESCs cultured in parallel in primed medium, 5i/L/FA and the medium of Gafni et al. (2013). Error bars indicate ±1 SD of technical replicates.

(**C**) Expression profile of transcripts upregulated in 6i/L/A during human embryonic development. For each gene, the normalized expression values in human ESCs cultured in 6i/L/A vs. primed human ESCs are indicated (Left). An unpaired two-tailed t test was performed to establish the degree of significance. Expression of the corresponding transcript is shown at nine stages of human pre-implantation development, as detected by single cell RNA-Seq

profiling (Yan et al., 2013) (Right). This comparison indicates that naive-associated transcripts upregulated in 6i/L/A are enriched at the morula/epiblast stage of human development when compared to human ESCs at passage 0 or passage 10.

Figure S7. Additional characterization of naive human ESCs in 5i/L/A [associated with Figure 6]

(A) Single molecule (sm) RNA FISH analysis using OCT4 and NANOG probes in WIBR2 human ESCs cultured in primed medium, the medium of Gafni et al. (2013) or 5i/L/A (P5). Scale bar indicates 5 μm.

(B) Variability in NANOG expression compared between single human ESCs cultured in primed medium, 5i/L/A or the medium of Gafni et al. (2013).

(**C**) Teratomas generated from WIBR3 OCT4-ΔPE-GFP-positive human ESCs derived and maintained in 5i/L±FA and WIBR3 AAVS1-tdTomato human ESCs in 5i/L/FA. Representative tissues of the three germ layers are indicated. 200X magnification.

(**D**) Immunofluorescence staining for AFP and HNF4a following 20d of hepatic differentiation in naive WIBR2 human ESCs derived and maintained in 5i/L/A. Scale bars indicate 100 μm.

(**E**) Table summarizing injections of human ESCs maintained in 5i/L/FA (Top) or the medium of Gafni et al. (2013) (Bottom) into mouse embryos. C1-AAVS1-GFP human ESCs in the medium of Gafni et al. (2013) were cultured on MEFs, gelatin/vitronectin or matrigel prior to injection. (*) E10.5 embryos injected with human ESCs cultured in 5i/L/FA and the medium of Gafni et al. (2013) were mixed during collection, but none were identified as positive. (**) 30 injected embryos were lost during transfer.

Table S1. Gene expression of naive and primed human ESCs [associated with Figure 6]

Normalized gene expression values from microarray samples shown in Figure 6A. Dataset includes two primed human ESC samples (hESM) and five naive human ESC samples (5i/L/A or 6i/L/A). Genes are ordered according to Column I (log_2FC naive vs. primed) with the most upregulated genes in the naive state at the top of the list.

III. Supplemental Figures

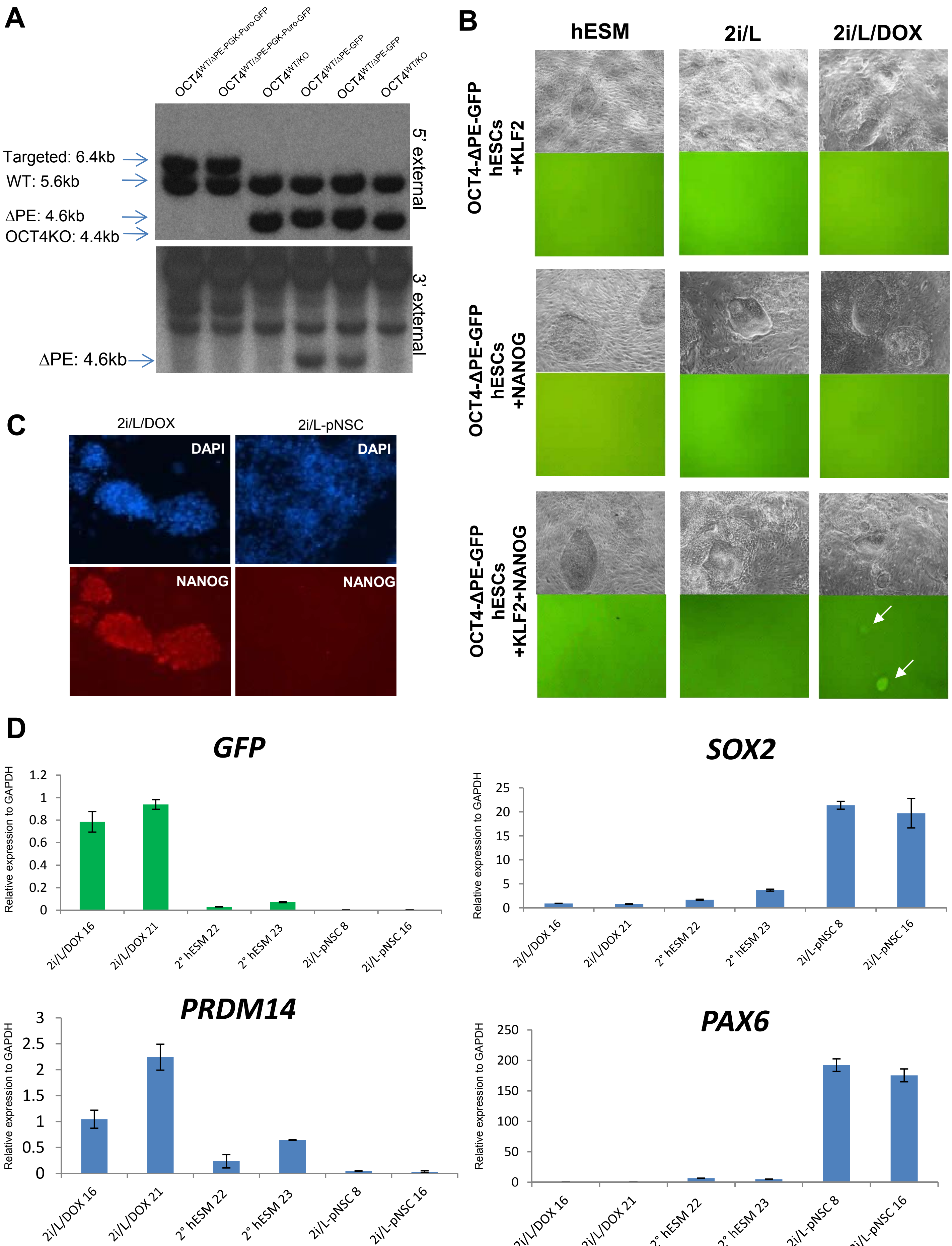
A
OCT4WT/ΔPE-PGK-Puro-GFP
OCT4WT/ΔPE-PGK-Puro-GFP
OCT4WT/KO
OCT4WT/ΔPE-GFP
OCT4WT/ΔPE-GFP
OCT4WT/KO
Targeted: 6.4kb
WT: 5.6kb
ΔPE: 4.6kb
OCT4KO: 4.4kb
5' external
3' external
ΔPE: 4.6kb
B
hESM
2i/L
2i/L/DOX
OCT4-ΔPE-GFP hESCs +KLF2
OCT4-ΔPE-GFP hESCs +NANOG
OCT4-ΔPE-GFP hESCs +KLF2+NANOG
C
2i/L/DOX
2i/L-pNSC
DAPI
DAPI
NANOG
NANOG
D
GFP
SOX2
PRDM14
PAX6
Relative expression to GAPDH
2i/L/DOX 16
2i/L/DOX 21
2° hESM 22
2° hESM 23
2i/L-pNSC 8
2i/L-pNSC 16

FIGURE S1

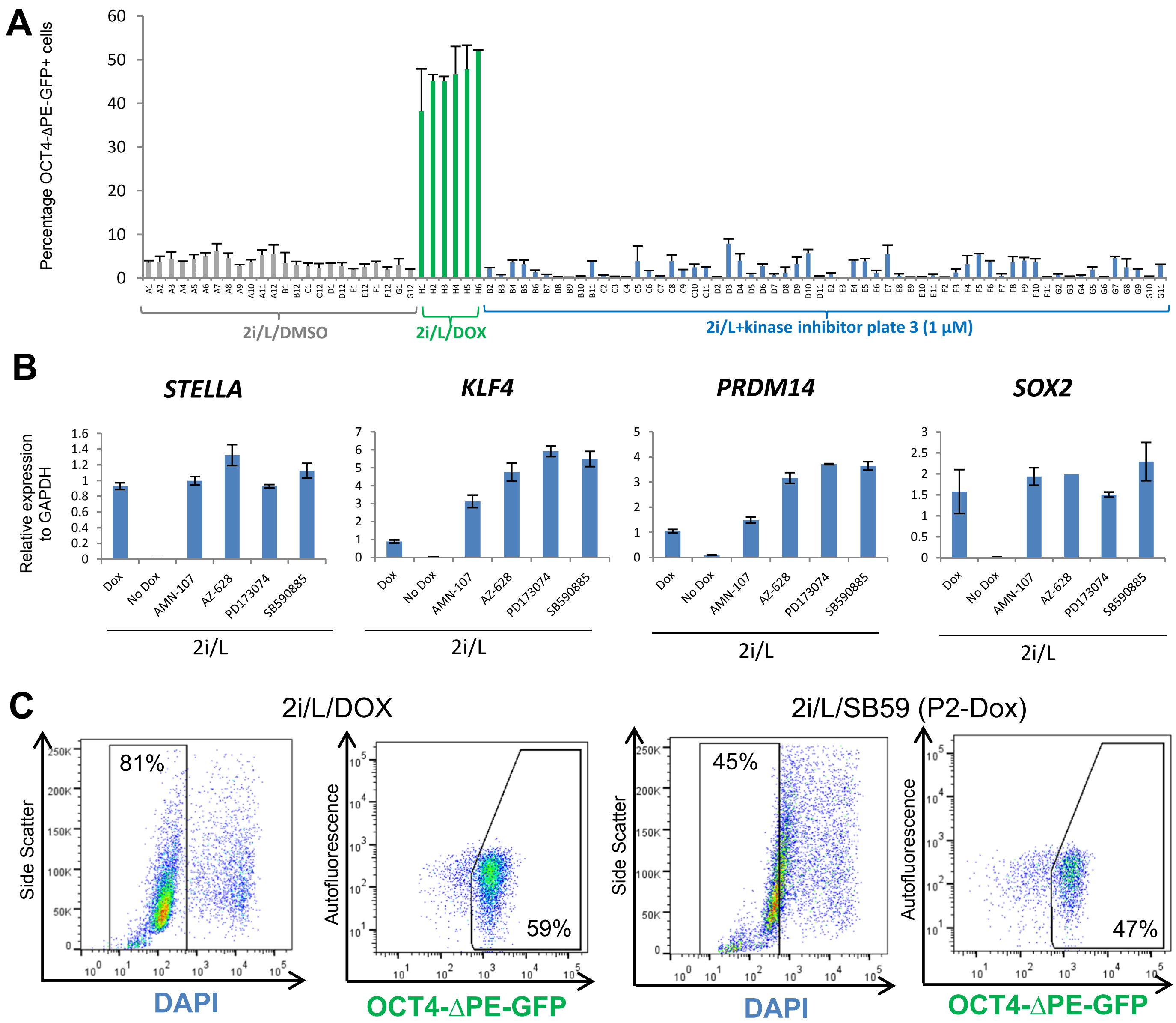
A
Percentage OCT4-ΔPE-GFP+ cells
2i/L/DMSO
2i/L/DOX
2i/L+kinase inhibitor plate 3 (1 μM)
B
STELLA
KLF4
PRDM14
SOX2
Relative expression to GAPDH
Dox
No Dox
AMN-107
AZ-628
PD173074
SB590885
2i/L
C
2i/L/DOX
2i/L/SB59 (P2-Dox)
Side Scatter
81%
59%
45%
47%
Autofluorescence
DAPI
OCT4-ΔPE-GFP

FIGURE S2

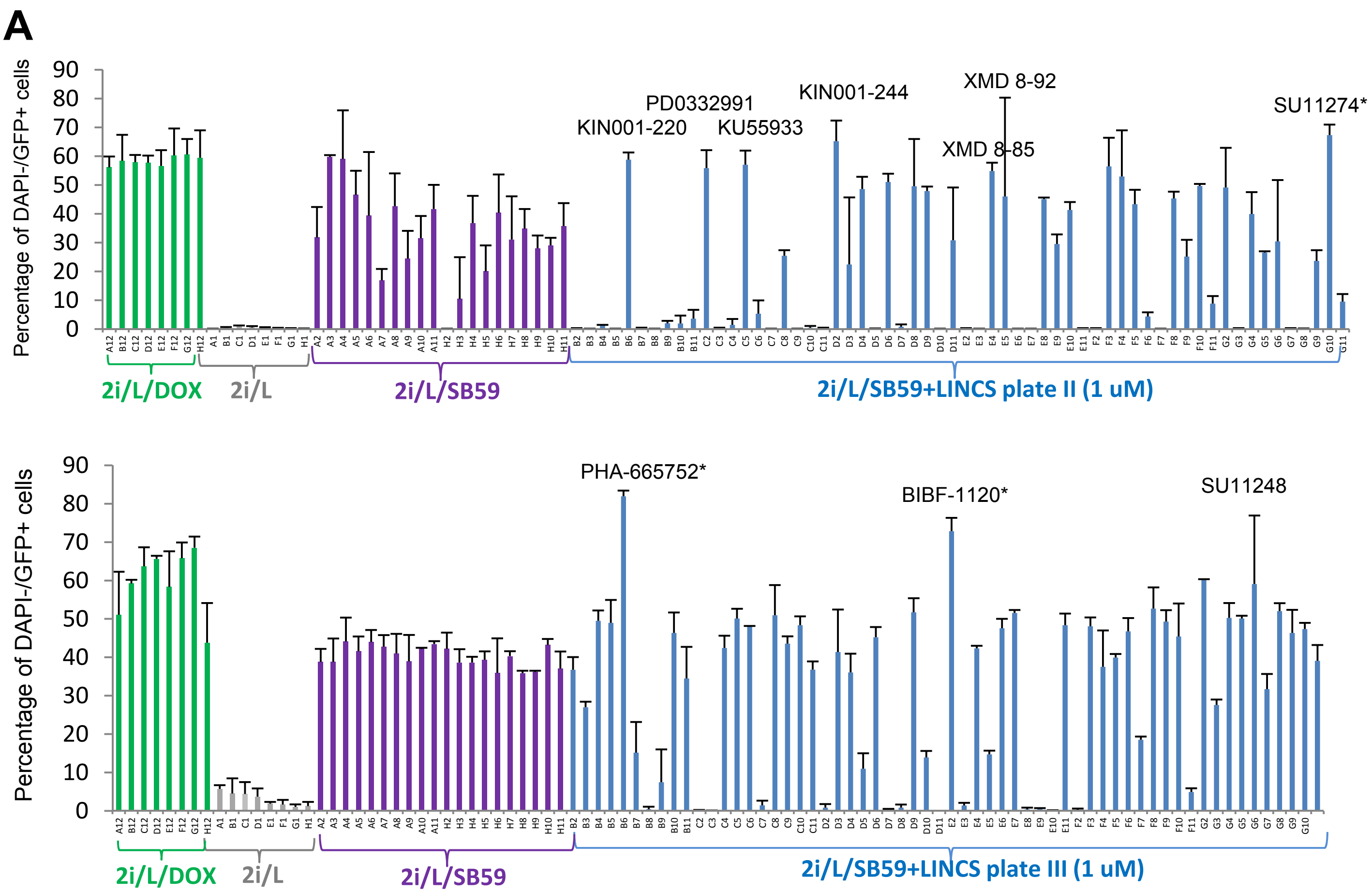

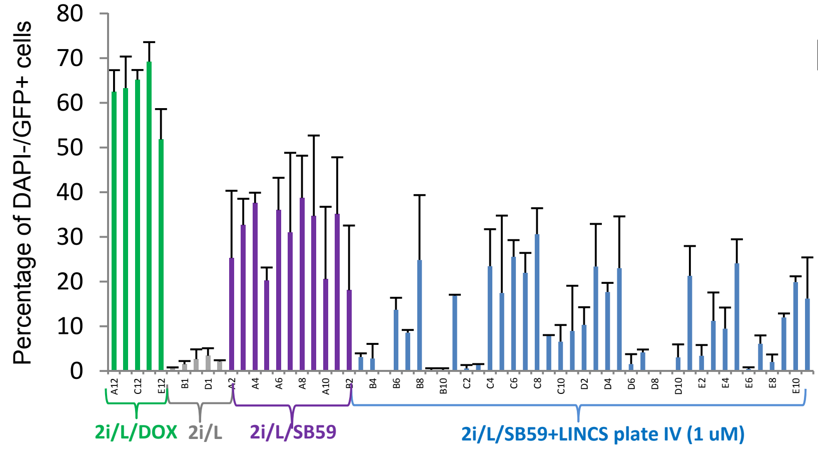

B

Compound	Target(s)
WH-4-023	SRC
KIN001-220	Aurora kinase
PD0332991	CDK4
KU55933	ATM
KIN001-244	PDK
XMD 8-85	ERK5
XMD 8-92	ERK5
SU11274*	MET
PHA-665752*	MET
BIBF-1120*	VEGFR, PDGFR, FGFR
SU11248	VEGFR1, PDGFR, KIT, FLT3

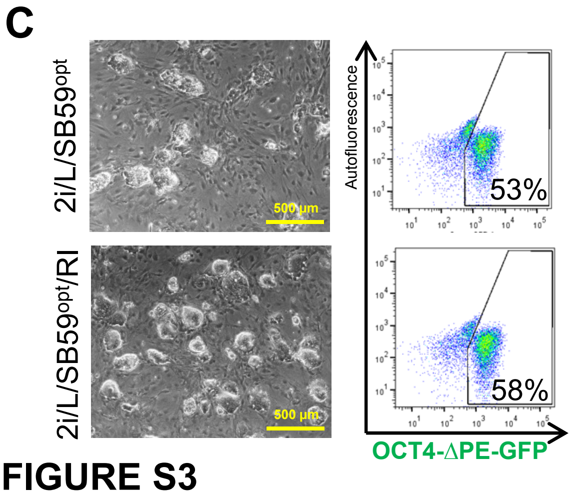

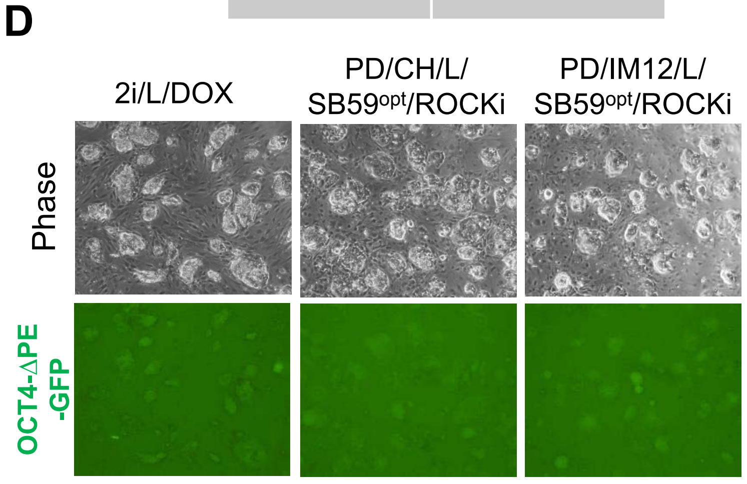

FIGURE S3

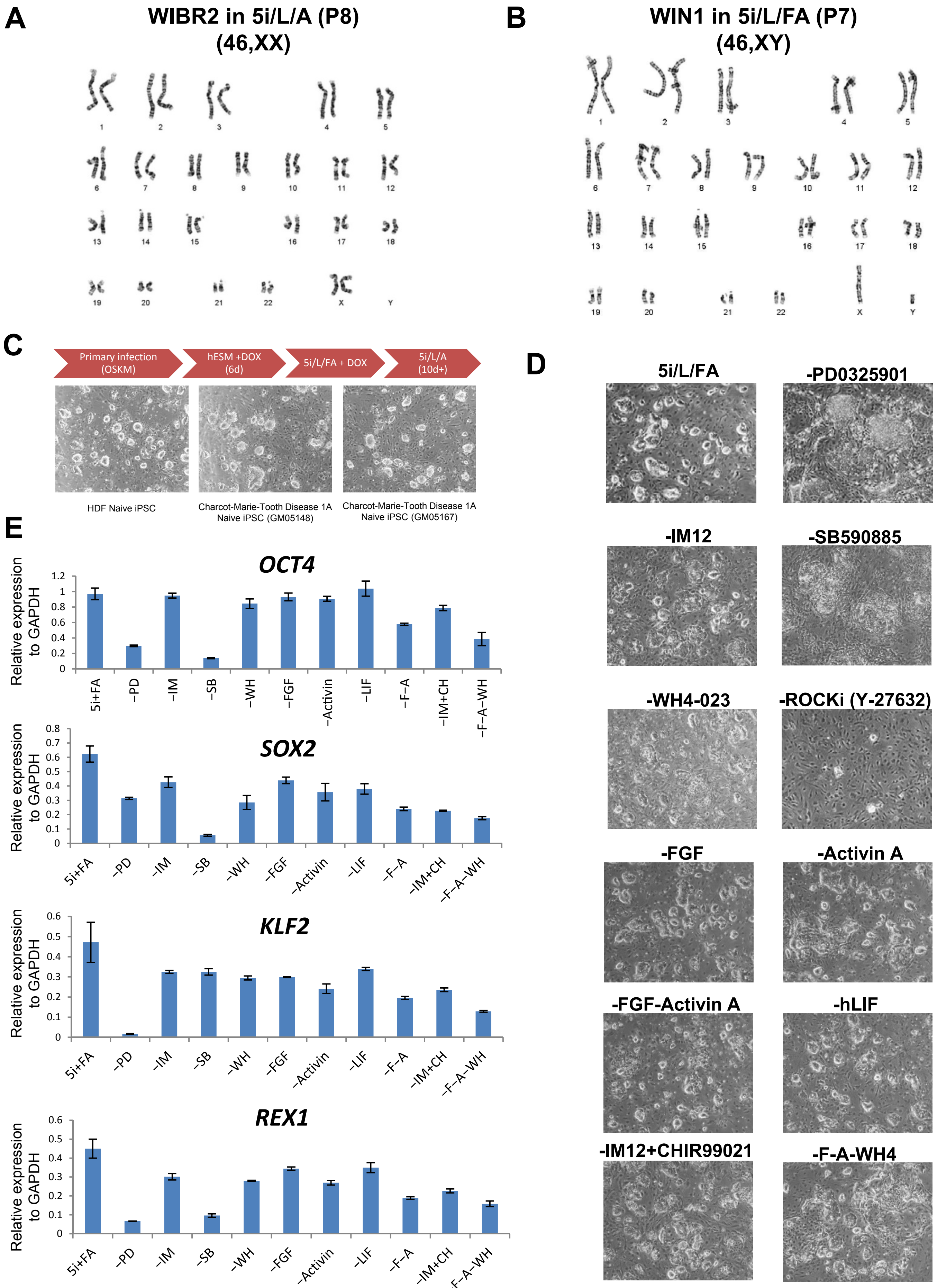

A
WIBR2 in 5i/L/A (P8)
(46,XX)
B
WIN1 in 5i/L/FA (P7)
(46,XY)
C
Primary infection (OSKM)
hESM +DOX (6d)
5i/L/FA + DOX
5i/L/A (10d+)
HDF Naive iPSC
Charcot-Marie-Tooth Disease 1A Naive iPSC (GM05148)
Charcot-Marie-Tooth Disease 1A Naive iPSC (GM05167)
D
5i/L/FA
-PD0325901
-IM12
-SB590885
-WH4-023
-ROCKi (Y-27632)
-FGF
-Activin A
-FGF-Activin A
-hLIF
-IM12+CHIR99021
-F-A-WH4
E
OCT4
SOX2
KLF2
REX1
Relative expression to GAPDH
5i+FA
–PD
–IM
–SB
–WH
–FGF
–Activin
–LIF
–F–A
–IM+CH
–F–A–WH

FIGURE S4

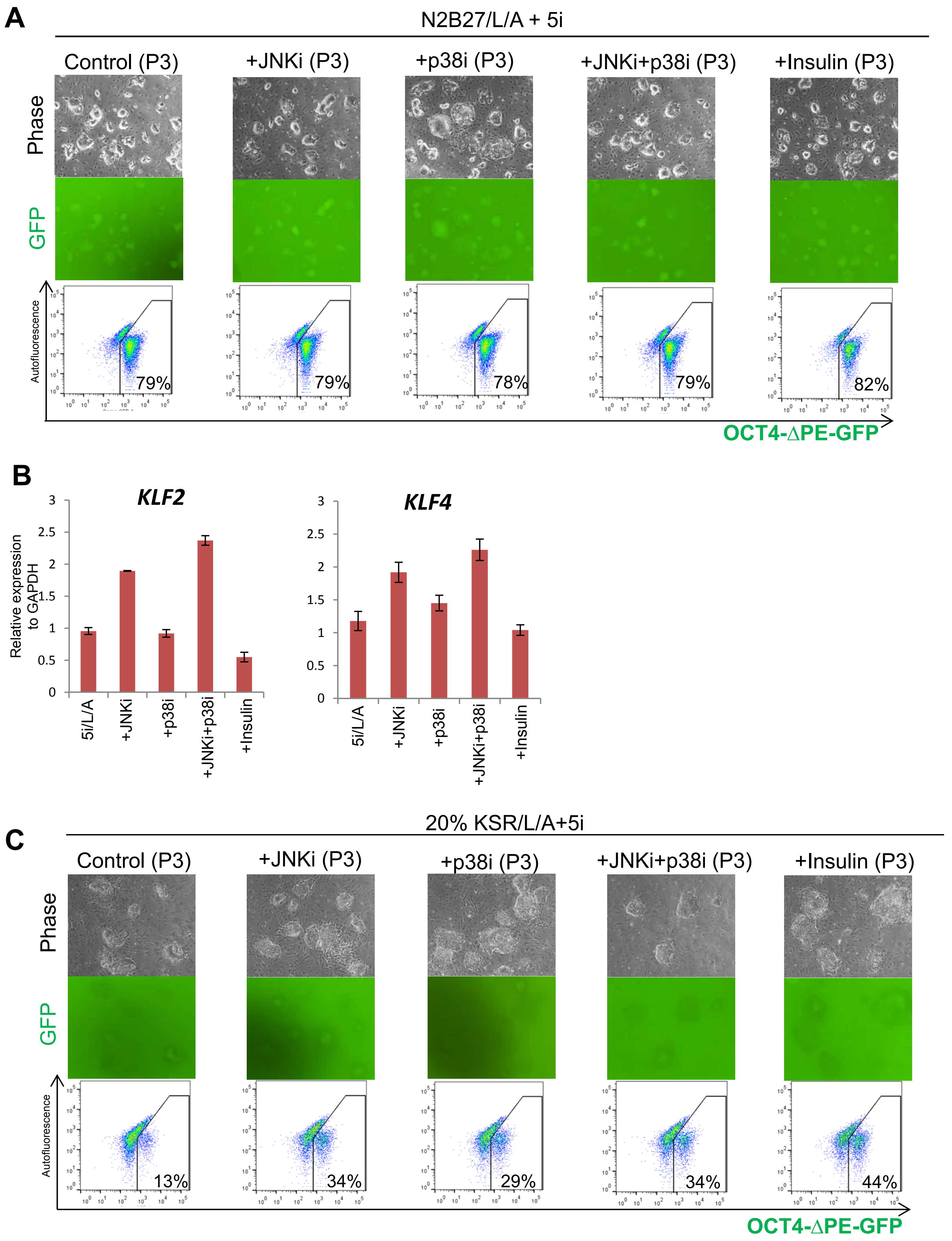
A
N2B27/L/A + 5i
Control (P3)
+JNKi (P3)
+p38i (P3)
+JNKi+p38i (P3)
+Insulin (P3)
Phase
GFP
Autofluorescence
79%
79%
78%
79%
82%
OCT4-ΔPE-GFP
B
KLF2
KLF4
Relative expression to GAPDH
5i/L/A
+JNKi
+p38i
+JNKi+p38i
+Insulin
C
20% KSR/L/A+5i
Control (P3)
+JNKi (P3)
+p38i (P3)
+JNKi+p38i (P3)
+Insulin (P3)
Phase
GFP
Autofluorescence
13%
34%
29%
34%
44%
OCT4-ΔPE-GFP

FIGURE S5

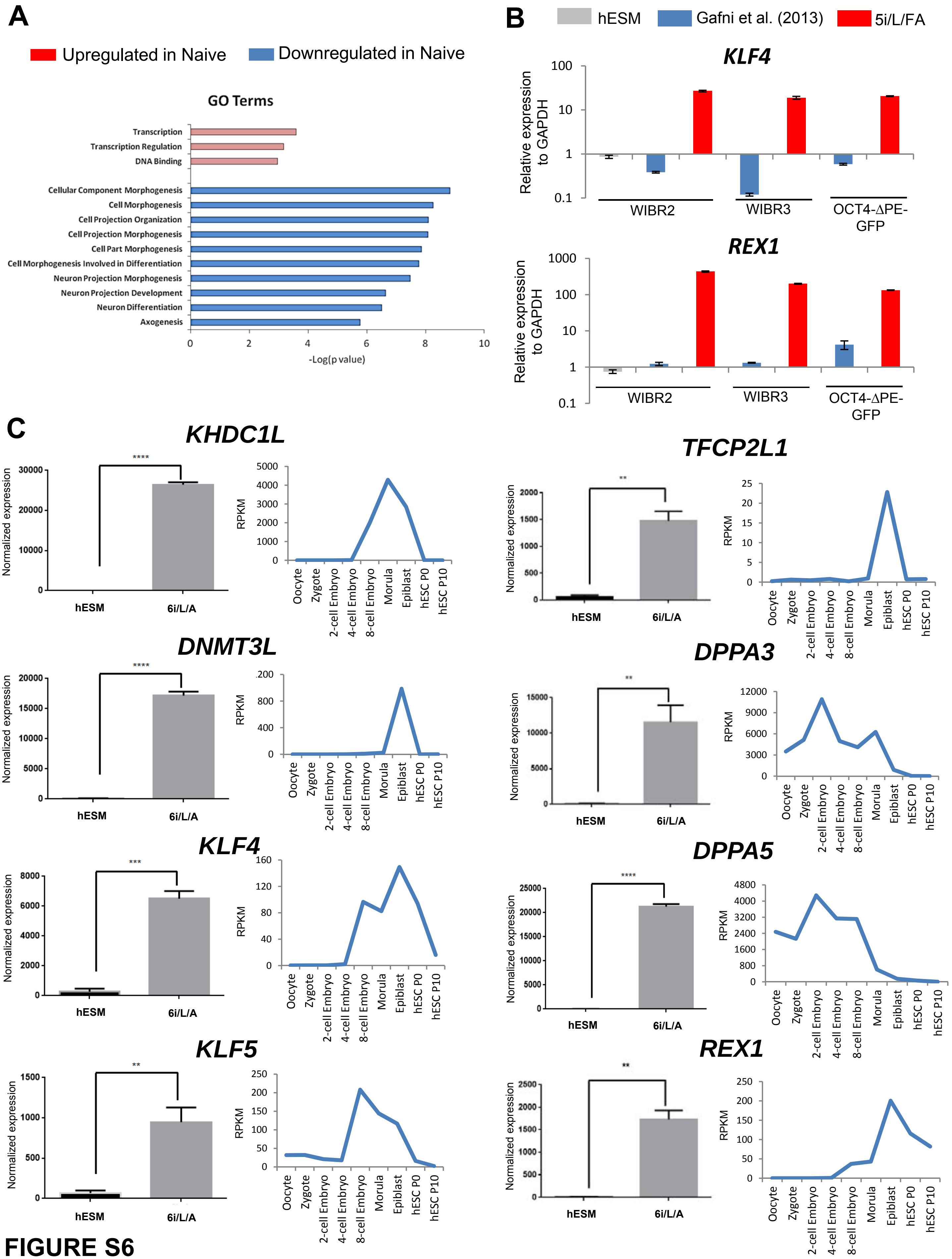
A
Upregulated in Naive
Downregulated in Naive
GO Terms
Transcription
Transcription Regulation
DNA Binding
Cellular Component Morphogenesis
Cell Morphogenesis
Cell Projection Organization
Cell Projection Morphogenesis
Cell Part Morphogenesis
Cell Morphogenesis Involved in Differentiation
Neuron Projection Morphogenesis
Neuron Projection Development
Neuron Differentiation
Axogenesis
-Log(p value)
B
hESM
Gafni et al. (2013)
5i/L/FA
KLF4
REX1
Relative expression to GAPDH
WIBR2
WIBR3
OCT4-ΔPE-GFP
C
KHDC1L
DNMT3L
KLF4
KLF5
TFCP2L1
DPPA3
DPPA5
REX1
Normalized expression
RPKM
hESM
6i/L/A
Oocyte
Zygote
2-cell Embryo
4-cell Embryo
8-cell Embryo
Morula
Epiblast
hESC P0
hESC P10

FIGURE S6

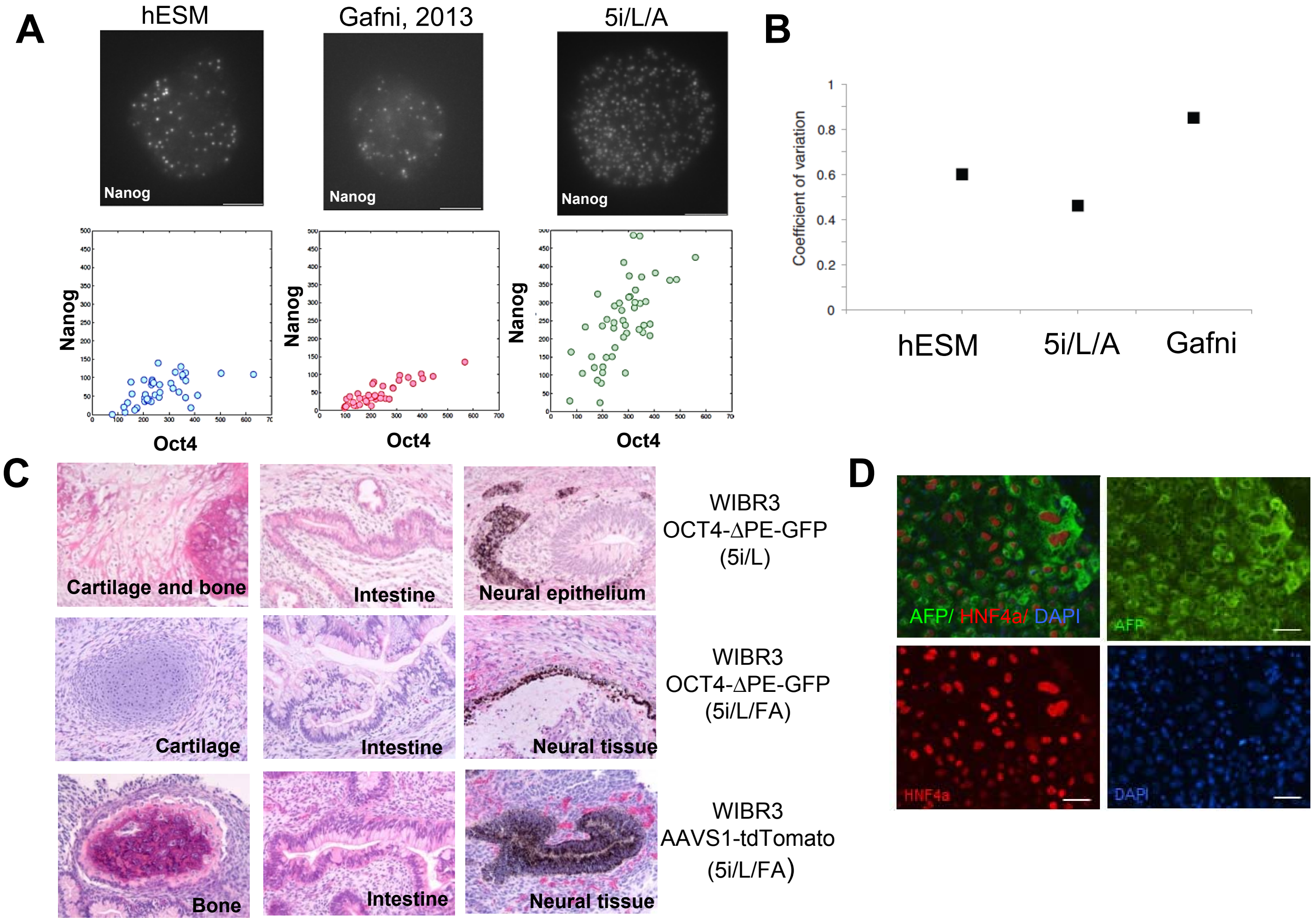

E

5i/L/FA medium Human-Mouse injections							
**Dev. Stage**	8-cell	8-cell	Morula	Morula	Blastocyst	Blastocyst	Total
**Cell line**	WIBR3 AAVS1-td Tomato	WIBR3 AAVS1-GFP	WIBR3 AAVS1-tdTomato	WIBR3 AAVS1-GFP	WIBR3 AAVS1-tdTomato	WIBR3 AAVS1-GFP	-
**Injected embryos**	245	363	92	60	50	50	860
**Embryos recovered at E10.5**	92	164*	47	28	11	26	368
**Positive embryos**	0	0	0	0	0	0	0
**Gafni et al. (2013) medium Human-Mouse injections**							
**Dev. stage**	8-cell	8-cell	8-cell	Morula	Morula	Blastocyst	Total
**Cell line**	C1 AAVS1-GFP on MEFs	C1 AAVS1-GFP on gel/vit	C1 AAVS1-GFP on matrigel	C1 AAVS1-GFP on MEFs	C1 AAVS1-GFP on gel/vit	C1 AAVS1-GFP on matrigel	-
**Injected embryos**	140	100	40	40	40	76	436
**Embryos recovered at E10.5**	96*	28	12	24	7**	28	195
**Positive embryos**	0	0	0	0	0	0	0

FIGURE S7

IV. Supplemental References

Boyer, L.A., Plath, K., Zeitlinger, J., Brambrink, T., Medeiros, L.A., Lee, T.I., Levine, S.S., Wernig, M., Tajonar, A., Ray, M.K*., et al.* (2006). Polycomb complexes repress developmental regulators in murine embryonic stem cells. Nature *441*, 349-353.

Brambrink, T., Foreman, R., Welstead, G.G., Lengner, C.J., Wernig, M., Suh, H., and Jaenisch, R. (2008). Sequential expression of pluripotency markers during direct reprogramming of mouse somatic cells. Cell stem cell *2*, 151-159.

Bruck, T., and Benvenisty, N. (2011). Meta-analysis of the heterogeneity of X chromosome inactivation in human pluripotent stem cells. Stem cell research *6*, 187-193.

Chan, Y.S., Goke, J., Ng, J.H., Lu, X., Gonzales, K.A., Tan, C.P., Tng, W.Q., Hong, Z.Z., Lim, Y.S., and Ng, H.H. (2013). Induction of a human pluripotent state with distinct regulatory circuitry that resembles preimplantation epiblast. Cell stem cell *13*, 663-675.

Costa, M., Dottori, M., Sourris, K., Jamshidi, P., Hatzistavrou, T., Davis, R., Azzola, L., Jackson, S., Lim, S., Pera, M*., et al.* (2007). A method for genetic modification of human embryonic stem cells using electroporation. Nat Protoc *2*, 792-796.

Faddah, D.A., Wang, H., Cheng, A.W., Katz, Y., Buganim, Y., and Jaenisch, R. (2013). Single-cell analysis reveals that expression of nanog is biallelic and equally variable as that of other pluripotency factors in mouse ESCs. Cell stem cell *13*, 23-29.

Gafni, O., Weinberger, L., Mansour, A.A., Manor, Y.S., Chomsky, E., Ben-Yosef, D., Kalma, Y., Viukov, S., Maza, I., Zviran, A*., et al.* (2013). Derivation of novel human ground state naive pluripotent stem cells. Nature *504*, 282-286.

Hockemeyer, D., Wang, H., Kiani, S., Lai, C.S., Gao, Q., Cassady, J.P., Cost, G.J., Zhang, L., Santiago, Y., Miller, J.C*., et al.* (2011). Genetic engineering of human pluripotent cells using TALE nucleases. Nature biotechnology *29*, 731-734.

Itzkovitz, S., Lyubimova, A., Blat, I.C., Maynard, M., van Es, J., Lees, J., Jacks, T., Clevers, H., and van Oudenaarden, A. (2011). Single-molecule transcript counting of stem-cell markers in the mouse intestine. Nat Cell Biol *14*, 106-114.

Langmead, B., Trapnell, C., Pop, M., and Salzberg, S.L. (2009). Ultrafast and memory-efficient alignment of short DNA sequences to the human genome. Genome biology *10*, R25.

Pruitt, K.D., Tatusova, T., and Maglott, D.R. (2007). NCBI reference sequences (RefSeq): a curated non-redundant sequence database of genomes, transcripts and proteins. Nucleic acids research *35*, D61-65.

Raj, A., Rifkin, S.A., Andersen, E., and van Oudenaarden, A. (2010). Variability in gene expression underlies incomplete penetrance. Nature *463*, 913-918.

Raj, A., van den Bogaard, P., Rifkin, S.A., van Oudenaarden, A., and Tyagi, S. (2008). Imaging individual mRNA molecules using multiple singly labeled probes. Nat Methods *5*, 877-879.

Si-Tayeb, K., Noto, F.K., Nagaoka, M., Li, J., Battle, M.A., Duris, C., North, P.E., Dalton, S., and Duncan, S.A. (2010). Highly efficient generation of human hepatocyte-like cells from induced pluripotent stem cells. Hepatology *51*, 297-305.

Smyth, G.K. (2004). Linear models and empirical bayes methods for assessing differential expression in microarray experiments. Statistical applications in genetics and molecular biology *3*, Article3.

Soldner, F., Hockemeyer, D., Beard, C., Gao, Q., Bell, G.W., Cook, E.G., Hargus, G., Blak, A., Cooper, O., Mitalipova, M.*, et al.* (2009). Parkinson's disease patient-derived induced pluripotent stem cells free of viral reprogramming factors. Cell *136*, 964-977.

Soldner, F., Laganiere, J., Cheng, A.W., Hockemeyer, D., Gao, Q., Alagappan, R., Khurana, V., Golbe, L.I., Myers, R.H., Lindquist, S.*, et al.* (2011). Generation of isogenic pluripotent stem cells differing exclusively at two early onset Parkinson point mutations. Cell *146*, 318-331.

Ware, C.B., Nelson, A.M., Mecham, B., Hesson, J., Zhou, W., Jonlin, E.C., Jimenez-Caliani, A.J., Deng, X., Cavanaugh, C., Cook, S.*, et al.* (2014). Derivation of naive human embryonic stem cells. Proc Natl Acad Sci U S A *111*, 4484-4489.

Xu, C., Jiang, J., Sottile, V., McWhir, J., Lebkowski, J., and Carpenter, M.K. (2004). Immortalized fibroblast-like cells derived from human embryonic stem cells support undifferentiated cell growth. Stem cells *22*, 972-980.

Yan, L., Yang, M., Guo, H., Yang, L., Wu, J., Li, R., Liu, P., Lian, Y., Zheng, X., Yan, J., *et al.* (2013). Single-cell RNA-Seq profiling of human preimplantation embryos and embryonic stem cells. Nature structural & molecular biology *20*, 1131-1139.

Zhang, Y., Liu, T., Meyer, C.A., Eeckhoute, J., Johnson, D.S., Bernstein, B.E., Nusbaum, C., Myers, R.M., Brown, M., Li, W., *et al.* (2008). Model-based analysis of ChIP-Seq (MACS). Genome biology *9*, R137.
